# Cadherin 1 negatively regulates SARS-CoV-2 infection in the olfactory epithelium

**DOI:** 10.1038/s44319-026-00880-8

**Published:** 2026-08-03

**Authors:** Weihao Li, Yuansen Shi, Xuewen Li, Meiqin Liu, Jinxia Liu, Zhen Qin, Haofeng Lin, Yuzhen Wang, Yiqun Yu

**Affiliations:** 1 ENT Institute and Department of Otorhinolaryngology, Eye & ENT Hospital, Fudan University, Shanghai, China; 2 Olfactory Disorder Diagnosis and Treatment Center, Eye & ENT Hospital, Fudan University, Shanghai, China; 3 Guangzhou Medical University, Guangzhou, China; 4 Guangzhou National Laboratory, Guangzhou International Bio Island, Guangzhou, China; 5 School of Life Sciences, Shanghai University, Shanghai, China; 6 The First Affiliated Hospital of Guangzhou Medical University, Guangzhou Medical University, Guangzhou, China

**Keywords:** Microbiology, Virology & Host Pathogen Interaction, Respiratory System, Structural Biology

## Abstract

Olfactory dysfunction is a major symptom of COVID-19 syndrome. The critical genes contributing to SARS-CoV-2 infection are not fully understood. Here, we identified Cadherin 1 (CDH1), a hub in the interaction network of viral entry-related genes in human and mouse olfactory epithelium (OE), is coexpressed with ACE2 in sustentacular cells. CDH1 overexpression attenuates pseudoviral and authentic SARS-CoV-2 infection in ACE2-expressing HEK293T cells and human OE organoids, whereas CDH1 downregulation promotes infection. Mechanistically, CDH1 interacts with ACE2 and competes with the Spike protein for ACE2 binding. Three residues in CDH1 (W59, N168, and Q197) are critical for CDH1-ACE2 binding, and mutations at these residues weaken the interaction and enhance pseudoviral infection. In the OE of deceased COVID-19 patients, cells with higher viral load exhibit lower CDH1 expression. This inverse correlation between CDH1 expression and SARS-CoV-2 load is validated in human bronchial epithelial cells, and in lung ciliated and secretory cells. Collectively, our findings establish CDH1 as a novel restriction factor for SARS-CoV-2 infection in the olfactory system, potentially offering a target for antiviral therapy.

## Synopsis

In this study, we identify CDH1 as a critical and novel restriction factor for SARS-CoV-2 infection in the olfactory system. This may offer an ideal target for antiviral therapy.

- CDH1 is a hub in the interaction network of viral entry-related genes in the olfactory epithelium.
- CDH1 overexpression attenuates pseudoviral and authentic SARS-CoV-2 infection, whereas CDH1 downregulation promotes infection.
- CDH1 interacts with ACE2 and competes with the Spike protein for ACE2 binding.
- Three residues in CDH1 (W59, N168, and Q197) are critical for the CDH1–ACE2 binding.
- Cells with higher viral load exhibit lower CDH1 expression in the olfactory epithelium of COVID-19 patients.

## Abstract (web summary)

In this study, we identify CDH1 as a critical and novel restriction factor for SARS-CoV-2 infection in the olfactory system. This may offer an ideal target for antiviral therapy.

## Graphical abstract

**Figure:**
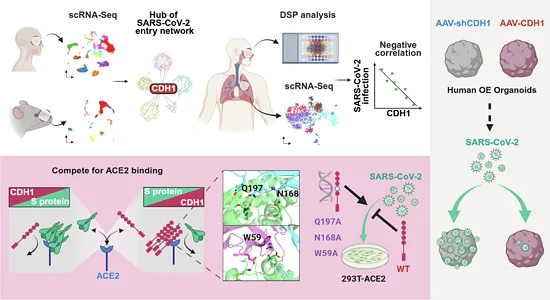

**Figure:**
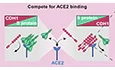

## Introduction

SARS-CoV-2 is the pandemic coronavirus responsible for COVID-19. Infection with SARS-CoV-2 frequently leads to olfactory dysfunction (Giacomelli et al, 2020; Spinato et al, 2020), and recovery from COVID-19-related anosmia typically occurs over weeks without medical intervention (Lee et al, 2020). A well-established mechanism underlying SARS-CoV-2 infection involves the interaction between the viral Spike (S) protein and angiotensin-converting enzyme 2 (ACE2) expressed on host cells. The transmembrane serine protease TMPRSS2 facilitates viral entry by cleaving the Spike protein, a critical step that enables binding to ACE2 (Hoffmann et al, 2020). In the mammalian olfactory epithelium (OE), non-neuronal cells such as sustentacular cells and horizontal basal cells (HBCs) express the SARS-CoV-2 entry factors ACE2 and TMPRSS2, whereas olfactory sensory neurons (OSNs) do not (Brann et al, 2020). These findings are supported by multiple studies demonstrating preferentially expression of ACE2 and TMPRSS2 in sustentacular cells but not in OSNs (Bilinska et al, 2020; Fodoulian et al, 2020), implicating sustentacular cells in viral entry and subsequent olfactory impairment. Further evidence indicates that in COVID-19 patients with acute smell loss, OMP^+^ mature OSNs, CK18^+^ sustentacular cells, and Iba1^+^ immune cells within the OE are infected, and viral replication is associated with local inflammation (de Melo et al, 2021). SARS-CoV-2 infection induces cell death and immune cell infiltration in the OE, triggers inflammatory responses, and downregulates olfactory receptor (OR) gene expression (Ye et al, 2021). Moreover, the virus can invade the central nervous system by crossing the neural–mucosal interface in the olfactory mucosa (Meinhardt et al, 2021). Despite these advances, how SARS-CoV-2 infection in the olfactory system is regulated remains poorly understood.

Previous reports have identified multiple factors that modulate SARS-CoV-2 infectivity. The viral Spike protein binds to cell surface Neuropilin-1 (NRP1) receptor on host cells (Daly et al, 2020), and NRP1 significantly enhances viral infectivity (Cantuti-Castelvetri et al, 2020). Additional mediators including cathepsin L, ADAM17, and heparan sulphate facilitate SARS-CoV-2 entry into pancreatic islets, either in conjunction with ACE2 or independently (Rangu et al, 2022). Moreover, SARS-CoV-2 has been shown to infect insulin-positive β cells both ex vivo and in vivo (Muller et al, 2021; Wu et al, 2021). The human glucose-regulated protein GRP78, a chaperone that translocates to the cell surface under cellular stress, binds to the SARS-CoV-2 Spike protein and is involved in viral entry (Johnson et al, 2024). In addition, ADAM10 and ADAM17 contribute to the priming of the Spike protein and promote viral entry into host cells (Jocher et al, 2022).

Cadherin 1 (CDH1) plays a critical role in cell cycle entry and cellular differentiation (Qiao et al, 2010). It has been reported that CDH1 expression is significantly downregulated in Caco-2 cells infected with SARS-CoV-2, compared to uninfected controls(Osman et al, 2022), suggesting that viral infection may negatively regulate CDH1 expression. Biering et al performed genome-wide CRISPR knockout and activation screens in human lung epithelial cell line Calu-3 and identified several host factors, including CDH1, that influence SARS-CoV-2 infectivity (Biering et al, 2022). However, whether CDH1 is expressed in the OE and whether it is involved in SARS-CoV-2 infection in olfactory tissues remain unknown. In this study, we identify CDH1 as a critical regulator for SARS-CoV-2 infection. CDH1 expression correlates with known SARS-CoV-2 infection-related genes in the OE. Overexpression of CDH1 reduces pseudoviral and SARS-CoV-2 infectivity, while CDH1 knockdown enhances viral infection. Mechanistically, CDH1 interacts with ACE2 and competes with the viral Spike protein for ACE2 binding. Importantly, CDH1 expression is negatively associated with viral load not only in olfactory epithelial cells but also in bronchial epithelial cells and lung cells from COVID-19 patients. Collectively, our findings establish CDH1 as an important regulator of SARS-CoV-2 infection and highlight its potential as a target for antiviral intervention.

## Results

### CDH1 is associated with SARS-CoV-2 infection-related genes in the OE

**Figure 1:**
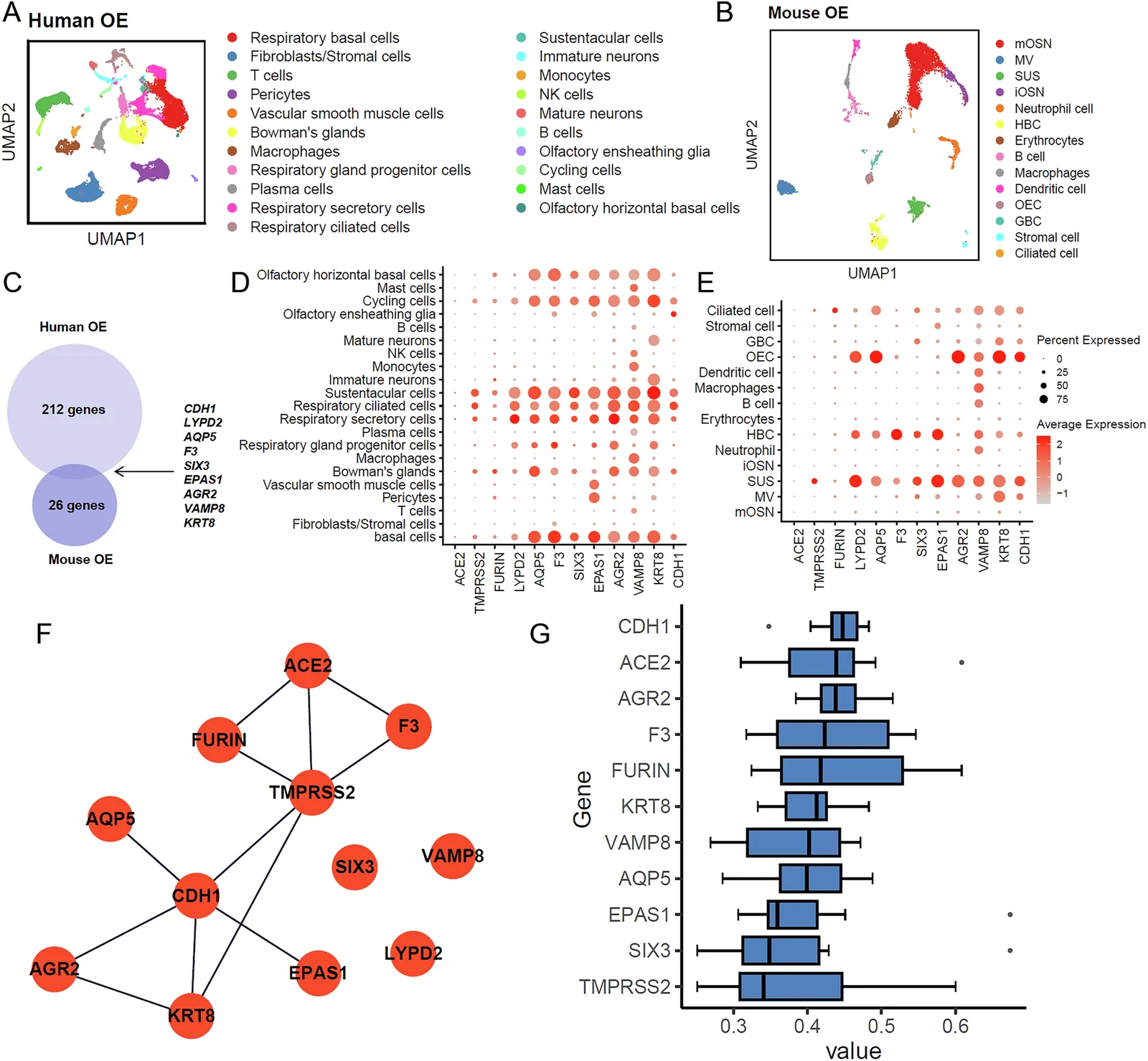
CDH1 is associated with SARS-CoV-2 infection genes in the OE. (**A**, **B**) UMAP plot showing various cell types in the human (**A**) and mouse (**B**) OE tissues. (**C**) Venn plot showing genes related with ACE2/FURIN/TMPRSS2 shared between human and mouse OE. (**D**, **E**) Dot plot showing expression of nine overlapped genes related with ACE2/FURIN/TMPRSS2 in various cell types of the human (**D**) and mouse (**E**) OE. (**F**) Interaction network of SARS-CoV-2 infection genes constructed by STRING database. (**G**) GOSemSim showing semantic similarity among nine overlapped ACE2/FURIN/TMPRSS2-related genes. The centre line represented the median (50th percentile), and the bounds of the box indicated the 25th and 75th percentiles (interquartile range, IQR). The whiskers extended to the minima and maxima values within 1.5 × IQR from the box limits. Individual outlier data points were overlaid as dots. Each box plot displayed the GO semantic similarity scores between the indicated gene and the other 10 genes, *n* = 10 genes.

To investigate CDH1 expression in the normal OE, we analyzed previously published scRNA-Seq data from human (Durante et al, 2020b; Data ref: Durante et al, 2020a) and mouse (Tan et al, 2020b; Data ref: Tan et al, 2020a) OE. UMAP plot revealed distinct cell populations in both human and mouse OE (Fig. 1A,B). CDH1 was expressed in both human and mouse OE. In human OE, CDH1 was co-expressed with ACE2, FURIN, and TMPRSS2 in several cell subtypes, including sustentacular (Sus) cells, respiratory basal cells (RBCs), respiratory ciliated cells, and respiratory secretory cells (Appendix Fig. S1A,B). In mouse OE, CDH1 was co-expressed with FURIN and TMPRSS2 mainly in Sus cells, HBCs, olfactory ensheathing cells (OECs), and ciliated cells (Appendix Fig. S1C,D). The percentage of cells co-expressing CDH1, ACE2, FURIN, and TMPRSS2 across six human OE cell types ranged from 0.086 to 1.011%, while the percentage of cells co-expressing CDH1, FURIN, and TMPRSS2 across five mouse OE cell types ranged from 0.141 to 8%. We next identified genes correlated with ACE2, FURIN, and TMPRSS2, three key factors for SARS-CoV-2 infection, in human and mouse OE. We identified 212 and 26 correlated genes in human and mouse, respectively. Among them, nine genes including CDH1, LYPD2, AQP5, F3, SIX3, EPAS1, AGR2, VAMP8, and KRT8 were overlapping between human and mouse OE (Fig. 1C). In human OE, these nine genes were predominantly expressed in HBCs, cycling cells, Sus cells, and multiple respiratory cell types, including ciliated cells, secretory cells, and RBCs (Fig. 1D). In mouse OE, these genes were enriched in HBCs, OECs, ciliated cells, and Sus cells (Fig. 1E). Protein–protein interaction analysis using the STRING database placed CDH1 at a central node within this network (Fig. 1F). Furthermore, GOSemSim analysis of semantic similarity in GO annotations revealed that CDH1 shared high functional similarity with the other overlapping genes (Fig. 1G). Collectively, our findings identify a conserved set of genes associated with SARS-CoV-2 entry and infection in human and mouse OE, with CDH1 potentially playing a central role in this gene network.

### Negative correlation between CDH1 expression and viral load in olfactory epithelial cells

**Figure 2:**
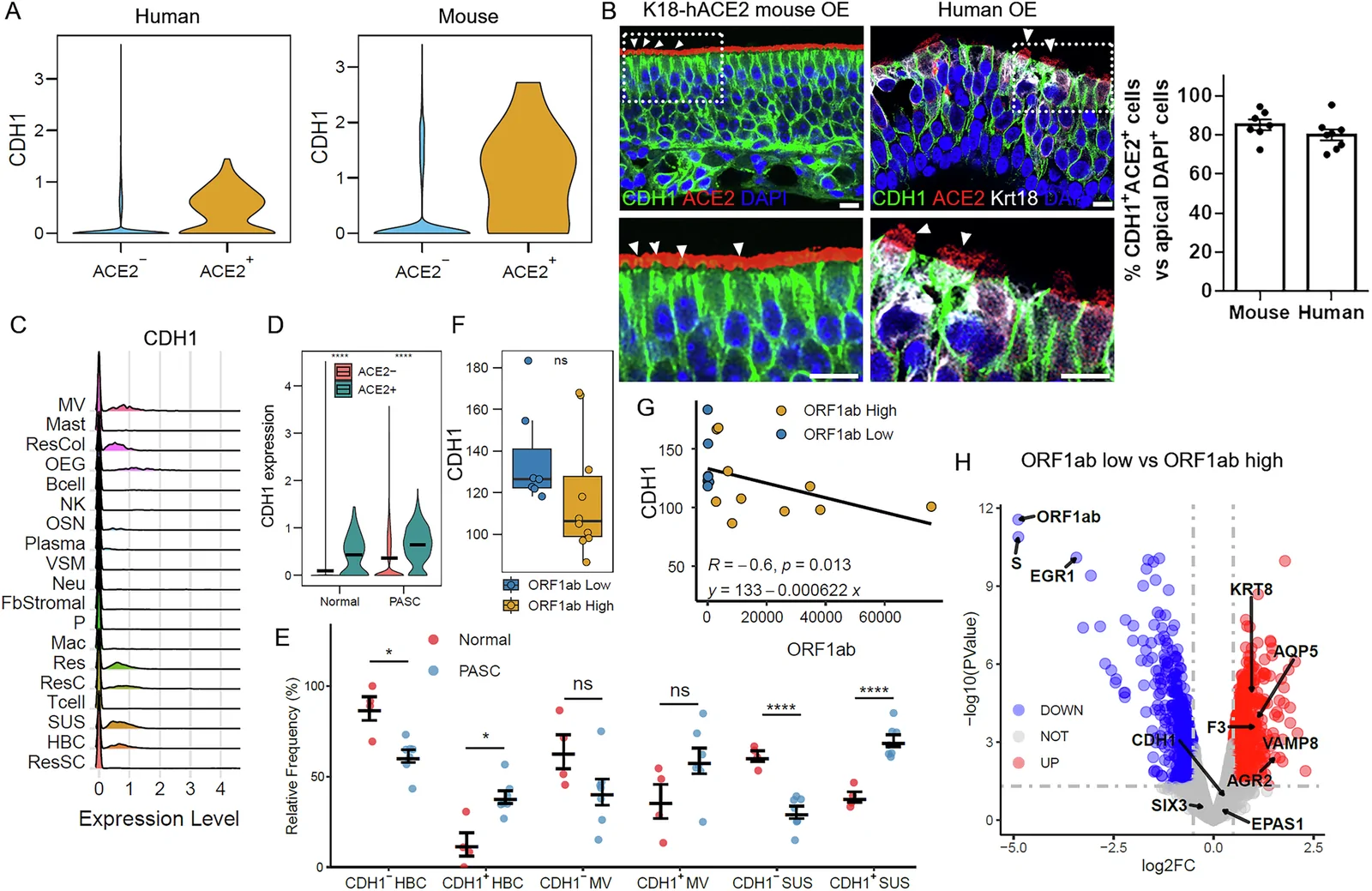
Correlation between CDH1 expression and SARS-CoV-2 loads in the OE of COVID-19 patients. (**A**) Violin plots showing CDH1 expression in ACE2^−^ and ACE2^+^ cells of normal human and mouse OE. Human: *n* = 4 patients, mouse: *n* = 4 animals. (**B**) Confocal images and quantification of CDH1^+^ and ACE2^+^ cells in K18-hACE2 mouse and human OE. White arrowheads denoted co-expression of CDH1 and ACE2. *n* = 7 and 8 sections from three technical replicates. (**C**) CDH1 expression in different cell types of the OE from patients with PASC. (**D**) Violin plot showing CDH1 expression in ACE2^-^ and ACE2^+^ cells of the OE from uninfected individuals and patients with PASC. Normal: ****P = 1.14E-134, *n* = 4 patients; PASC: ****P = 1.75E-68, *n* = 6 patients. (**E**) Percentages of CDH1^−^ and CDH1^+^ Sus cells, HBCs, MV cells in the OE from uninfected individuals and patients with PASC. *P = 0.0174 (CDH1^−^ HBC), *P* = 0.0661 (CDH1^+^ HBC), *P* = 0.106 (CDH1^−^ and CDH1^+^ MV), ****P < 0.0001 (CDH1^−^ and CDH1^+^ SUS). *n* = 4 (Normal) and 7 (COVIDPASC). (**F**) CDH1 expression in OE cells with high and low ORF1ab expression from deceased COVID-19 patients. ns: *P* = 0.0878. The centre line represented the median (50th percentile), and the bounds of the box indicated the 25th and 75th percentiles. The whiskers extended to the minima and maxima values within 1.5 × IQR from the box limits. Individual data points were overlaid as dots. ORF1ab Low: *n* = 7, ORF1ab High: *n* = 10. (**G**) A correlation analysis between CDH1 expression and ORF1ab expression in the OE from deceased COVID-19 patients. Statistical significance of the correlation was evaluated using Spearman’s rank correlation test. (**H**) Volcano plot showing downregulated (blue) and upregulated (red) genes in ORF1ab^low^ OE region than ORF1ab^high^ region. Differential expression analysis was performed using the likelihood ratio test (LRT) based on a negative binomial generalized linear model (GLM), implemented via the edgeR package. Significance was determined by unpaired *t* test with Benjamini & Hochberg adjustment in (**E**) and unpaired two-samples Wilcoxon rank-sum test in (**D**, **F**). ns not significant. Data were presented as mean ± SEM. Scale bars, 10 μm. Source data are available online for this figure.

We next investigated whether CDH1 is involved in SARS-CoV-2 infection. Analysis of scRNA-Seq data from mouse and human OE [GSE146043 from (Tan et al, 2020b; Data ref: Tan et al, 2020a) and GSE139522 from (Durante et al, 2020b; Data ref: Durante et al, 2020a)] revealed that ACE2^+^ cells in both human and mouse OE exhibited higher CDH1 expression levels compared to ACE2^-^ cells (Fig. 2A). Immunostaining confirmed co-expression of ACE2 and CDH1 in the surface layer of the K18-hACE2 mouse OE and in the apical Krt18^+^ Sus cells of the human OE (arrowheads in Fig. 2B), and quantification analysis indicated that 85 ± 3% and 80 ± 3% of apical cells expressed both ACE2 and CDH1 in the mouse and human OE, respectively (Fig. 2B). To elucidate the correlation between CDH1 expression and SARS-CoV-2 infection in the human OE, we analyzed published scRNA-Seq data from olfactory epithelial biopsies obtained from patients with post–COVID-19 hyposmia/anosmia at 4–16 months post infection with the prototypic SARS-CoV-2 strain, as well as from normosmic individuals with no history of COVID-19 [GSE201620 from (Finlay et al, 2022b; Data ref: Finlay et al, 2022a)]. We also examined spatial whole-transcriptome profiling data from olfactory cleft mucosa of deceased COVID-19 patients infected with the B.1.1.7/Alpha variant, collected shortly after death [GSE176080 from (Khan et al, 2021b; Data ref: Khan et al, 2021a)]. CDH1 was expressed in several cell subtypes of the patient OE, including microvilli (MV) cells, respiratory cells (Res), Sus cells, respiratory columnar cells (ResCol) and HBCs (Fig. 2C). In both normal controls and patients with post-acute sequelae of COVID-19 (PASC), CDH1 expression levels were significantly higher in ACE2^+^ cells compared to ACE2^-^ cells (Fig. 2D). The proportion of CDH1^+^ cells among Sus cells, and HBCs was significantly higher in PASC patients compared to normal OE (Fig. 2E). Conversely, COVID-19 infection reduced the ratio of CDH1^-^ cells in these populations (Fig. 2E). In the OE from deceased COVID-19 patients, cells with high Orf1ab expression (Orf1ab^high^) showed reduced CDH1 expression compared to cells in Orf1ab^low^ region, although this difference did not reach statistical significance (Fig. 2F). Correlation analysis revealed that CDH1 expression was negatively correlated with viral load in the OE of deceased COVID-19 patients, across cells with either high or low viral load (Fig. 2G). Compared to Orf1ab^high^ cells, Orf1ab^low^ cells exhibited higher expression levels of several ACE2/FURIN/TMPRSS2-associated genes, including KRT8, AQP5, VAMP8, AGR2, and F3 (Fig. 2H). In contrast, the transcriptional factor EGR1, which has been implicated in alleviating OE aging (Li et al, 2024), was downregulated in low viral load cells compared to those with high Orf1ab expression (Fig. 2H). To determine whether SARS-CoV-2 infection directly affects CDH1 expression, we measured CDH1 levels in infected OE organoids derived from Krt18-ACE2 mice and humans. In both infected organoid models, SARS-CoV-2 RNA copy numbers were significantly higher than in mock-infected controls (Appendix Fig. S2A,C). CDH1 expression showed no significant change in infected mouse organoids compared to mock controls by quantitative PCR (Appendix Fig. S2B). Infected human OE organoids also did not show significant difference in CDH1 expression at 3 dpi compared to uninfected control by bulk RNA-Seq analysis (Appendix Fig. S2D). By contrast, infection led to upregulation of CDH1 in human organoids at 6 dpi (Appendix Fig. S2E), and upregulation of CDH1 was also in olfactory epithelial cells of patients with PASC compared to normal control (Appendix Fig. S2F). This suggests that SARS-CoV-2 infection does not reduce CDH1 expression, whereas continuous infection may offset this expression. Collectively, CDH1 is enriched in ACE2^+^ olfactory epithelial cells, while its expression is negatively correlated with SARS-CoV-2 viral load in the OE.

### CDH1 overexpression inhibits pseudoviral and SARS-CoV-2 infection

To further investigate whether CDH1 regulates SARS-CoV-2 infection, we transiently overexpressed the full length of CDH1 in HEK293T cells stably expressing human ACE2 (293T-ACE2). All four CDH1 transcript variants were individually expressed in 293T-ACE2 cells, and their expression was visualized via mCherry fluorescence and immunostaining against CDH1. The vast majority of mCherry^+^ cells showed robust CDH1 expression (v1: 97 ± 2%, v2: 97 ± 3%, v3: 97 ± 2%, v4: 97 ± 1%, Appendix Fig. S3A,B). The relative intensity of CDH1 staining was significantly higher in mCherry^+^ cells compared to mCherry^–^ cells (Appendix Fig. S3C). These data suggest that mCherry signals represent the expression of CDH1. Moreover, ACE2 expression was not significantly affected by CDH1 overexpression, since intensity of ACE2 expression was not significantly different between mCherry^-^ and mCherry^+^ cells (Appendix Fig. S4A,B). Immunostaining data validated colocalization of CDH1 and ACE2 in HEK293T cells transiently overexpressing CDH1 variants (Appendix Fig. S4C,D).

**Figure 3:**
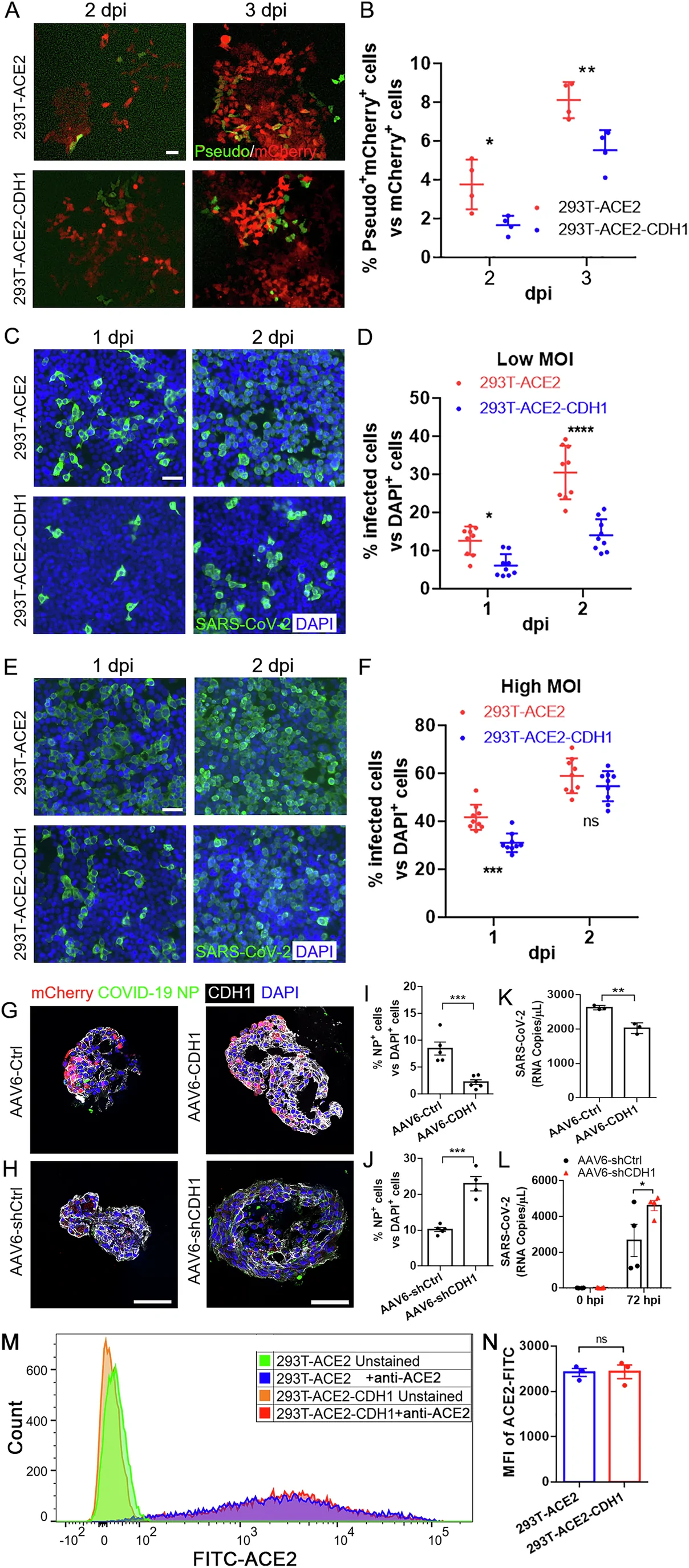
CDH1 overexpression inhibits SARS-CoV-2 infection in HEK293T cells expressing human ACE2 and human OE organoids. (**A**) Images of pseudoviral infection in HEK293T cells expressing human ACE2 (293T-ACE2 cells) and 293T-ACE2 cells stably expressing CDH1 variant 1 (293T-ACE2-CDH1 cells) at 2 and 3 dpi (days post infection). (**B**) Quantitative analysis on the ratios of pseudovirus-infected 293T-ACE2 and 293T-ACE2-CDH1 cells (EGFP^+^mCherry^+^) in all mCherry^+^ cells. *P = 0.0207, **P = 0.0056. *n* = 4 per group. (**C**) Images of SARS-CoV-2-infected (GFP^+^) cells at low dose (MOI = 0.0002) at 1 and 2 dpi. (**D**) Quantitative analysis on ratios of SARS-CoV-2-infected cells at 1 and 2 dpi with low MOI, *P = 0.0129, ****P < 0.0001, *n* = 9 per group. (**E**) Images of SARS-CoV-2-infected (GFP^+^) cells at high dose (MOI = 0.002) at 1 and 2 dpi. (**F**) Quantitative analysis on ratios of SARS-CoV-2-infected cells at 1 and 2 dpi with high MOI, ***P = 0.001, ns: *P* = 0.2311, *n* = 9 per group. (**G**, **H**) Confocal images of mCherry^+^ (AAV infection), COVID-19 NP^+^ (SARS-CoV-2 infection), CDH1^+^ cells in human OE organoids infected with AAV6-Ctrl (Control group) and AAV6-CDH1 (CDH1 overexpression) (**G**), AAV6-shCtrl and AAV6-shCDH1 (CDH1 downregulation) (**H**). (**I**, **J**) Quantification for the ratio of NP^+^ cells in organoids infected with AAV6-Ctrl and AAV6-CDH1 (**I**), AAV6-shCtrl and AAV6-shCDH1 (**J**). ***P = 0.0006 (**I**), ***P = 0.0003 (**J**). *n* = 5 and 6 biological replicates (**I**), *n* = 5 and 4 (**J**). Measurement of SARS-CoV-2 RNA copies in human OE organoids with CDH1 overexpression (**K**) and CDH1 downregulation (**L**). (**K**) *n* = 3 per group, **P = 0.0035; (**L**) *n* = 4 per group, *P = 0.0262. (**M**) Flow cytometry histograms showing ACE2 surface abundance on 293T-ACE2 and 293T-ACE2-CDH1 cells. ACE2 expression levels were detected using FITC labeling. (**N**) Quantitative bar graph showing the mean fluorescence intensity (MFI) of surface FITC-ACE2. *n* = 3 biological replicates. *P* = 0.9347. The statistical significance was determined by two-way ANOVA with Sidak’s multiple comparisons test in (**B**, **D**, **F**, **L**), and by unpaired *t* test in (**I**–**K**, **N**). ns, not significant. Scale bars, 100 µm. Data were presented as mean ± SEM. Source data are available online for this figure.

Using SARS-CoV-2 pseudovirus to assess viral infection, we observed that transient expression of CDH1 variant 1 (CDH1-v1) significantly reduced the proportion of pseudovirus-infected cells (EGFP^+^mCherry^+^) at 24 h post infection. In contrast, overexpression of variant 3 and 4 did not markedly alter the infection rate compared to control plasmid (Appendix Fig. S5). These findings suggest that CDH1-v1 overexpression specifically attenuates pseudoviral entry in 293T-ACE2 cells. We next generated 293T-ACE2 cells stably overexpressing CDH1-v1 (293T-ACE2-CDH1). Compared to control cells, stable CDH1 overexpression led to a significantly reduction in the number of pseudovirus-infected cells (MOI = 0.005) at both 2 and 3 days post infection (dpi), with decreases of 55 ± 6% and 32 ± 6% in the percentage of EGFP^+^mCherry^+^ cells, respectively (Fig. 3A,B). To extend these observations to authentic SARS-CoV-2, we infected 293T-ACE2 and 293T-ACE2-CDH1 cells at low (0.0002) and high (0.002) MOIs. At low MOI, the frequency of SARS-CoV-2-infected cells was reduced by 46 ± 7% at 1 dpi and 52 ± 4% at 2 dpi in CDH1-overexpressing cells relative to controls (Fig. 3C,D). At high MOI, infection rates were decreased by 25 ± 3% at 1 dpi and 7 ± 4% at 2 dpi (Fig. 3E,F).

To determine whether CDH1 also modulates SARS-CoV-2 infection in a more physiologically relevant model, we used human OE organoids. CDH1 expression was modulated via AAV-mediated gene transfer, and up- and downregulation was confirmed by quantitative PCR (Appendix Fig. S6). Following SARS-CoV-2 infection, immunostaining for viral nucleoprotein (NP) revealed markedly reduced NP signals in organoids overexpressing CDH1 (AAV6-CDH1) compared to controls (AAV6-Control), with 73 ± 5% reduction in the percentage of COVID-19 NP^+^ cells (Fig. 3G,I). Conversely, CDH1 knockdown enhanced NP expression, with 128 ± 13% increase in the percentage of COVID-19 NP^+^ cells (Fig. 3H,J). Consistently, SARS-CoV-2 RNA copy numbers were significantly lower in CDH1-overexpressing organoids (Fig. 3K) and significantly higher upon CDH1 knockdown (Fig. 3L).

To further confirm if CDH1 overexpression affects ACE2 surface expression, we performed flow cytometry and found that no significant difference in the mean fluorescence intensity (MFI) of ACE2 was observed between the HEK293T-ACE2 control and HEK293T-ACE2-CDH1 cells (Fig. 3M,N), indicating that CDH1 overexpression does not interfere with the abundance of membrane ACE2. We then assessed the membrane binding of three coronavirus Spike proteins in HEK293T-ACE2 cells, with AAV-mediated CDH1 overexpression and knockout. Flow cytometry revealed that HCoV-NL63 Spike protein with low affinity exhibited minimal binding at threshold levels in both control and CDH1 regulation groups (Appendix Fig. S7). By comparison, the binding efficiency of SARS-lineage Spike protein binding negatively correlated with CDH1 expression abundance. AAV-mediated CDH1 overexpression suppressed the membrane binding of both SARS-lineage Spike proteins. Compared to the controls, the binding rate of SARS-CoV-1 Spike protein decreased from 19.3 to 11.2%, and SARS-CoV-2 Spike binding decreased from 21.2 to 13.5% (Appendix Fig. S7A,B). Moreover, AAV-targeted knockout of endogenous CDH1 increased Spike protein binding. Compared to the control group, SARS-CoV-1 Spike binding was increased from 22.3 to 26.1%, and SARS-CoV-2 Spike binding was enhanced from 10.8 to 15.1% (Appendix Fig. S7C,D). These data demonstrated that cell-surface CDH1 serves as a restriction factor that hinders SARS-lineage Spike protein binding, without altering membrane abundance of ACE2.

Taken together, these results demonstrate that CDH1 overexpression attenuates SARS-CoV-2 infection and Spike protein binding, while its downregulation enhances these processes. These findings highlight a regulatory role for CDH1 expression levels in modulating susceptibility to SARS-CoV-2 infection.

### The interaction between ACE2 and CDH1

**Figure 4:**
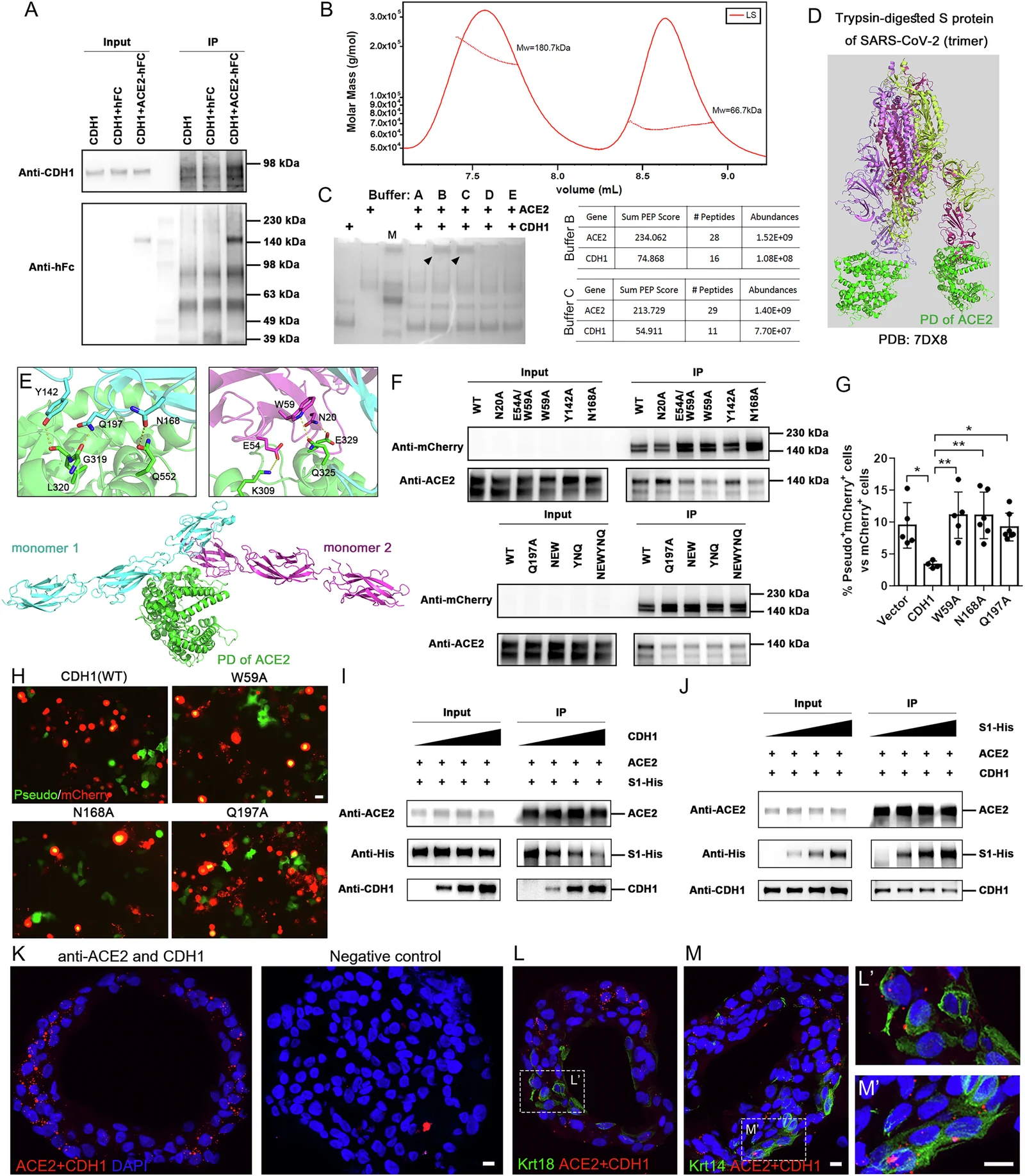
Interaction between ACE2 and CDH1. (**A**) Immunoprecipitation of CDH1 with ACE2-hFc fusion protein. Purified recombinant proteins were subjected to immunoprecipitation using Protein A/G beads, followed by immunoblotting with anti-CDH1 and anti-hFc antibodies. (**B**) MALS analysis of ACE2-CDH1 complex eluted from an SEC column. The absolute molar mass versus elution volume shows two peaks at 180.7 kDa and 66.7 kDa. (**C**) Native PAGE analysis of CDH1–ACE2 complex formation under different buffer conditions. Buffer A: PBS, 6% Trehalose, pH 7.4; Buffer B: 25 mM Tris-HCl, 150 mM NaCl, 1 mM EDTA, 1% NP-40, 5% Glycerin, pH 7.5; Buffer C: 25 mM Tris-HCl, 150 mM NaCl, 1 mM EDTA, 1% Triton X-100, 5% Glycerin, pH 7.5; Buffer D: 0.1 M Glycine, 1 M Tris, pH 7.5; Buffer E: 50 mM Tris-HCl, 150 mM NaCl, pH 7.5. M: Marker. Black arrowheads marked bands corresponding to potential CDH1–ACE2 complex, subjected to LC-MS/MS analysis. (**D**) Ribbon diagrams showing Trypsin-digested S protein of SARS-CoV-2 bound with peptidase domain (PD) of ACE2. PDB codes of shown structure: 7DX8. (**E**) Molecular docking prediction of the CDH1 and PD of ACE2 binding model. Squared images showed amino acid residues contributing to interactions between ACE2 and CDH1. (**F**) Co-IP assay of ACE2 recombinant protein and mutated CDH1 protein, immunoprecipitated with anti-mCherry magnetic beads, followed by immunoblotting with ACE2 and mCherry antibodies. The wild-type CDH1 was used as a control. (**G**) Quantification of ratios of GFP^+^mCherry^+^ infected cells versus mCherry^+^ cells. *P = 0.0205 (CDH1 vs. vector), **P = 0.0033 (CDH1 vs. W59A), **P = 0.0024 (CDH1 vs. N168A), *P = 0.0174 (CDH1 vs. Q197A). Data were presented as mean ± SD. *n* = 5, 4, 5, 6, 7 technical replicates for vector, CDH1, W59A, N168A, Q197A group. (**H**) Microscopic images of Pseudoviral^+^ (GFP^+^) and mCherry^+^ (representing WT and mutated CDH1) in 293T-ACE2 cells. (**I**) CDH1 competes with Spike S1 for binding to ACE2. ACE2 was incubated with S1-His and increasing concentrations of CDH1 (0, 300, 1500, or 3000 ng), followed by immunoprecipitation with Streptavidin magnetic beads. The precipitated complexes were analyzed by immunoblotting using antibodies against ACE2, His, and CDH1. (**J**) Spike S1 displaces CDH1 from ACE2 in a dose-dependent manner. Purified recombinant ACE2 and CDH1 were incubated with graded concentrations of S1-His (0, 300, 600, or 1200 ng) and subjected to immunoprecipitation with Streptavidin magnetic beads. The resulting complexes were examined by immunoblotting with antibodies against ACE2, His, and CDH1. (**K**–**M**) Confocal images of PLA data showing the interaction between CDH1 and ACE2 in human OE organoids (**K**), with immunostaining against Krt18 (**L**) and Krt14 (**M**). The dashed squares in (**L**, **M**) were magnified as (**L’**, **M’**). The statistical significance was determined by one-way ANOVA with Dunnett’s multiple comparisons test in (**G**). Scale bars, 20 µm in (**H**), 10 µm in (**K**–**M’**). Source data are available online for this figure.

We next investigated whether CDH1 directly interacts with ACE2. Co-immunoprecipitation followed by immunoblotting using antibodies against CDH1 and the hFc tag showed that recombinant human ACE2 fused to a hFC epitope (ACE2-hFc), but not hFc alone, effectively pulled down CDH1 (Fig. 4A; Appendix Fig. S8A). Although Protein A/G beads exhibited some non-specific binding to CDH1, the inclusion of hFc-tagged proteins allowed specific pull-down via the hFc moiety, supporting the specificity of the observed CDH1-ACE2 interaction. To further validate this interaction, we performed size-exclusion chromatography coupled with multi-angle light scattering (SEC-MALS) using ACE2-hFc and recombinant human CDH1. A mixture of both protein at 1 mg/mL was subjected to SEC-MALS to determine the oligomeric state of the complex in solution. The first peak exhibited an average molecular mass of 180.7 kDa, consistent with the predicted size of an ACE2-CDH1 complex, while the second peak corresponded to a molecular mass of 66.7 kDa, matching that of a CDH1 monomer (Fig. 4B). Native-PAGE analysis further confirmed the formation of a stable complex, with high-molecular-weight bands appearing near the top of the gel under two different buffer conditions (Buffer B and C), suggesting the presence of a CDH1–ACE2 complex under native conditions (Fig. 4C). To confirm the identity of the proteins in these bands, the corresponding gel regions were excised and analyzed by mass spectrometry, which detected both CDH1 and ACE2, thereby supporting the formation of a specific and stable complex between the two proteins (Fig. 4C). The Spike (S) protein of SARS-CoV-2 is known to utilize ACE2 as cell entry receptor and to facilitate viral-host membrane fusion (Hoffmann et al, 2020). Structural studies have reported the binding of the S protein to the peptidase domain (PD) of ACE2. Under trypsin digestion conditions, cryo-electron microscopy (cryo-EM) revealed that a trimeric S protein could bind two ACE2 PD domains (Yan et al, 2021) (Fig. 4D, PDB ID 7DX8). To explore the potential interaction between CDH1 and ACE2 in this context, we performed molecular docking to predict the binding interface. The resulting model suggested that two CDH1 monomers could bind to the PD of ACE2, and this interaction region partially overlapped with the S protein-ACE2 binding interface (Fig. 4E). This overlap implies potential competition between CDH1 and the S protein for ACE2 binding. The predicted hydrogen bond network involved six residues from CDH1 (Y142, N168, Q197, E54, W59, N20 across two monomers), and six from ACE2 (G319, L320, Q552, K309, Q325, E329) (Fig. 4E). To functionally validate these predicted interaction sites, we generated a panel of CDH1 point mutants including N20A, W59A, Y142A, N168A, Q197A, E54A/W59A, N20A/E54A/W59A (NEW), Y142A/N168A/Q197A (YNQ), and N20A/E54A/W59A/Y142A/N168A/Q197A (NEWYNQ). Co-immunoprecipitation assay showed that mutations W59A, N168A, Q197A, E54A/W59A, NEW, YNQ, and NEWYNQ impaired CDH1-ACE2 binding, whereas N20A and Y142A had no detectable effect (Fig. 4F; Appendix Fig. S8B). These results highlight W59, N168, and Q197 as critical residues for the CDH1-ACE2 binding. Transient expression of three mutants including W59A, N168A, and Q197A in 293T-ACE2 cells significantly enhanced pseudoviral infection compared to wild-type CDH1 (Fig. 4G,H), suggesting that CDH1 binding to ACE2 suppresses viral infection. To determine whether CDH1 competes with the viral S protein for ACE2 binding, we performed competitive pull-down assays. The results demonstrated that CDH1 competed with the S1 subunit of the Spike protein for ACE2 binding in a dose-dependent manner (Fig. 4I; Appendix Fig. S8C). Conversely, the presence of S1 weakened the CDH1–ACE2 interaction (Fig. 4J; Appendix Fig. S8D), further supporting mutual competition between CDH1 and the Spike protein for ACE2 binding. To further confirm the CDH1-ACE2 interaction in an OE-derived system, we employed a proximity ligation assay (PLA) in human olfactory epithelial organoids. The results revealed the physiological proximity between endogenous CDH1 and ACE2, indicating the interaction between two proteins under real cellular system (Fig. 4K). In human OE organoids, the CDH1-ACE2 ligation was found in Krt18^+^ sustentacular cells and Krt14^+^ basal cells (Fig.4L,M). Taken together, these findings validate a direct interaction between CDH1 and ACE2 in both outside and inside the cellular context, and demonstrate that CDH1 competes with the SARS-CoV-2 Spike protein for binding to ACE2, providing a mechanistic basis for its antiviral effect.

### Lower level of CDH1 in SARS-CoV-2-infected bronchial epithelial cells

**Figure 5:**
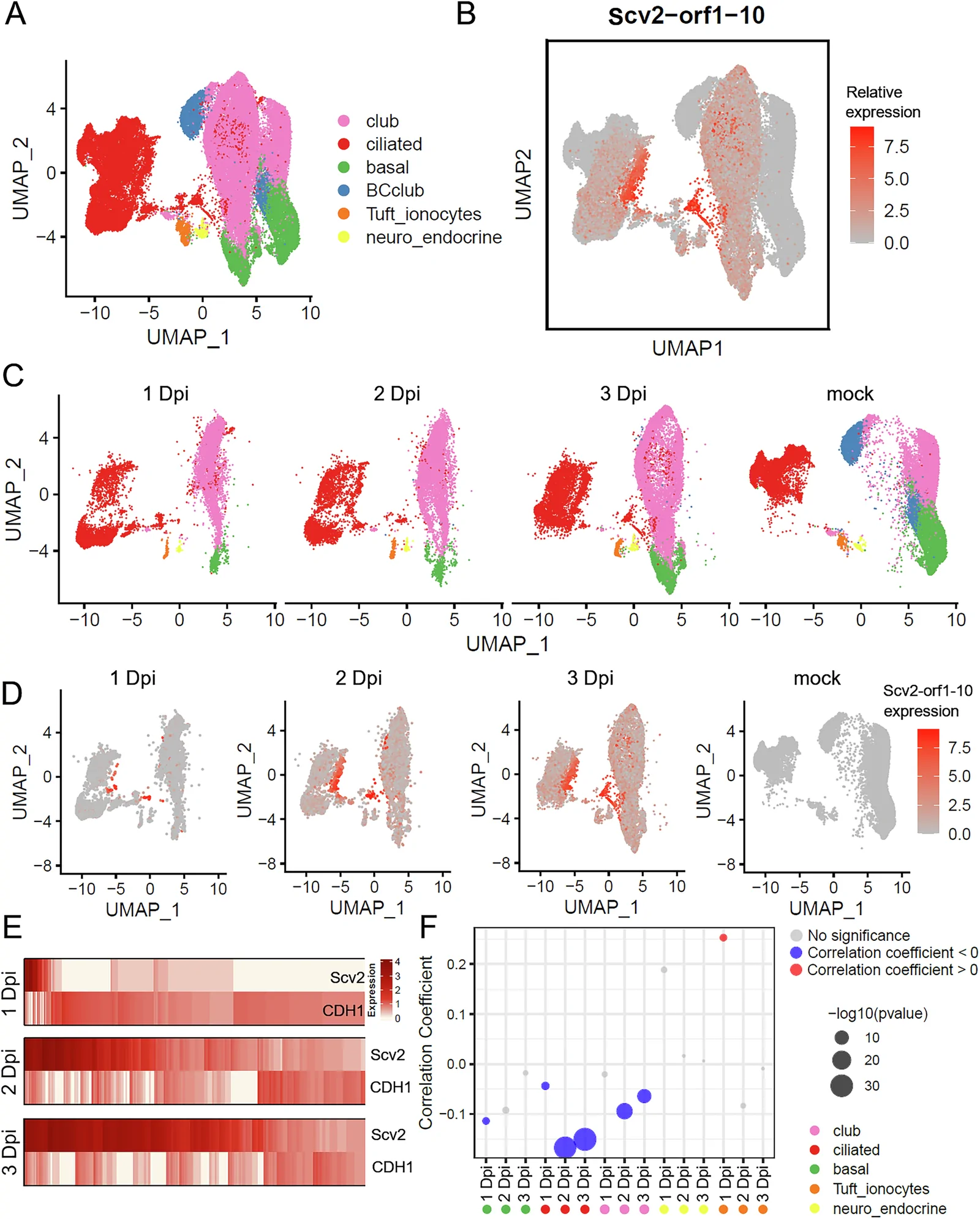
CDH1 expression was negatively correlated with viral load post infection in bronchial epithelial cells. (**A**) UMAP plot showing cell clusters in in-vitro cultured human bronchial epithelial cells (hBECs) at Day 1, 2, and 3 post SARS-CoV-2 infection. This was a combined presentation of (**C**). (**B**) UMAP plot showing infected hBECs expressing SARS-CoV-2 genome Scv2-orf1-10. (**C**, **D**) Separate UMAP plots showing cell clusters (**C**) and expression of Scv2-orf1-10 (**D**) in hBECs at Day 0, 1, 2, and 3 post infection. (**E**) Heatmap showing expression of CDH1 and Scv2-orf1-10 in single ciliated cells at Day 1, 2, and 3 post infection. (**F**) Dot plots showing correlation coefficient between CDH1 and Scv2-orf1-10 in different cell types of hBECs at Day 1, 2, and 3 post infection. The correlations were assessed using Spearman’s rank correlation test.

We then determined whether CDH1 expression correlates with the progression of SARS-CoV-2 infection. Using previously reported scRNA-Seq data [GSE166766 reported in (Ravindra et al, 2021b; Data ref: Ravindra et al, 2021a)], we analyzed in vitro cultured human bronchial epithelial cells (hBECs) infected with SARS-CoV-2 isolate USA-WA1/2020. The infected cells were classified into six distinct subclusters (Fig. 5A). Expression of the SARS-CoV-2 genomic transcript Scv2-orf1-10 was predominantly detected in ciliated cells and club cells (Fig. 5B). As infection progressed, a transitional cell population positioned between basal cells and club cells (BC/club), likely representing basal stem cells undergoing differentiation into club cells, markedly diminished in infected hBECs, whereas this population remained clearly present in uninfected (mock) cells (Fig. 5C). Infected ciliated cells became increasingly enriched at 2 and 3 dpi (Fig. 5D). While Scv2-orf1-10 expression was barely detectable at 1 dpi, it was robustly observed at 2 and 3 dpi, with no expression detected in uninfected cells (Fig. 5D). Notably, in ciliated cells, Scv2-orf1-10 expression exhibited a negative correlation with CDH1 expression, since cells with high viral RNA levels consistently showed reduced CDH1 expression (Fig. 5E). This inverse relationship became more pronounced at 2 and 3 dpi compared to 1 dpi (Fig. 5F). Beyond ciliated cells, a negative correlation between CDH1 expression and viral load was also observed in basal cells at 1 dpi, and club cells at 2 and 3 dpi (Fig. 5F). Collectively, these findings demonstrate that CDH1 expression is inversely associated with viral load in SARS-CoV-2-infected bronchial epithelial cells over the course of infection.

### CDH1 expression is negatively correlated with SARS-CoV-2 infection in lung cells

**Figure 6:**
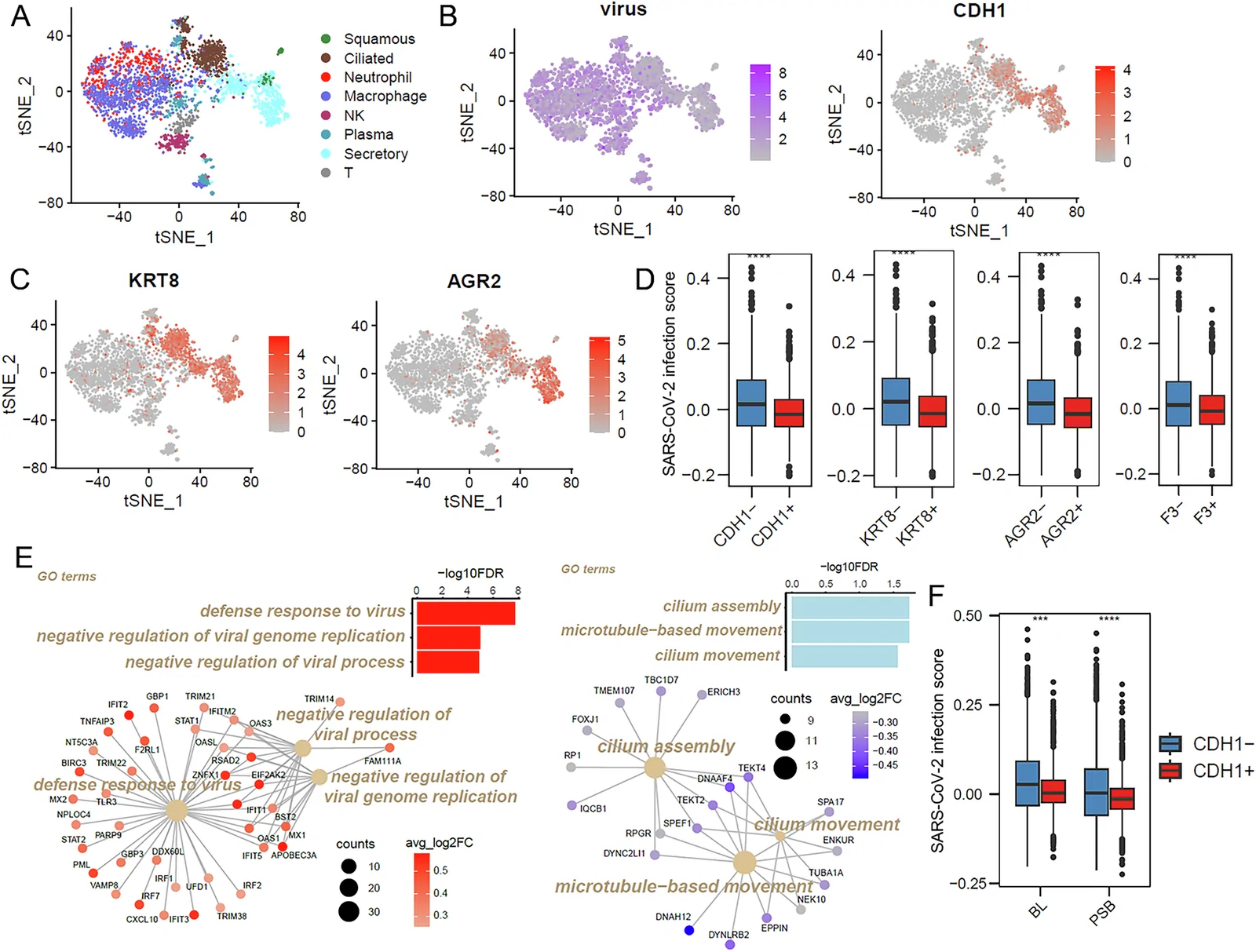
Negative correlation between CDH1 expression and SARS-CoV-2 infection in lung cells. (**A**) tSNE plot showing eight cell subtypes in SARS-CoV-2-infected lung cells. (**B**) tSNE plots showing viral expression and CDH1 expression in infected lung cells. (**C**) tSNE plots showing KRT8 and AGR2 expression in infected lung cells. (**D**) Differential gene set scores of early SARS-CoV-2 infection events between CDH1^+^ and CDH1^−^, KRT8^+^ and KRT8^−^, AGR2^+^ and AGR2^−^, F3^+^ and F3^−^ infected lung cells. CDH1: ****P = 1.15E-13, 2317 CDH1^+^ cells and 768 CDH1^−^ cells, KRT8: ****P = 5.21E-17, 1087 KRT8^+^ cells and 1998 KRT8^−^ cells, AGR2: ****P = 7.87E-15, 799 AGR2^+^ cells and 2286 AGR2^−^ cells, F3: ****P = 0.0000172, 725 F3^+^ cells and 2360 F3^−^ cells. *n* = 8 patients. (**E**) GO analysis of upregulated (red) and downregulated (blue) genes between CDH1^+^ACE2^+^ and CDH1^-^ACE2^+^ infected lung cells. (**F**) Differential gene set scores of early SARS-CoV-2 infection events between CDH1^+^ and CDH1^-^ bronchial lavage (BL) and protected specimen brush (PSB) samples from COVID-19 patients. Significance was determined by unpaired two-samples Wilcoxon rank-sum test. ***P = 0.00024, ****P = 1.49E-22. BL: *n* = 381 CDH1^+^ cells and 7820 CDH1^−^ cells from two patients, PSB: *n* = 6551 CDH1^+^ cells and 3072 CDH1^−^ cells from two patients. For the box plots (**D**, **F**), the centre line represented the median (50th percentile), and the bounds of the box indicated the 25th and 75th percentiles. The whiskers extended to the minima and maxima values within 1.5 × IQR from the box limits. Data points outside this range were plotted individually as outliers.

We next examined the correlation between CDH1 expression and SARS-CoV-2 infection in infected lung cells. Using previously reported scRNA-Seq data from bronchoalveolar lavage fluid (BALF) samples collected from COVID-19 patients infected with the prototypic SARS-CoV-2 lineage [GSE158055 reported in (Ren et al, 2021b; Data ref: Ren et al, 2021b)], infected lung cells were categorized into eight distinct subclusters (Fig. 6A). CDH1 expression was predominantly enriched in lung ciliated and secretory cells, where viral transcripts including ORF1ab and other SARS-CoV-2 genes were relatively lower (Fig. 6B). Other genes associated with viral entry, such as KRT8, AGR2, and F3, which are linked to ACE2, FURIN, TMPRSS2 expression, exhibited a similar negative correlation with viral gene expression as observed for CDH1 (Fig. 6C; Appendix Fig. S9A). However, this correlation was not evident for other ACE2/FURIN/TMPRSS2–related genes, including EPAS1, SIX3, and LYPD2 (Appendix Fig. S9A). To further explore the correlation between SARS-CoV-2 gene expression and CDH1 (or other related genes), we scored CDH1^-^ and CDH1^+^ cells using a human gene set representing early SARS-CoV-2 infection events (GSEA_M45028). Gene set enrichment analysis (GSEA) indicated that the SARS-CoV-2 infection score was significantly lower in CDH1^+^ cells than CDH1^-^ cells. A similar trend was observed in KRT8^+^, AGR2^+^, and F3^+^ cells relative to their negative counterparts (Fig. 6D), but not in cells expressing five other viral entry-related genes (Appendix Fig. S9B). GO analysis indicated that genes upregulated in CDH1^+^ACE2^+^ infected human lung ciliated and secretory cells compared to CDH1^-^ACE2^+^ cells were primarily enriched in biological processes such as negative regulation of viral process, negative regulation of viral genome replication and defense response to virus. In contrast, downregulated genes were mainly associated with cilium assembly and movement (Fig. 6E). To further validate whether CDH1 plays a comparable role in reducing viral susceptibility in lung cells, we analyzed bronchial lavage (BL) and protected specimen brush (PSB) samples from two COVID-19 patients infected with prototypic SARS-CoV-2 lineage (Chua et al, 2020b; Data ref: Chua et al, 2020a). Virus-positive cells were identified from previously reported single-cell RNA sequencing data (Ren et al, 2021b; Data ref: Ren et al, 2021b), revealing viral tropism across seven major cell types (Fig. 6A, excluding plasma cells). Notably, CDH1^+^ cells exhibited significantly reduced viral infection-related gene scores in both BL and PSB samples compared to CDH1^-^ counterparts (Fig. 6F), further supporting an inhibitory role of CDH1 in SARS-CoV-2 infection within lung cells. Collectively, these findings demonstrate that CDH1 expression levels are negatively correlated with SARS-CoV-2 infection in infected lung ciliated and secretory cells.

## Discussion

In this study, we found that CDH1 regulates SARS-CoV-2 infectivity and negatively correlates with viral load in olfactory and bronchial epithelial cells and lung cells. CDH1 interacts with ACE2 and competes with the Spike protein for ACE2 binding. Cells with higher levels of SARS-CoV-2 genome exhibit lower CDH1 expression. Overexpression of CDH1 attenuates pseudoviral and SARS-CoV-2 infection in both heterologous cell and organoid models, whereas CDH1 downregulation enhances infection. These findings suggest that CDH1 serves as a key regulator of SARS-CoV-2 infection in olfactory and other tissues.

The functional role of CDH1 in viral infection was elucidated in this study. First, a HEK-293T cell line stably expressing ACE2 and CDH1 was used to demonstrate that CDH1 overexpression inhibits SARS-CoV-2 infection. However, this heterologous cell line failed to recapitulate the features of olfactory epithelial tissues. To address this limitation, we employed human olfactory mucosa organoids, which contain the major cell types of mucosal tissue (Ren et al, 2021a) and exhibit functional responses to odor stimulation, thereby replicating real tissue at both cellular and physiological levels. In these mucosal organoids, CDH1 showed a similar inhibitory effect on viral infection as observed in the heterologous cell line, supporting the reliability of both models. A limitation of the organoid model is that it does not capture the pseudostratified structure or the inflammatory microenvironment. Further work will aim to incorporate these features into the organoid model to more faithfully replicate native tissue.

The nine shared genes between mouse and human OE, including CDH1, are associated with the SARS-CoV-2 entry genes ACE2, FURIN, and TMPRSS2 and exhibit diverse functions. AQP5 (aquaporin 5) regulates inflammation and cell death (Ren et al, 2017; Wang et al, 2023). SIX3 is a transcriptional factor that governs the developmental trajectory of multipotent progenitor cell (Ferrena et al, 2024) and neuronal differentiation (Shrestha et al, 2023). EPAS1, also known as HIF2α, promotes inflammation and immunity in group 2 innate lymphoid cells (Han et al, 2022) and suppresses anti-inflammatory macrophage mitochondrial metabolism (DeBerge et al, 2021). AGR2 is a mucin chaperone that regulates the unfolded protein response (UPR) (Cloots et al, 2024). VAMP8 mediates mucin exocytosis and maintains tissue homeostasis (Cornick et al, 2019). KRT8 regulates epithelial-mesenchymal transition (Miao et al, 2020) and cell damage (Zhang et al, 2023). Collectively, these genes, which are associated with viral entry genes, potentially function in cell death, cell differentiation, inflammation, cellular stress and damage, further supporting the notion that SARS-CoV-2 infection exerts multiple adverse effects on cellular processes and functions. In the SARS-CoV-2-infected OE region, genes related to ACE2, FURIN, and TMPRSS2 were downregulated compared to uninfected OE (Fig. 2). This suggests a similar trend in the expression changes of these viral entry-related genes as observed for CDH1, potentially reinforcing the important roles of these infection-associated genes in regulating SARS-CoV-2 infectivity.

CDH1 is a critical gene involved in regulating cell adhesion, and its loss initiates tumor progression (Monster et al, 2024). Overexpression of CDH1 is negatively correlated with immune cell infiltration, including that of macrophages and neutrophils (Fan et al, 2022). Given that neutrophil infiltration promotes tumorigenesis, this suggests that CDH1 overexpression exerts an anti-tumor effect (Wang et al, 2014). As an important tight junction gene, reduced expression of CDH1 leads to loss of cellular integrity, which is closely associated with viral infection. A previous study showed that CDH1 expression was significantly lower in SARS-CoV-2-infected Caco-2 cells compared to virus-free cells (Osman et al, 2022), indicating that reduced CDH1 expression facilitates viral infection in intestinal cells. This finding aligns with our observation that CDH1 overexpression inhibits pseudoviral and SARS-CoV-2 infectivity. Further support comes from evidence that CDH1 expression is negatively correlated with SARS-CoV-2 viral load in olfactory epithelial cells, bronchial epithelial cells, and lung ciliated and secretory cells. A recent study identified CDH1 as a significant differentially expressed gene linked to ACE2 in the comorbidity of COVID-19 and asthma (Xu et al, 2024). Additionally, CDH1 is a hub gene enriched in Alzheimer’s disease and COVID-19 (Premkumar and Sajitha Lulu, 2023). KDOAM-25, a KDM5 family demethylase inhibitor, was shown to significantly reduce virus entry into cells, with a concurrent marked increase in CDH1 expression in undifferentiated Caco-2 cells following treatment (Knyazev et al, 2023). Together, these studies support a correlation between CDH1 and SARS-CoV-2 infection. Further investigation into the mechanisms by which CDH1 regulates viral entry into nasal and olfactory epithelial cells may reveal promising targets for antiviral therapy.

It is important to acknowledge that this study was conducted using the prototypic SARS-CoV-2 strain WIV04, one of the earliest isolates of the pandemic. Although this strain serves as a critical reference for understanding the fundamental mechanisms of viral entry and replication, we recognize that the rapidly evolving landscape of SARS-CoV-2 variants of concern (VOCs), such as Delta and Omicron sublineages, harbors mutations in the Spike protein that can substantially alter ACE2 binding affinity. Therefore, caution should be exercised when extrapolating our findings directly to other variants. Future investigations employing pseudotyped viruses or authentic isolates representing currently circulating variants are warranted to determine whether the observed effects of CDH1 on viral infection are conserved across different lineages.

Although our findings establish a negative regulatory role for CDH1 in SARS-CoV-2 entry, a major limitation of this study is the lack of functional entry assays across a broader range of viral species and variants. Future studies employing pseudovirus and live virus systems are necessary to determine whether this effect extends to SARS-CoV-2 variants and other ACE2-dependent coronaviruses (e.g., SARS-CoV-1 and NL63), as well as unrelated specificity controls, thereby confirming the precise specificity of CDH1 in viral entry regulation.

## Methods

**Table:** Reagents and tools table

Reagent/resource	Reference or source	Identifier or catalog number
**Experimental models**		
K18-hACE2 H11 (C57BL/6JGpt)	GemPharmatech Co., Ltd	T037657
HEK293T-ACE2 cell line	Genomeditech Co., Ltd	GM-C12968
HEK293T-ACE2-CDH1 cell line	Fenghui Biotechnology Co. Ltd	FH08531159
**Recombinant DNA**		
pcDNA3.1-mCherry	Shhebio Co., Ltd	Cat #P2418
**Antibodies**		
Human/Mouse/Rat/Hamster ACE‑2 Antibody	R&D Systems	Cat #AF933
Human/Mouse E‑Cadherin Antibody	R&D Systems	Cat #AF748
Purified Mouse Anti-E-Cadherin	BD biosciences	Cat #610182
Cytokeratin 18 Monoclonal Antibody (DC10)	Invitrogen	Cat #MA5-12104
Cytokeratin 18 Polyclonal antibody	Proteintech	Cat #10830-1-AP
Cytokeratin 14 Polyclonal antibody	Proteintech	Cat #10143-1-AP
HRP-conjugated Goat Anti-Human IgG(H + L)	Proteintech	Cat #SA00001-17
mCherry antibody	Proteintech	Cat #68088-1-Ig
Mouse anti-His-Tag mAb	ABclonal	Cat #AE003
Fluorescein (FITC)–conjugated Goat Anti-Rabbit IgG (H + L)	Proteintech	Cat #SA00003-2
Donkey anti-Mouse IgG (H + L) Highly Cross-Adsorbed Secondary Antibody, Alexa Fluor™ 488	Invitrogen	Cat #A-21202
Donkey anti-Rabbit IgG (H + L) Highly Cross-Adsorbed Secondary Antibody, Alexa Fluor™ 488	Invitrogen	Cat #A-21206
Donkey anti-Goat IgG (H + L) Cross-Adsorbed Secondary Antibody, Alexa Fluor™ 488	Invitrogen	Cat #A-11055
Donkey anti-Goat IgG (H + L) Cross-Adsorbed Secondary Antibody, Alexa Fluor™ 647	Invitrogen	Cat #A-21447
HRP-conjugated Goat Anti-Mouse IgG Secondary Antibody	Epizyme	Cat #LF101
HRP-conjugated Goat Anti-Rabbit IgG Secondary Antibody	Epizyme	Cat #LF102
HRP-conjugated Rabbit Anti-Goat IgG (H + L)	Proteintech	Cat #SA00001-4
APC anti-mouse/human CD324 (E-Cadherin)	BioLegend	Cat #147311
APC/Cyanine7 anti-His-Tag	BioLegend	Cat #362633
DAPI	BioLegend	Cat #422801
**Oligonucleotides and other sequence-based reagents**		
QRT-Human-CDH1-FP	Saihengbio	5′-ATTTTTCCCTCGACACCCGAT
QRT-Human-CDH1-RP	Saihengbio	5′-TCCCAGGCGTAGACCAAGA
QRT-Human-GAPDH-FP	Saihengbio	5′-TGGCACCGTCAAGGCTGAGAA
QRT-Human-GAPDH-RP	Saihengbio	5′-TGGTGAAGACGCCAGTGGACTC
QRT-Mouse-CDH1-FP	Saihengbio	5′-CTCCAGTCATAGGGAGCTGTC
QRT-Mouse-CDH1-RP	Saihengbio	5′-TCTTCTGAGACCTGGGTACAC
QRT-Mouse-beta actin-FP	Saihengbio	5′-GGCTGTATTCCCCTCCATCG
QRT-Mouse-beta actin-RP	Saihengbio	5′-CCAGTTGGTAACAATGCCATGT
scAAV.CAG. EGFP.WPREs.U6 vector.forward oligo	Saihengbio	5’-CACATTCTCGAGAATGTGAGCAATTCTGCTTGGTTTTTTGGTACCCGCGTCGACATTG
scAAV.CAG. EGFP.WPREs.U6 vector.reverse oligo	Saihengbio	5’-GCTCACATTCTCGAGAATGTGAGCAATTCTGCTTGGcGGTGTTTCGTCCTTTCCACAA
QRT-SARS-CoV-2 Np-qF1	Saihengbio	5’-GAGCTACCAGACGAATTCGTGGT
QRT-SARS-CoV-2 Np-qR1	Saihengbio	5’-CTCAAGTGTCTGTGGATCACG
CDH1-N20A-F	Saihengbio	5’-CCCATTTCCTAAAGCCCTGGTTCAGATCAAATCCAACAAAG
CDH1-N20A-R	Saihengbio	5’-GATCTGAACCAGGGCTTTAGGAAATGGGCCTTTTTC
CDH1-E54A/W59A-F	Saihengbio	5’-GAAACAGGAGCGCTGAAGGTGACAGAGC
CDH1-E54A/W59A-R	Saihengbio	5’-CTTCAGCGCTCCTGTTTCTCTTGCAATAATAAAGACA
CDH1-Y142A-F	Saihengbio	5’-TGAACACCGCCAATGCCGCCATC
CDH1-Y142A-R	Saihengbio	5’-GGCATTGGCGGTGTTCACATCATCG
CDH1-N168A-F	Saihengbio	5’-CATTAACAGGGCCACAGGAGTCATCAGT
CDH1-N168A-R	Saihengbio	5’-CTCCTGTGGCCCTGTTAATGGTGAAC
CDH1-Q197A-F	Saihengbio	5’-CTGACCTTGCAGGTGAGGGGTTAAG
CDH1-Q197A-R	Saihengbio	5’-CCTCACCTGCAAGGTCAGCAGCTTG
**Chemicals, enzymes and other reagents**		
Recombinant human ACE2-hFC (Uniprot ID: Q9BYF1, 18–740 aa)	Mabnus	Cat #GS13193
Recombinant human CDH1 (Uniprot lD: P12830, 155–707 aa)	Mabnus	Cat #GS10128
Recombinant Human FC Protein	Mabnus	Cat #GS15091
Biotinylated Human ACE2/ ACEH Protein, Fc, Avitag	ACRO Biosystems	Cat #AC2-H82F9
Human E-Cadherin/ Cadherin-1 (155-709) Protein, Fc Tag	ACRO Biosystems	Cat #ECD-H5256
SARS-CoV GD01 Spike Trimer Protein (R667A, KV968-969PP), His Tag (MALS verified)	ACRO Biosystems	Cat #SPN-S52Ht
SARS-CoV-2 (COVID-19) s1 protein, His Tag (MALS verified)	ACRO Biosystems	Cat #S1N-C52H3
HCoV-NL63 S1 protein, His Tag	ACRO Biosystems	Cat #SIN-V52H3
EZ ECL pico chemiluminescent fluid	Shanghai Life-iLab Biotech	Cat #AP34L024
Protein A/G agarose	Epizyme Biotech	Cat #YJ201
anti-mCherry magnetic beads	FITGENE	Cat #FI8206
Streptavidin magnetic beads	TargetMol	Cat #C0090
Coomassie Brilliant Blue	Epizyme	Cat #PS111
Duolink ® In Situ PLA ® Probe Anti-Mouse PLUS	Merck	Cat #DUO92001-30RXN
Duolink ® In Situ PLA ® Probe Anti-Goat MINUS	Merck	Cat #DUO92006-30RXN
Duolink® In Situ Detection Reagents Red	Merck	Cat #DUO92008-30RXN
**Software**		
Seurat (version 3.2.3)	https://satijalab.org/seurat/	
R (version 4.1.0)	https://www.r-project.org/	
SeuratDisk (version 0.0.0.9021)	https://github.com/mojaveazure/seurat-disk	
stats (version 3.6.2)	https://www.r-project.org/	
STRING (version 12.0, https://cn.string-db.org/)	https://cn.string-db.org/	
Cytoscape (version 3.8.2)	http://www.cytoscape.org	
GOSemSim (version 2.24.0)	https://bioconductor.org/packages/GOSemSim/	
ggplot2 (version 3.5.1)	https://ggplot2.tidyverse.org/	
clusterProfiler (version 3.15)	https://bioconductor.org/packages/clusterProfiler/	
enrichplot package (version 1.26.5)	https://bioconductor.org/packages/enrichplot/	
ComplexHeatmap (version 2.22.0)	https://bioconductor.org/packages/ComplexHeatmap/	
DESeq2 (version 1.47.1)	https://bioconductor.org/packages/DESeq2/	
ZDOCK server for analysis and verification (Pierce, Wiehe et al, 2014)	https://zdock.umassmed.edu/ Pierce et al, 2014	
PyMOL (DeLano Scientific LLC, San Carlos, CA, USA)	https://pymol.org/	
ASTRA software (Wyatt Technology)	https://www.wyatt.com/products/software/astra.html	
ggpubr R package (Version 0.6.0)	https://rpkgs.datanovia.com/ggpubr/	
ImageJ software (version 1.53 K).	https://imagej.nih.gov/ij/	
GraphPad Prism v10.4.0	https://www.graphpad.com/	
FlowJo v10.9	https://flowjo.com/	
LAS AF Lite software	https://www.leica-microsystems.com/products/microscope-software	
**Other**		
chemiluminescence detection system	Tanon	4600SF
LEICA TCS SP8 confocal microscope	Leica	SP8

### Animals

Krt18-hACE2 mice were purchased from GemPharmatech Corp. (Nanjing, China). Mice were housed at the Experimental Animal Facility of Shanghai Medical College, Fudan University (Shanghai, China), with ad libitum access to food and water under a 12-h light/dark cycle. Only male mice were used in this study.

### scRNA-seq data analysis

Four human olfactory epithelium scRNA-seq datasets (GSM4142870, GSM4142871, GSM4142872, GSM4142873) were downloaded from Gene Expression Omnibus (GEO) under accession code GSE139522 (Durante et al, 2020b; Data ref: Durante et al, 2020a). Four mouse olfactory epithelium scRNA-seq datasets (GSM4363336, GSM4363337, GSM4363338, GSM4363339) were downloaded from GEO under accession code GSE146043 (Tan et al, 2020b; Data ref: Tan et al, 2020a). Datasets were quality-controlled, filtered and integrated using the Seurat (version 3.2.3) standard integration workflow as described (Durante et al, 2020b; Tan et al, 2020). Cell types were identified based on reported cell type-specific markers (Durante et al, 2020b; Tan et al, 2020). SARS-CoV-2-infected human bronchial epithelial cells (hBECs) data were downloaded from GEO under accession code GSE166766(Ravindra et al, 2021b; Data ref: Ravindra et al, 2021a). The feature-barcode matrices were read into R (version 4.1.0), excluding cells expressing fewer than 200 genes and genes expressed in fewer than three cells. Cells with more than 10% of reads mapping to mitochondrial genes were filtered out. Cell clustering was performed using the “FindNeighbors” and “FindClusters” functions using the first 40 principal components (PCs) and a resolution of 0.3. To infer ACE2-positive cells, we utilized the “WhichCells” function in Seurat (version 3.2.3) with the expression parameter “ACE2 > 0”. Infected single-cell h5ad data with annotated cell types were downloaded from http://covid19.cancer-pku.cn. The h5ad data were converted into a Seurat object using the “LoadH5Seurat” function in SeuratDisk (version 0.0.0.9021). To assess the correlation between target gene expression and viral expression, scatter plot analysis was performed in cells double-positive for the target gene and ACE2 within the ciliated and secretory cell cluster using the “FeatureScatter” function in Seurat (version 3.2.3). Visualizations were generated using the DotPlot and DimPlot functions within Seurat (version 3.2.3).

### Identification of correlated genes and network construction

To identify genes with high expression correlation with ACE2, TMPRSS2 and FURIN using human and mouse OE scRNA-seq data, we employed the “cor.test” function from stats package (version 3.6.2). For mouse OE, normalized expression counts of all genes were used to calculate correlations with mouse Ace2 using Spearman’s rank correlation. Genes with *p*-values < 0.05 were considered significantly correlated. The genes correlated with ACE2, TMPRSS2, and FURIN were overlapped to identify common genes in mouse OE. The same approach was applied to human OE scRNA-seq data. STRING network analysis was performed using STRING (version 12.0, https://cn.string-db.org/) and visualized with Cytoscape (version 3.8.2).

### Hub-gene analysis

We used GOSemSim (version 2.24.0) to identify hub genes interacting with all other genes within the gene set. The “mgeneSim” function was used to calculate semantic similarity score for genes at the biological process (BP), cellular component (CC) and molecular function (MF) levels. The Wang method was used for semantic similarity measurement, and the best-match average (BMA) method was used for combining multiple terms. The average score for each gene across BP, CC, and MF levels was calculated to obtain a final score. LYPD2 was excluded from the analysis as it lacked a score at the biological process level. Visualization was performed using ggplot2 (version 3.5.1).

### Gene ontology (GO) analysis

The “FindMarkers” function in Seurat was used to identify differentially expressed genes between CDH1-positive and CDH1-negative cells within infected ciliated and secretory cell clusters, using default parameters. GO analysis was performed using the “enrichGO” function in clusterProfiler (version 3.15), with a qvalue cutoff of 0.05. The top GO terms were selected and visualized with ggplot2 (version 3.5.1). The results were further visualized as a network diagram using the “Cnetplot” function from the enrichplot package (version 1.26.5).

### Expression correlation analysis

To assess the correlation between CDH1 expression and Scv2-orf1-10 expression in hBEC scRNA-seq data, normalized expression counts of CDH1 and Scv2-orf1-10 across each cell type and day post infection were used for analysis. Correlations were calculated using the “cor.test” function with Spearman’s method. Correlation coefficients and *P* values were visualized using ggplot2. Positive correlations were shown in red, negative correlations in blue, and correlations with *P* values greater than 0.05 were shown in gray. Gene expression heatmaps were generated using the “Heatmap” function in ComplexHeatmap (version 2.22.0).

### Gene set score analysis

To score individual cells for SARS-CoV-2 infection activity in virus-positive scRNA-seq data, we used the “AddModuleScore” function from Seurat (version 3.2.3). An early SARS-CoV-2 infection gene set (M45028) was downloaded from the MsigDB database and used to score each cell. Cells positive for a specific gene were defined as those with a count value greater than 0 for that gene. For lower airways data analysis, RDS data were downloaded from https://doi.org/10.6084/m9.figshare.12436517.v2 (Chua et al, 2020b; Data ref: Chua et al, 2020a). Target cells were extracted based on the defined cell types for scoring. Expression of early SARS-CoV-2 infection genes was evaluated in CDH1-positive and CDH1-negative cells across patient samples from two sources, including bronchial lavage (BL) and protected specimen brushes (PSB).

### GeoMx DSP data analysis

GeoMx DSP data (GSE176080) was downloaded from GEO (Khan et al, 2021b; Data ref: Khan et al, 2021a). The 17 areas of interest (AOIs) were divided into ORF1ab-low (*n* = 7) and ORF1ab-high (*n* = 10) groups according to the original descriptions (Khan et al, 2021). The geometric mean of the log2-normalized ORF1ab expression (9.92) was used as the threshold to define ORF1ab-low and ORF1ab-high AOIs. To analyze the correlation, CDH1 and ORF1ab expression levels were extracted for each AOI. Spearman correlation between CDH1 and ORF1ab was calculated using the “cor.test” function. A bar plot showing CDH1 expression levels in ORF1ab-low and ORF1ab-high AOIs was generated using ggplot2 (version 3.5.1). Differentially expressed genes between ORF1ab-low and ORF1ab-high AOIs were identified using the edgeR (version 4.4.2) via the likelihood ratio test based on raw counts downloaded from GEO (GSE176080) (Khan et al, 2021b). The volcano plot was generated using ggplot2 (version 3.5.1) and ggVolcano (version 0.0.2).

### Dissection and preparation of human OE

Biopsies of the superior nasal turbinate were obtained from a 34-year-old male patient (for frozen sections of OE) by surgeons at the Eye & ENT Hospital, Fudan University. Tissue samples were transferred into containers with 4% paraformaldehyde (PFA, #V900894, Sigma-Aldrich) and fixed for 12 h. Samples were then dehydrated in a gradient of 10%, 20%, and 30% sucrose, followed by embedding in O.C.T. compound. The frozen tissues were cut into 20 μm coronal sections using a Leica CM1950 cryostat and collected onto Superfrost Plus adhesion microscope slides. Slides were air-dried at room temperature, and slide boxes were sealed prior to storage at −80 °C.

### Immunohistochemistry for mouse/human OE sections and human OE organoids

K18-hACE2-H11 mice were purchased from GemPharmatech Co. Ltd. This strain is an ACE2 knock-in mouse on the C57BL/6JGpt background, with human ACE2 overexpressed at the H11 safe harbor under the control of the human cytokeratin 18 promoter. For immunohistochemistry, mice were deeply anesthetized by intraperitoneal injection of ketamine-xylazine (200 and 15 mg/kg body weight) and transcardially perfused with PBS (#7674–14-5, Sinopharm Chemical) followed by 4% PFA. Heads were fixed in 4% PFA overnight at 4 °C, decalcified in 0.5 M EDTA (#15575020, Invitrogen), and infiltrated with a series of sucrose solutions before embedding in O.C.T. compound. Frozen tissues were cut into 20 μm coronal sections on a cryostat. After rinsing with PBS, the mouse or human tissue sections were blocked for 60 min in PBS containing 0.3% Triton X-100 and 5% bovine serum albumin (BSA), followed by incubation with the primary antibodies at 4 °C overnight. Primary antibodies were diluted in blocking solution, and included goat anti-ACE2 (1:200, AF933, R&D Systems), mouse anti-CK18 (1:100, MA5-12104, Invitrogen), and goat anti-CDH1 (1:300, AF748, R&D Systems). After three washes in PBST (0.3% Triton X-100 in PBS), sections were incubated with secondary antibodies for one hour at room temperature, including Alexa Fluor 488 donkey anti-rabbit (A-21206, Thermo Fisher Scientific) and Alexa Fluor 488 donkey anti-goat (A-11055, Thermo Fisher Scientific). Nuclei were counterstained with DAPI (Thermo Fisher Scientific). Tissues were mounted in Vectashield (Vector Laboratories). Images were acquired using a Leica SP8 confocal microscope with LAS AF Lite software.

For immunostaining of cultured organoids, organoids were collected by centrifugation, washed with PBS, fixed in 4% PFA for 20 min, and then dehydrated in 30% sucrose at 4 °C overnight. Organoids were embedded in warm gelatin/sucrose solution, equilibrated at 37 °C for 15 min, and then solidified at −20 °C. The solidified organoid-gelatin/sucrose complex was embedded in O.C.T. compound, and 20-μm-thick sections were prepared using a Cryostat (CM1950, Leica). Sections were washed with PBS and incubated with blocking buffer containing SuperBlock (Thermo Fisher Scientific), 2% (v/v) donkey serum, and 0.3% Triton X-100 at room temperature for 1 h. Primary antibody incubation, including goat anti-CDH1 and in-house generated anti-SARSr-CoV Rp3 N antibody (1:1000) (Zhou et al, 2020), was performed overnight at 4 °C. After washing with PBS, appropriate secondary antibodies were used for visualization. Images were acquired using a Leica SP8 confocal microscope with LAS AF Lite software.

### Transient CDH1 overexpression in HEK293T-ACE2 cell line

The HEK293T-ACE2 cell line was purchased from Genomeditech (Shanghai, China). Cells were cultured in DMEM (Gibco) supplemented with 10% fetal bovine serum (FBS) (Gibco), 100 U/mL penicillin–streptomycin solution (Gibco) and 1 µg/mL puromycin (Sigma-Aldrich). Cells were maintained at 37 °C in a humidified incubator with 5% CO_2_. The cell line used in this study were authenticated by STR profiling and were confirmed to be negative for mycoplasma contamination before use.

Four human CDH1 transcript variants were synthesized by GenScript (Nanjing, China). Each transcript was inserted into the pcDNA3.1-mCherry plasmid (Addgene, 128744). HEK293T-ACE2 cells were seeded at a density of 10,000 cells per well in 96-well culture plates with 200 μL of medium per well and cultured overnight. For transfection, cells in each well were incubated with 100 ng of plasmid and 0.2 μL of Lipofectamine 2000 (Invitrogen) in 200 μL DMEM supplemented with 10% FBS, and medium was replaced after 6 h. Pseudoviral infection was performed at 24 h post transfection.

### Immunostaining for HEK293T-ACE2 cells

HEK293T-ACE2 cells were seeded onto poly-L-lysine-coated coverslips in 24-well plates at a density of 2.5 × 10^4^ cells per well. Following an overnight incubation, the cells were transiently transfected with plasmids encoding four distinct CDH1 variants using Lipofectamine 2000 (Invitrogen) according to the manufacturer’s instructions. At 48 h post-transfection, the cells were subjected to immunofluorescence staining. Briefly, cells were fixed with 4% PFA for 30 min at room temperature. After washing with PBS, the cells were blocked with BSAT buffer (5% BSA and 0.3% Triton X-100 in PBS) for 1 h at room temperature. Subsequently, the cells were incubated overnight at 4 °C with primary antibodies including anti-CDH1 (1:200, 610182, BD) and anti-ACE2 (1:200, AF933, R&D Systems). After PBST washing, the coverslips were incubated with secondary antibodies including Alexa Fluor 488 Donkey anti-Mouse (1:300, A-21202, Invitrogen) and Alexa Fluor 633 Donkey anti-Goat (1:300, A-21082, Invitrogen) for 1 h at room temperature in the dark. Finally, nuclei were counterstained with DAPI, and the coverslips were mounted in Vectashield (Vector Laboratories). Images were taken under a SP8/Leica confocal microscope with LAS AF Lite software.

### Establishment of HEK293T-ACE2 cell line stably expressing CDH1 variant 1

The HEK293T-ACE2-CDH1 cell line was established by Fenghui Biotechnology Co. Ltd. (Hunan, China). The full-length of human CDH1 variant 1 gene was cloned into the pLV-CMV-MCS-EF1-mCherry-T2A-Hygro vector. HEK293T cells were transfected with hCDH1-v1construct to produce lentivirus with a titer of 1 × 10^8^ TU/mL. HEK293T-hACE2 cells seeded in a six-well plate at 70% confluence were then infected with lentivirus encoding hCDH1-v1. The medium was replaced with fresh medium at 48 h post infection. Hygromycin B (Sigma-Aldrich) was added at a final concentration of 50 µg/mL for selection. The selection medium was replaced every 3 days until the proportion of fluorescent cells exceeded 90%. Cells were then maintained in DMEM supplemented with 10% FBS, 100 U/mL penicillin–streptomycin, 1 µg/mL puromycin, and 50 µg/mL hygromycin B (Sigma-Aldrich).

### Pseudoviral infection

SARS-CoV-2 Spike protein pseudotyped virus with GFP-T2A-Luciferase (GM21318LV, 5 × 10^7^ TU/mL) was purchased from Genomeditech (Shanghai, China). For pseudoviral infection in cells transiently or stably expressing CDH1, the culture medium was replaced with 120 μL DMEM containing pseudovirus. Cells were infected with pseudovirus at multiplicities of infection (MOI) of 0.2 and 0.005. After 6 h of incubation, the pseudovirus-containing medium was removed. GFP fluorescence in infected cells was captured using a Leica DMi8 microscope at 24, 48 and 72 h post infection.

### Molecular docking

The protein interaction network of ACE2 and CDH1 was analyzed using molecular docking with AutoDock Vina software. The structure model of human ACE2 (PDB code: 7DX8) was used as the docking template. The human CDH1 molecular model was extracted from the complex structure (PDB code: 7STZ) as the docking template. Crystallized water molecules and metal ions present in the structures were removed. Nonpolar hydrogen atoms were merged into both the ligands and the target by AutoDock Tools. The same molecular docking process was also validated using the ZDOCK server (Pierce et al, 2014). Ten conformations were generated, and the conformation corresponding to the best interaction between ACE2 and CDH1 was selected for subsequent evaluation. Structural models were visualized and analyzed using PyMOL (DeLano Scientific LLC, San Carlos, CA, USA).

### Co-immunoprecipitation assay and western blot

Recombinant human ACE2-hFC (Uniprot ID: Q9BYF1, 18–740 aa) and recombinant human CDH1 (Uniprot lD: P12830, 155–707 aa) lyophilized protein powders were purchased from Mabnus Biological Technology Incorporation (China). To determine the interaction between CDH1 and ACE2, co-immunoprecipitation (co-IP) assays were performed. 40 µg of ACE2 and CDH1 proteins were dissolved in sterile PBS containing 6% trehalose. In total, 25 μL of protein A/G agarose (Epizyme Biotech, #iYJ201) was added to the protein solution and incubated overnight at 4 °C with gentle agitation. The supernatant was discarded, and the remaining agarose was rinsed with PBS, followed by addition of 100 μL of 1×SDS-PAGE loading buffer. After heating at 100 °C for 10 min, the supernatant was collected for SDS-PAGE separation. Proteins were transferred to nitrocellulose membranes (Millipore, #HATF00010), and the membranes were incubated with antibodies against hFC (1:2000, Proteintech, SA00001-17) and CDH1 (R&D, #AF748). Antibody binding was detected using EZ ECL pico chemiluminescent substrate (Shanghai Life-iLab Biotech, #AP34L024) with a chemiluminescence detection system (Tanon, 4600SF).

### Multi-angle light scattering

SEC-MALS experiments were performed using the Dynapro plate reader Ⅲ system coupled to a DAWN HELEOS Ⅱ multi-angle light scattering instrument and an Optilab differential refractometer (Wyatt Technology). For chromatographic separation, a SRT SEC-300 size exclusion column (Sepax Technologies) was used with a 100 μL sample containing 100 μg of CDH1 and 100 μg of ACE2, at a flow rate of 0.5 mL/min in running buffer (PBS, 6% Trehalose, pH 7.4, sterilized through 0.2 μm filter). The outputs were analyzed using ASTRA software (Wyatt Technology), and the molecular weight (Mw) of the complexes was calculated.

### SARS-CoV-2 infection

All experiments with wild-type SARS-CoV-2 (#IVCAS 6.7512) with a titer of 6.31 × 10^5^ TCID_50_/mL were performed in the BSL-3 facilities at the Wuhan Institute of Virology, following procedures and protocols thoroughly reviewed and approved by the Biosafety Committee of the institute. The complete genome sequence of this SARS-CoV-2 isolate (WIV04) is available under GenBank accession number MN996528.1. The virus dilution was prepared by adding 100 μL of virus stock to 900 μL of DMEM, and 95 μL of this dilution was then added to 4 mL of DMEM to generate virus working solution 1. Virus working solution 2 was obtained by further diluting the virus working solution 1 by a factor of 10. For SARS-CoV-2 infection, cells were seeded in 48-well plates (1.5 × 10^5^ cells per well) and infected the following day for 1 h with 200 μL of virus working solution 1 (MOI of 0.002) or 200 μL of virus working solution 2 (MOI of 0.0002). Cells were then rinsed three times with DMEM, and then the culture medium was replaced with DMEM supplemented with 2% FBS. At 1- and 2-day post infection (dpi), cells were fixed with 4% PFA for 24 h at 4 °C. Fluorescence of SARS-CoV-2-infected cells was captured using an AMF4300 Evos FL Cell Imaging System (Life Technologies, Carlsbad, CA, USA).

For SARS-CoV-2 infection, organoids at day 5 post AAV infection were extracted from Matrigel using a cell recovery solution (Corning, 354253) at 4 °C for 1 h. Organoids were then infected with virus at an MOI of 1 and incubated at 37 °C for 3 h in a 5% CO_2_ incubator. Following infection, the organoids were washed twice with PBS and resuspended in Matrigel. The resuspended organoids were then placed in a CO_2_ incubator at 37 °C.

### Immunofluorescence assay on heterologous cell lines

Heterologous cell susceptibility was assessed by immunofluorescence assay targeting Rp3 Np in virus-incubated cells. Briefly, at 24 or 48 h post infection (hpi), cells were fixed with 4% PFA at room temperature for 30 min and permeabilized with 0.1% Triton X-100. Cells were blocked with 5% BSA in PBS for 1 h and then incubated with a rabbit anti-Rp3 Np polyclonal antibody for 1 h. This rabbit polyclonal antibody against SARSr-CoV Rp3 Np, which shares >90% amino acid identity with SARS-CoV-2, was generated in-house. After three washes with PBS, cells were incubated with Fluorescein (FITC)-conjugated goat anti-rabbit IgG (H + L) for 1 h, followed by DAPI staining for 15 min. After three washes with PBS, fluorescence images were acquired and examined using an AMF4300 Evos FL cell imaging system (Life Technologies, Carlsbad, CA, USA).

### Native polyacrylamide gel electrophoresis and In-gel digestion

Proteins were incubated under various buffer conditions at room temperature for 30 min. After incubation, samples were mixed with 5× native non-reducing loading buffer (final concentration 1×; P0016N, Beyotime). Protein mixtures were then loaded onto a HEPES-based native precast gel (C601104-0001, Sangon Biotech) alongside a native protein marker (C630010, Sangon Biotech). Electrophoresis was performed on ice at a constant voltage of 120 V for 60 min. Following electrophoresis, the gel was stained with Coomassie Brilliant Blue (PS111, Epizyme).

The gel piece corresponding to the molecular mass of the target protein was excised and destained with 50% acetonitrile in 50 mM ammonium bicarbonate (NH_4_HCO_3_). The destained gel piece was dehydrated with 100% acetonitrile for 5 min and then incubated with 10 mM TCEP and 40 mM CAA at 60 °C for 30 min. The gel piece was again dehydrated with 100% acetonitrile. Finally, the gel piece was rehydrated and digested with 2 μg of trypsin in 50 mM NH_4_HCO_3_ at 37 °C overnight for in-gel digestion. After digestion, peptides were extracted from the gel piece with 50% acetonitrile/0.1% formic acid. The extracted peptides were dried in SpeedVac concentrator and resuspended in 0.1% formic acid for LC-MS/MS analysis.

### LC-MS/MS analysis and data processing

The peptide samples were dissolved in mobile phase A (0.1% formic acid) and separated using an EASY nLC-1200 system (Thermo Scientific). The nano-liquid chromatography gradient was maintained at a constant flow rate of 400 nL/min and consisted of the following steps: an increase from 2% to 7% mobile phase B (0.1% formic acid in 80% acetonitrile) over 1 min, from 7 to 35% over 35 min, from 35 to 55% over 9 min, ramping to 100% over 7 min, and held at 100% for the final 8 min. The separated peptides were subjected to nano-electrospray source followed by analysis on a Q Exactive HF-X mass spectrometer. The electrospray voltage was set to 2.0 kV, and intact peptides were detected in the Orbitrap at a resolution of 60,000. Peptides were then selected for MS/MS using a normalized collision energy (NCE) setting of 27, and fragments were detected in the Orbitrap at a resolution of 15,000. Mass Spectra were acquired in data-dependent scan mode, which included selection of the 20 most abundant precursor ions from each MS spectrum for MS/MS analysis with a dynamic exclusion time of 20 s.

Mass spectra were processed and searched using Proteome Discoverer (version 2.4, Thermo Scientific) against the human SwissProt protein database (release 2023_09). Trypsin/P (or other enzymes, as applicable) was specified as the cleavage enzyme, allowing up to two missed cleavages. The mass tolerance for precursor ions was set to 10 ppm, while the mass tolerance for fragment ions was set to 0.02 Da. Carbamidomethylation on cysteine was specified as a fixed modification, while oxidation on methionine and acetylation on protein N-termini were specified as variable modifications. Peptide confidence was set to high.

### Site-directed mutagenesis of CDH1 and co-immunoprecipitation assay

Pairs of complementary oligonucleotide primers were designed for each desired point mutation (N20A, W59A, E54A, Y142A, N168A, Q197A), with each primer pair incorporating the corresponding alanine substitution codon. CDH1 mutants were generated using a loop-PCR-based site-directed mutagenesis strategy, with the parental plasmid pcDNA3.1-CDH1-mCherry serving as the template. PCR amplification was performed in a 50 μL reaction volume at an annealing temperature of 58 °C and an extension time of 4 min. Following PCR, 1 μL of DpnI enzyme (Thermo Scientific, ER1705) was added directly to the reaction mixture, gently mixed, and incubated overnight at 37 °C. After DpnI digestion, the mixture was used directly to transform DH5α competent cells. Positive clones were selected and subjected to DNA sequencing. Sequential rounds of the mutagenesis protocol were performed to introduce additional mutations into existing single mutant plasmids.

Wild-type CDH1 and CDH1 mutants cloned into the pcDNA3.1-mCherry vector were expressed in HEK293T cells. At 48 h post transfection, total cellular proteins were lysed in immunoprecipitation (IP) buffer containing 25 mM Tris-HCl (pH 7.4), 150 mM NaCl, 1 mM EDTA, and 1% Triton X-100, supplemented with freshly prepared protease inhibitor cocktail (HY-K0010, MCE) and 100 μM PMSF (HY-B0496, MCE). The lysates were then incubated with recombinant ACE2 protein (C-terminal hFc-tagged, amino acids 18-740, GS13193, Mabnus). The CDH1-ACE2 complex was immunoprecipitated using anti-mCherry magnetic beads (FI8206, FITGENE) overnight at 4 °C. The precipitated complexes were washed five times with IP buffer, eluted with SDS loading buffer (LT101, Epizyme), and separated by SDS-PAGE, followed by immunoblotting using antibodies against ACE2 (AF933, R&D Systems) and mCherry (68088-1-Ig, Proteintech).

### Pull-down assay

ACE2 recombinant protein was immobilized on Streptavidin Magnetic Beads by incubating 300 ng of ACE2-Avi tag (18–740 aa, AC2-H82F9, Acro) with Streptavidin Magnetic Beads (C0090, TargetMol) in 500 µL of IP buffer. The bead–protein complex was then incubated with prey proteins, including 300 ng of Spike protein subunit 1 with a C-terminal His tag (S1-His; 16–685 aa, S1N-C52H3, Acro) together with increasing amounts (0, 300, 1500, or 3000 ng) of human recombinant CDH1 protein (155–709 aa, ECD-H5256, Acro). Alternatively, 2000 ng of CDH1 was incubated with graded concentrations of Spike protein S1-His (0, 300, 600, or 1200 ng). Samples were gently rotated at 4 °C overnight. Proteins bound to the beads were eluted with SDS loading buffer (LT101, Epizyme), separated by SDS-PAGE, and analyzed by Western blot using antibodies against ACE2, CDH1, and His (AE003, Abclonal).

### OE organoid culture

OE organoids were cultured using previously reported protocol with some modifications (Ren et al, 2021a). Human OE tissues for organoid culture were derived from 33-, 56-, and 66-year-old male patients. Krt18-hACE2 mice and human olfactory epithelium tissues were collected and rinsed twice with ice-cold Dulbecco’s Phosphate-Buffered Saline (DPBS). The tissues were then minced on ice in a 15 mL centrifuge tube containing 5 mL of 0.25% Trypsin-EDTA, followed by enzymatic digestion in a cell culture incubator (37 °C, 5% CO_2_) for 30 min. Digestion was terminated by adding a pre-prepared stop solution consisting of DMEM/F12 supplemented with GlutaMAX (10565-018, Gibco), 10% FBS, 10 mM HEPES (15630-080, Gibco), and 1% Penicillin–Streptomycin (15140-122, Gibco). The resulting cell suspension was passed through a 40 μm cell strainer (352340, Falcon), centrifuged at 300×*g* for 5 min, and the supernatant was removed. The cell pellet was then resuspended in growth factor-reduced Matrigel (354230, Corning), and 40 μL droplets of the cell–Matrigel mixture were plated into a 24-well culture plate. The plate was incubated at 37 °C with 5% CO_2_ for 30 min to allow Matrigel polymerization. Subsequently, 500 μL of organoid culture medium was gently added to each well. The medium was replaced every 2 days.

### Quantitative PCR

Six days after SARS-CoV-2 infection, total RNA was extracted from OE organoids using the TRIzol Reagent (catalog #15596026, Invitrogen) following the manufacturer’s instructions. The extracted RNA was immediately dissolved in Nuclease-Free Water (catalog #AM9938, Invitrogen), and its purity and concentration were assessed using a NanoDrop 2000 spectrophotometer (Thermo Scientific). First-strand cDNA was synthesized from total RNA using the PrimeScript RT Master Mix (catalog #RR036A, Takara). Quantitative real-time PCR was conducted using an Analytik Jena real-time PCR system. Each reaction contained cDNA template, 0.2 mM of each primer, ChamQ Blue Universal SYBR qPCR Master Mix (catalog #Q312-02, Vazyme), and nuclease-free water. The thermal cycling conditions were as follows: initial denaturation at 95 °C for 1 min, followed by 40 cycles of 95 °C for 20 s, 60 °C for 20 s, and 72 °C for 30 s. Relative gene expression levels were calculated using the 2^ΔΔCt^ method. Primer sequences were as follows: Human-CDH1: forward 5′-ATTTTTCCCTCGACACCCGAT, reverse 5′-TCCCAGGCGTAGACCAAGA; Human-GAPDH: forward 5′-TGGCACCGTCAAGGCTGAGAA, reverse 5′-TGGTGAAGACGCCAGTGGACTC; Mouse-CDH1: forward 5′-CTCCAGTCATAGGGAGCTGTC, reverse 5′-TCTTCTGAGACCTGGGTACAC and Mouse-beta actin: forward 5′-GGCTGTATTCCCCTCCATCG, reverse 5′-CCAGTTGGTAACAATGCCATGT.

### AAV preparation and organoid infection

A pair of short hairpin RNA (shRNA) oligos targeting CDH1 were designed and subcloned into the scAAV.CAG.EGFP.WPREs.U6 vector. The forward oligo sequence was 5’-CACATTCTCGAGAATGTGAGCAATT CTGCTTGGTTTTTTGGTACCCGCGTCGACATTG-3’, and the reverse oligo sequence was 5’-GCTCACATTCTCGAGAATGTGAGCAATTCTGCTTGGCGGTGTTTCGTCCTTTCCACAA-3’. The complementary oligonucleotides encoded a hairpin structure (CCAAGCAGAATTGCTCACATT) derived from the coding sequence of CDH1. The construct was transformed into DH5α competent cells. Ampicillin-resistant colonies were selected and confirmed by DNA sequencing. A scAAV.CAG.EGFP.WPREs.U6.NCshRNA (C-2545, PackGene, Guangzhou, China) was used as control. An ssAAV.EFS.hCDH1.polyA.miniCMV.mCherry.WPRE3-SV40pA vector was constructed to overexpress CDH1 by PackGene. For virus packaging, HEK293 cells were transiently co-transfected with the gene of interest (GOI) plasmid, an adenovirus helper plasmid (pHelper), and an AAV capsid plasmid (pAAV-RC). At 72 h post-transfection, recombinant AAV particles were collected by lysing the cells to release the virus into the supernatant. The crude viral lysate was then purified by iodixanol gradient centrifugation. Viral titers, reported as genome copies per milliliter (GC/mL), were measured by real-time PCR using Universal SYBR Green Mix (Bio-Rad, USA) and primers targeting the inverted terminal repeat (ITR) sequence of the AAV vector. Viruses that met quality control standards were aliquoted and stored at -80 °C until further use.

AAV infection of OE organoids was conducted at Day 7 post culture. Organoids in each Matrigel droplet were infected with 5 × 10^10^ GC of AAV. The virus was removed at 48 h post infection, and organoids were collected for subsequent SARS-CoV-2 infection at Day 5 after AAV infection.

### Measurement of SARS-CoV-2 RNA copies

At Day 0, 3 and 6 post SARS-CoV-2 infection, the virus was inactivated by incubating 80 μL of cell culture supernatant with 240 μL of TRIzol (Vazyme, R411-02). Viral RNA in the samples was quantified using a one-step qRT-PCR assay. Viral RNA was extracted using the Virus DNA/RNA Extraction Kit (Vazyme, RM501) and used as a template for qRT-PCR amplification with the HiScript Ⅱ One-Step qRT-PCR SYBR Green Kit (Vazyme, Q221). The following primer pair was used to target the SARS-CoV-2 nucleocapsid gene: SARS-CoV-2 Np-qF1 (5’-GAGCTACCAGACGAATTCGTGGT-3’)/Np-qR1 (5’-CTCAAGTGTCTGTGGATCACG -3’). PCR was performed according to the manufacturer’s instructions.

### Proximity ligation assay

OE organoid slides were washed twice with PBS and then blocked for 60 min in BSAT (0.4% Triton X-100 in PBS with 5% BSA). Slides were processed using Duolink Proximity Ligation Assay (PLA) reagents (In-Situ PLA Probe Anti-Goat MINUS, Sigma, DUO92006; In-Situ PLA Probe Anti-Mouse PLUS, Sigma, DUO92001; In-Situ Detection Reagents Red, Sigma, DUO92008) according to the manufacturer’s in situ fluorescence protocol. Briefly, slides were blocked in BSAT for 60 min, followed by overnight incubation with primary antibodies diluted in BSAT. Primary antibodies used included anti-CDH1 (1:200, 610182, BD), anti-ACE2, anti-Krt18 (1:200, 10830-1-AP, Proteintech), and anti-Krt14 (1:200, 10143-1-AP, Proteintech). Slides were then incubated with PLA probe solution for 60 min, ligation solution for 30 min, amplification solution for 100 min, and DAPI staining solution (C1006, Beyotime) for 20 min, with washes in the provided buffers between each step. Finally, organoids were mounted in Vectashield (Vector Laboratories). Images were acquired using an SP8 confocal microscope (Leica) with LAS AF Lite software.

### Flow cytometry

For flow cytometry analysis, approximately 5 × 10^5^ HEK293-ACE2 and HEK293-ACE2-CDH1 cells were harvested and washed. Cells were incubated with a primary Goat anti-ACE2 antibody (Cat# AF933, R&D Systems) diluted in FACS buffer (PBS containing 2% FBS) for 2 h at 4 °C. Following the primary incubation, cells were washed twice with FACS buffer. Subsequently, cells were co-stained with an APC-conjugated anti-E-Cadherin (CDH1) antibody (Cat# 147311, BioLegend) and an Alexa Fluor 488-conjugated Donkey anti-Goat IgG secondary antibody (Cat# A-11055, Invitrogen) in FACS buffer for 30 min at 4 °C in the dark. After final washing step, cells were resuspended in FACS buffer containing DAPI to exclude dead cells. Data were acquired using a BD FACSymphony A1 flow cytometer and analyzed using FlowJo software.

For Spike protein binding assays, cells were transfected with AAV6 vectors to manipulate CDH1 expression. At 48 h post transfection, approximately 5 × 10^5^ cells per sample were harvested and washed. Cells were then incubated with recombinant His-tagged Spike proteins, including SARS-CoV GD01 Spike Trimer (Cat# SPN-S52Ht, ACRO Biosystems), SARS-CoV-2 S1 protein (Cat# S1N-C52H3, ACRO Biosystems), and HCoV-NL63 S1 protein (Cat# SIN-V52H3, ACRO Biosystems) diluted in FACS buffer (PBS containing 2% FBS) for 2 h at 4 °C. Following the binding step, cells were washed and incubated with a primary Goat anti-ACE2 antibody (Cat# AF933, R&D Systems) for 1.5 h at 4 °C. Cells were then washed twice with FACS buffer and subsequently co-stained for 30 min at 4 °C in the dark with a mixture of three antibodies including Alexa Fluor 488-conjugated Donkey anti-Goat IgG secondary antibody (Cat# A-11055, Invitrogen), APC-Cy7-conjugated anti-His antibody (Cat# 362633, BioLegend) for Spike protein detection, and APC-conjugated anti-E-Cadherin (CDH1) antibody (Cat# 147311, BioLegend) to concurrently monitor CDH1 expression. After final washing step, cells were resuspended in FACS buffer containing DAPI to exclude dead cells. Data were acquired using a BD FACSymphony A1 flow cytometer and analyzed using FlowJo software.

### Statistics

A double-blinding approach was adopted to eliminate observer bias. Samples were coded by an independent researcher, and the investigators performing image analysis and quantification were blinded to group allocation until all data analyses were finalized. The intensities of protein bands were quantified using ImageJ software (version 1.53 K). Grayscale values were measured by applying a uniform rectangular region of interest to each band. For pseudoviral infection measurements, four randomly selected areas per well were imaged at 24, 48, and 72 h post infection. The numbers of GFP- and mCherry-positive cells were manually counted using ImageJ software. The percentage of GFP/mCherry-positive cells was calculated by dividing the number of GFP/mCherry-positive cells by the number of mCherry-positive cells in the imaged area. For SARS-CoV-2 infection measurements, nine randomly selected areas per well were imaged at 24 and 48 h post infection. The number of GFP-positive cells was counted using ImageJ software, and the percentage of GFP-positive cells was determined by dividing the number of GFP-positive cells by the number of DAPI-positive cells in the imaged area. Data were presented as mean ± SD or SEM using GraphPad Prism (Version 10.4.0) software. Statistical significance was determined by one-way ANOVA with Dunnett’s multiple comparisons test, or by two-way ANOVA with Sidak’s multiple comparisons test. Significance in scRNA-seq data was determined using the unpaired two-sample Wilcoxon rank-sum test from the ggpubr R package (Version 0.6.0). The *P* values lower than 0.05 were considered statistically significant. ns, *, **, ***, and **** indicated not significant, *P* < 0.05, *P* < 0.01, *P *< 0.001, and *P* < 0.0001, respectively.

### Study approval

The procedures of animal breeding and tissue harvesting were approved by the Committee of Laboratory Animals in EENT Hospital, Fudan University (Permit number: IACUC-DWZX-2025-030). The study protocol of human biopsies of the superior nasal turbinate was approved by the ethical committee of EENT Hospital and ethical permission number was 2025106-1. Informed consent was obtained from the male patient for nasal endoscopic surgery.

## Supplementary information

Appendix

Peer Review File

Source data Fig. 2

Source data Fig. 3

Source data Fig. 4

Appendix Figures Source Data

## Data Availability

The sequencing datasets analyzed in this study are publicly available in the Gene Expression Omnibus (GEO) and Figshare repositories without any access restrictions. The specific datasets corresponding to the figure panels are detailed as follows: data supporting Figs. 1A,C,D,F,G, 2A, and Appendix Fig. S1A,B are accessible under GEO accession code GSE139522 (Durante et al, 2020b; Data ref: Durante et al, 2020a); data supporting Figs. 1B, 1C, 1E, 2A, and Appendix Fig. S1C,D under GSE146043 (Tan et al, 2020b; Data ref: Tan et al, 2020a); data supporting Fig. 2C–E and Appendix Fig. S2F under GSE201620 (Finlay et al, 2022b; Data ref: Finlay et al, 2022a); data supporting Fig. 2F–H under GSE176080 (Khan et al, 2021b; Data ref: Khan et al, 2021a); data supporting Fig. 5A–F under GSE166766 (Ravindra et al, 2021b; Data ref: Ravindra et al, 2021a); and data supporting Fig. 6A–E and Appendix Fig. S9A,B under GSE158055 (Ren et al, 2021b; Data ref: Ren et al, 2021b). Additionally, the dataset supporting Fig. 6F has been deposited in the Figshare repository (https://doi.org/10.6084/m9.figshare.12436517). The source data of this paper are collected in the following database record: biostudies:S-SCDT-10_1038-S44319-026-00880-8.
