# Supplementary material for: Cadherin 1 negatively regulates SARS-CoV-2 infection in the olfactory epithelium
Source: EMBO Rep. 2026 Aug 3;27(18):5407–28. doi: 10.1038/s44319-026-00880-8 (PMC13601637; doi:10.1038/s44319-026-00880-8)
Supplement: Supplementary file 1 [file 44319_2026_880_MOESM1_ESM.pdf]

Appendix to

Cadherin 1 Negatively Regulates SARS-CoV-2 Infection in the Olfactory Epithelium

This PDF file includes:

Appendix Figure S1. Expression of ACE2/CDH1/TMPRSS2/FURIN in the OE....**p2**
Appendix Figure S2. SARS-CoV-2 infection in OE organoids....**p3**
Appendix Figure S3. Expression of CDH1 and mCherry in HEK293T-ACE2 cell line transiently expressing four variants (v1-v4) of CDH1.... **p5**
Appendix Figure S4. Localization of mCherry, ACE2 and CDH1 in HEK293T-ACE2 cells transiently expressing CDH1 variants....**p7**
Appendix Figure S5. Images and quantifications of EGFP$^+$ pseudovirus-infected cells in mCherry$^+$ 293T-ACE2 cells transiently overexpressing four variants (v1-v4) of CDH1....**p8**
Appendix Figure S6. Regulation of CDH1 expression in human OE organoids....**p9**
Appendix Figure S7. CDH1 regulates the binding capacity of coronavirus Spike proteins....**p10**
Appendix Figure S8. Quantitative analysis on Co-IP and pull-down assay data....**p11**
Appendix Figure S9. The correlation between viral entry related genes and viral load in lung cells....**p12**

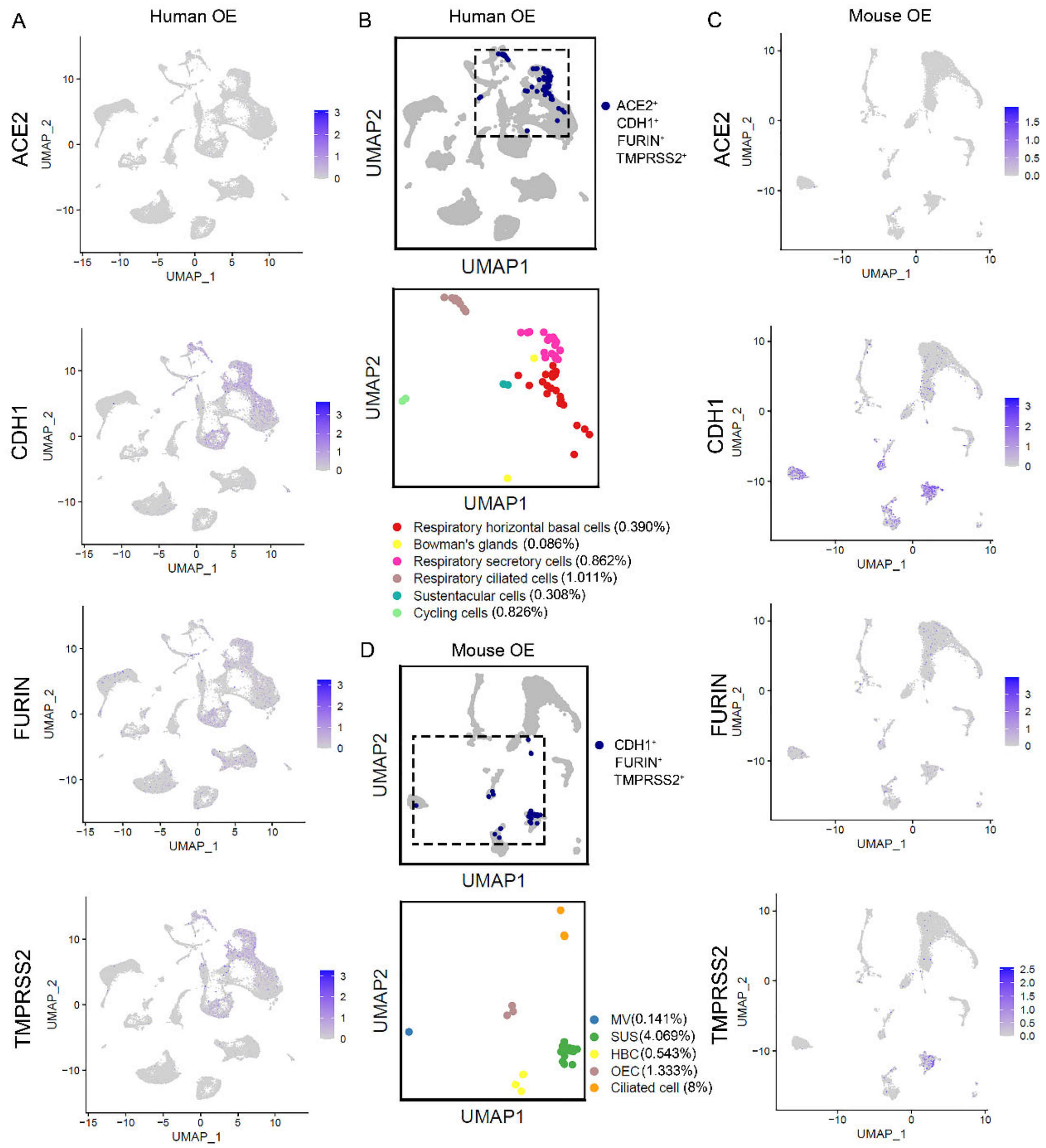

Appendix Figure S1. Expression of ACE2/CDH1/TMPRSS2/FURIN in the OE. (A) UMAP showing expression of ACE2/CDH1/TMPRSS2/FURIN in the human OE. (B) UMAP showing the presence of $ACE2^{+}/CDH1^{+}/TMPRSS2^{+}/FURIN^{+}$ cells in the human OE. UMAP plot at the bottom was the magnification of square by dashed line. (C) UMAP showing expression of ACE2/CDH1/TMPRSS2/FURIN in the mouse OE. (D) The presence of $CDH1^{+}/TMPRSS2^{+}/FURIN^{+}$ cells in the mouse OE. The numbers in parenthesis indicated the percentile co-expression values for various cell types in human (B) and mouse (D) OE.

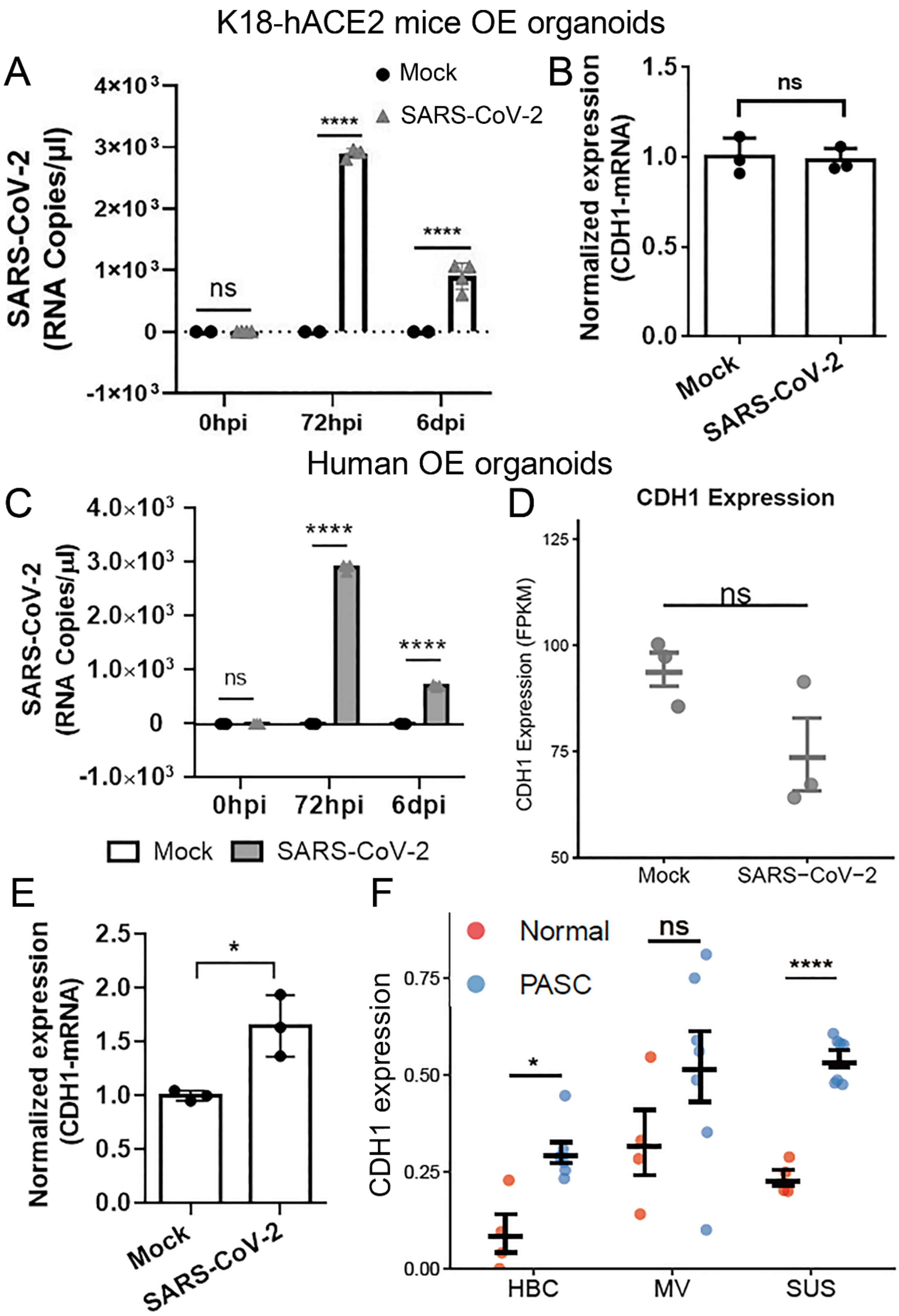

Appendix Figure S2. SARS-CoV-2 infection in OE organoids. (A, C) Measurement of SARS-CoV-2 RNA copies at 72h and 6 days post infection in K18-hACE2 mice (A) and human (C) OE organoids. n =4 in each group (A), n = 3 in each group (C). ns: p >

0.9 (C), **** $p < 0.0001$. (B, E) Quantitative PCR data of CDH1 expression in mock control and infected mouse (B) and human (E) OE organoids at Day 6. n = 3 in each group. ns: $p = 0.7924$ (B), *$p = 0.018$ (E). (D) Relative CDH1 expression in RNA-Seq data of mock control and infected human OE organoids at 3 dpi. ns: $p = 0.482$. (F) Relative CDH1 expression in scRNA-Seq data of OE from normal controls and patients with PASC. Statistical significance was determined by two-way ANOVA with Sidak's multiple comparisons test in (A) and (C), unpaired t test in (B) and (E), Wald test in (D), unpaired t test with Benjamini & Hochberg adjustment in (F). ns: $p = 0.151$, * $p = 0.023$; **** $p = 0.000019$. ns, not significant. Data were presented as mean ± SD.

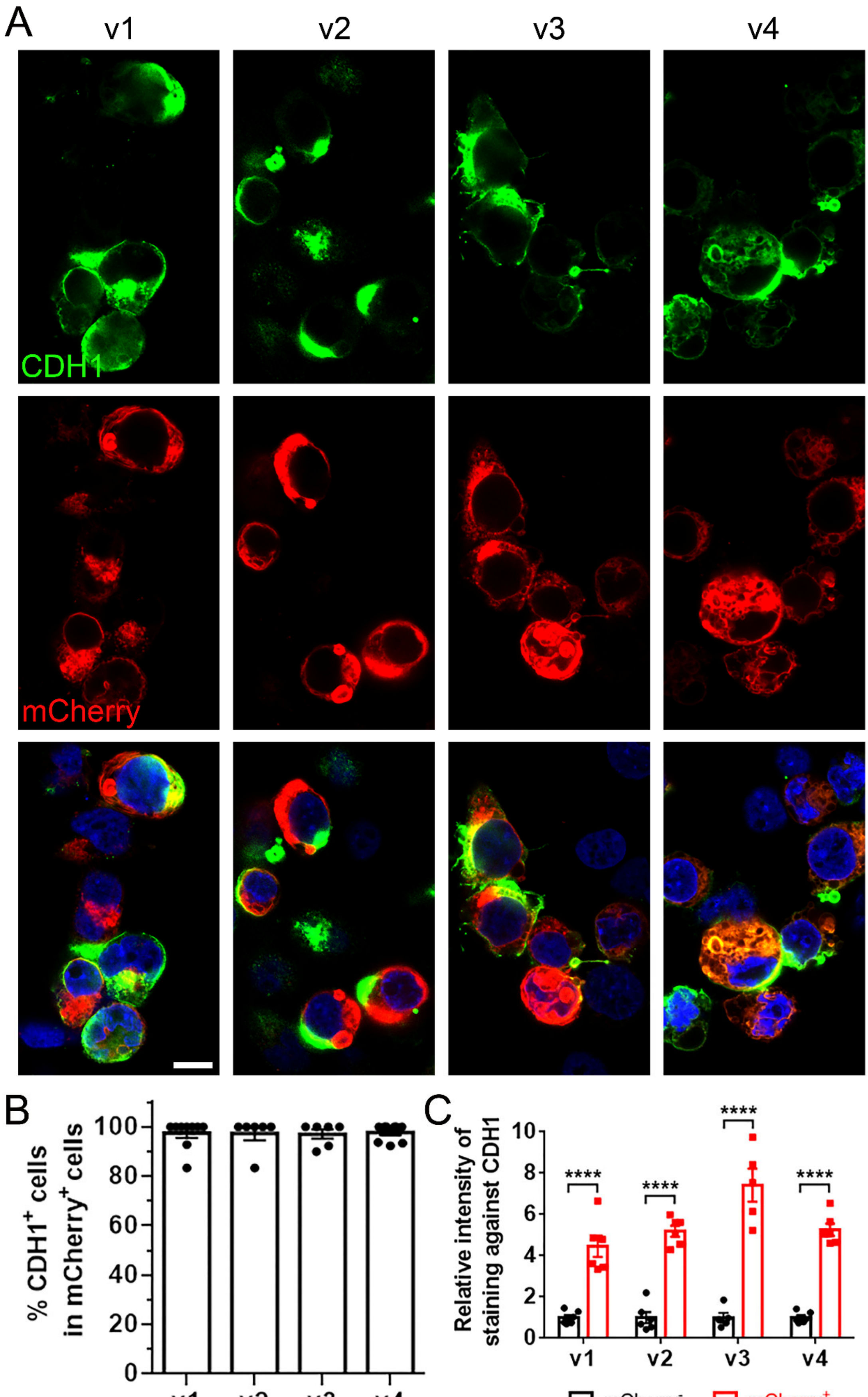

A
v1
v2
v3
v4
CDH1
mCherry
B
% CDH1+ cells in mCherry+ cells
100
80
60
40
20
0
v1
v2
v3
v4
C
Relative intensity of staining against CDH1
10
8
6
4
2
0
****
****
****
****
v1
v2
v3
v4
mCherry-
mCherry+

Appendix Figure S3. Expression of CDH1 and mCherry in HEK293T-ACE2 cell line transiently expressing four variants (v1-v4) of CDH1. (A) Confocal images of CDH1 staining and mCherry signals in HEK293T-ACE2 cells transiently expressing CDH1 variants. (B) Quantification of the percentage of $CDH1^{+}$ cells out of the $mCherry^{+}$ cells. n = 9, 6, 6, 9 technical replicates in v1-v4 groups. (C) Quantification of the relative intensity of CDH1 staining in $mCherry^{+}$ and $mCherry^{-}$ cells. n = 6 replicates in v1, v2 and v4 groups, and n = 5 replicates in v3 group. The statistical difference was determined by two-way ANOVA with Sidak's multiple comparisons test. **** $p < 0.0001$. The CDH1 antibody (BD Transduction Laboratories™, #610181) recognizes the cytoplasmic domain of CDH1 that exists in all four variants. Data were presented as mean ± SEM. Scale bar: 10 µm.

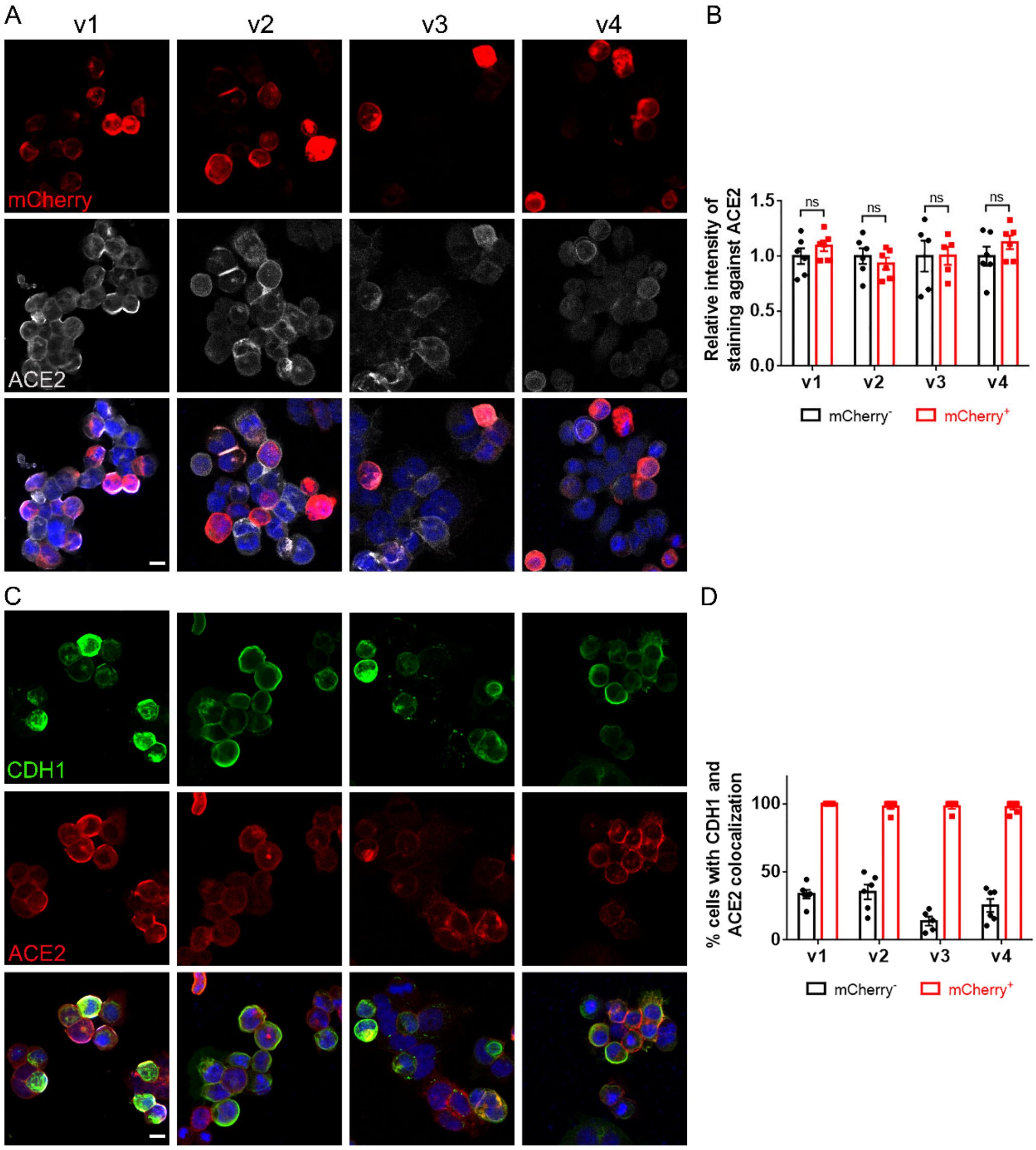

Appendix Figure S4. Localization of mCherry, ACE2 and CDH1 in HEK293T-ACE2 cells transiently expressing CDH1 variants. (A, B) Confocal images (A) and quantification (B) of ACE2 expression intensity in $mCherry^+$ and $mCherry^−$ cells. (C, D) Confocal images and quantification of cells with CDH1 and ACE2 localization. n = 6, 6, 5, 6 technical replicates in v1, v2, v3 and v4 group. The statistical difference was determined by two-way ANOVA with Sidak's multiple comparisons test. ns, not significant. Data were presented as mean ± SEM. Scale bars: 10 μm.

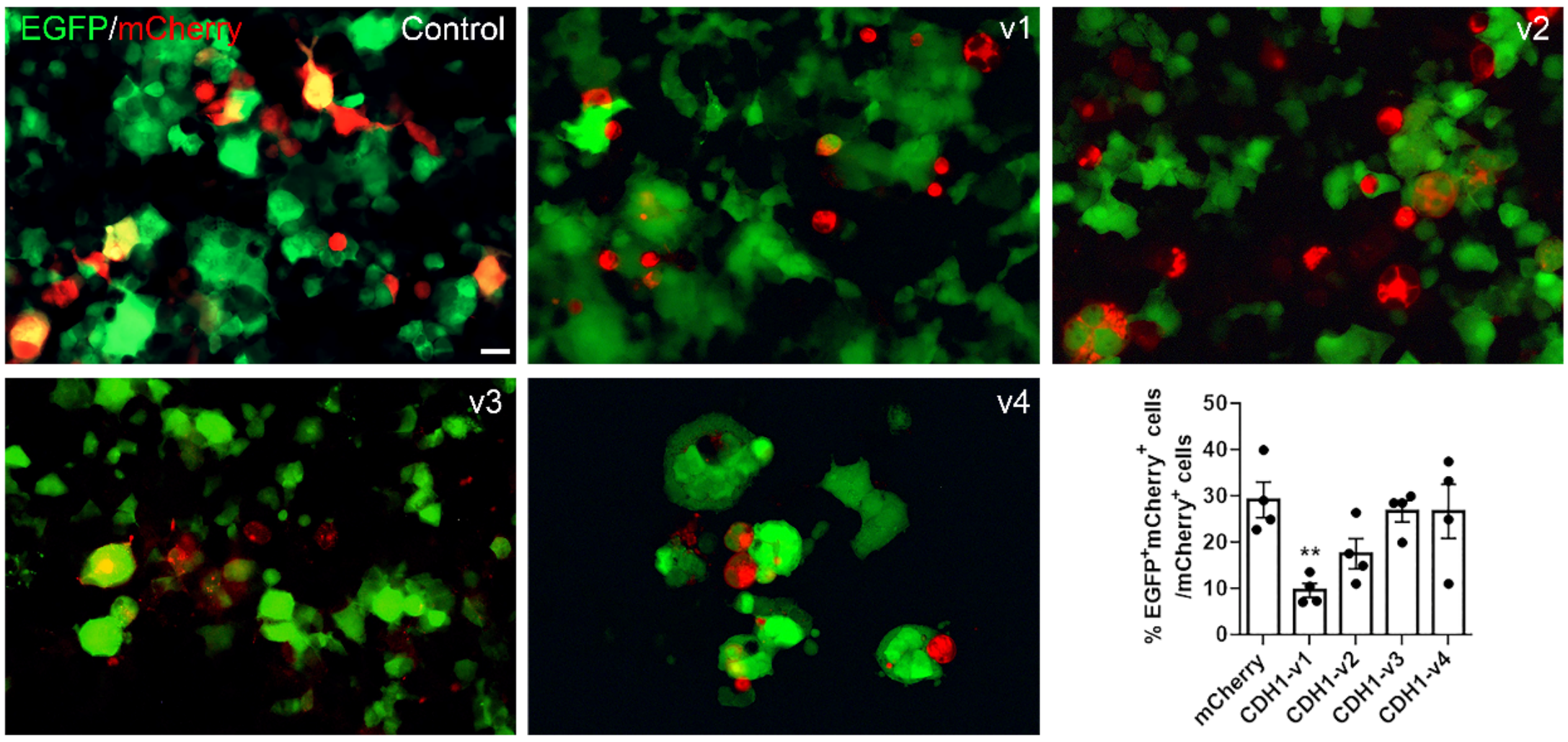

Appendix Figure S5. Images and quantifications of $EGFP^+$ pseudovirus-infected cells in $mCherry^+$ 293T-ACE2 cells transiently overexpressing four variants (v1-v4) of CDH1. Statistical significance was determined by one-way ANOVA with Dunnett's multiple comparisons test. v1: ** $p = 0.0062$, v2: $p = 0.1187$, v3: $p = 0.9693$, v4: $p = 0.967$. $n = 4$ in each group. Data were presented as mean ± SEM. Scale bar: 20 µm.

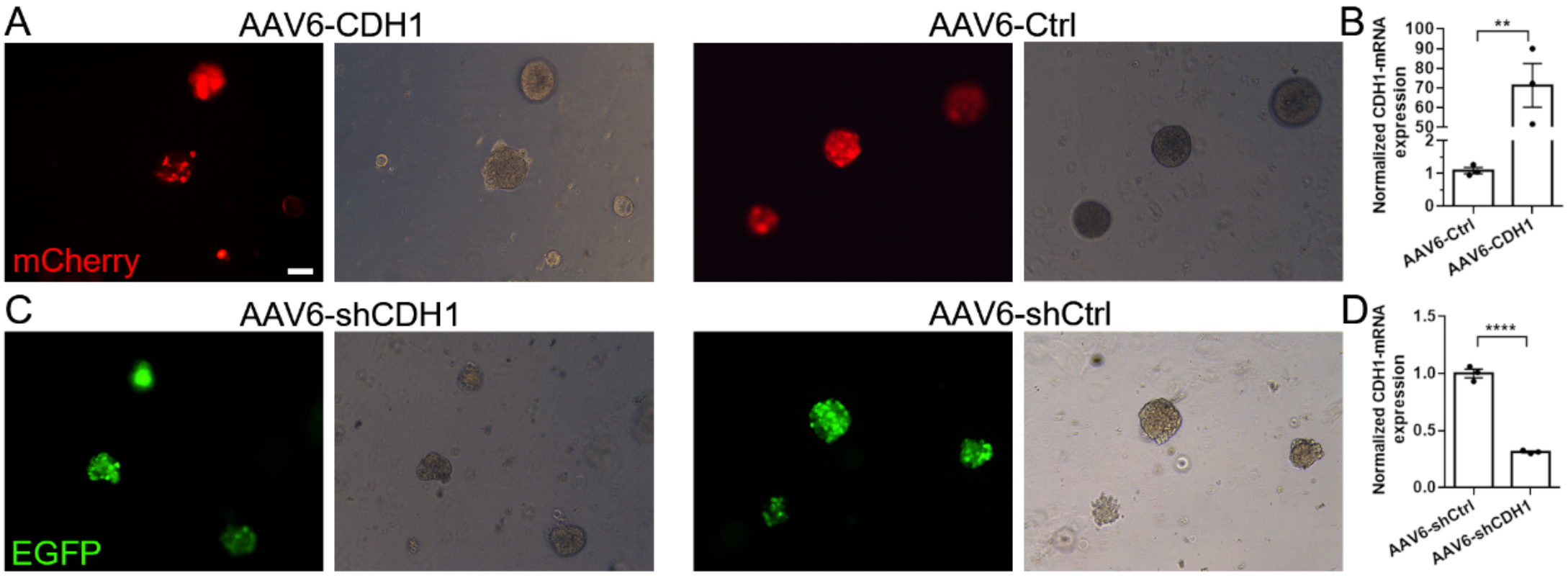

Appendix Figure S6. Regulation of CDH1 expression in human OE organoids. (A, C) Confocal images of organoids infected with AAV-CDH1 (CDH1 overexpression) or AAV6-Ctrl (Control group) (A), and with AAV6-shCDH1 (CDH1 downregulation) or AAV6-shCtrl (Control group) (C). (B, D) Quantitative PCR data showing the significant upregulation (B) or downregulation (D) in organoids by AAV infection. n = 3 in each group. Statistical significance was determined by unpaired t test. ** $p = 0.0032$, **** $p < 0.0001$. Data were presented as mean ± SEM. Scale bar: 100 μm.

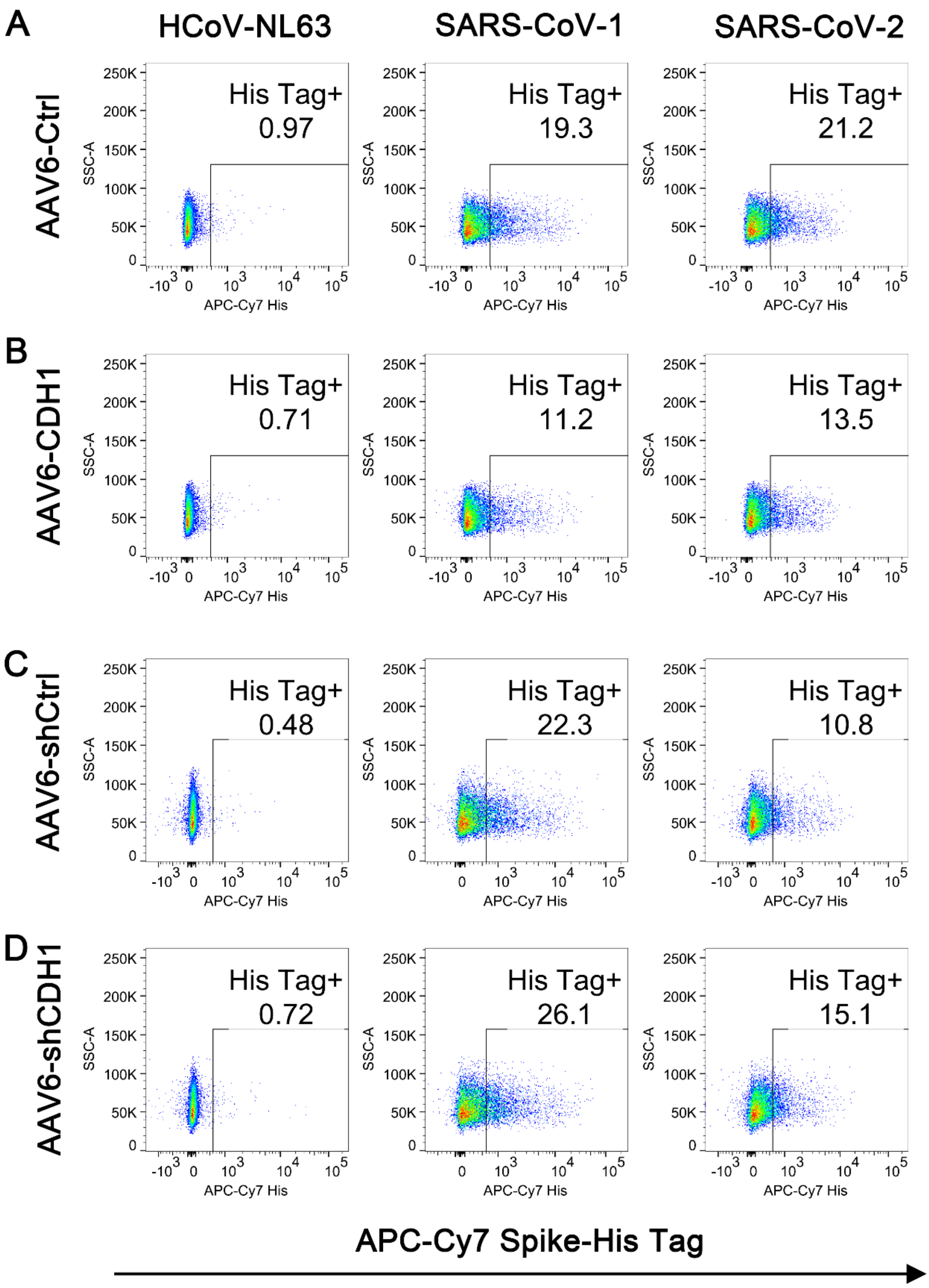

Appendix Figure S7. CDH1 regulates the binding capacity of coronavirus Spike proteins. (A-D) Flow cytometry plots showing the binding efficiency of His-tagged Spike proteins from HCoV-NL63, SARS-CoV-1, and SARS-CoV-2 to HEK293T-ACE2 cells infected with control virus AAV6-Ctrl (A), CDH1 overexpression virus AAV6-CDH1 (B), knockout control virus AAV6-shCtrl (C), and the CDH1 knockout virus AAV6-shCDH1 (D). The numbers within the plots represented the percentage of His Tag positive cell population.

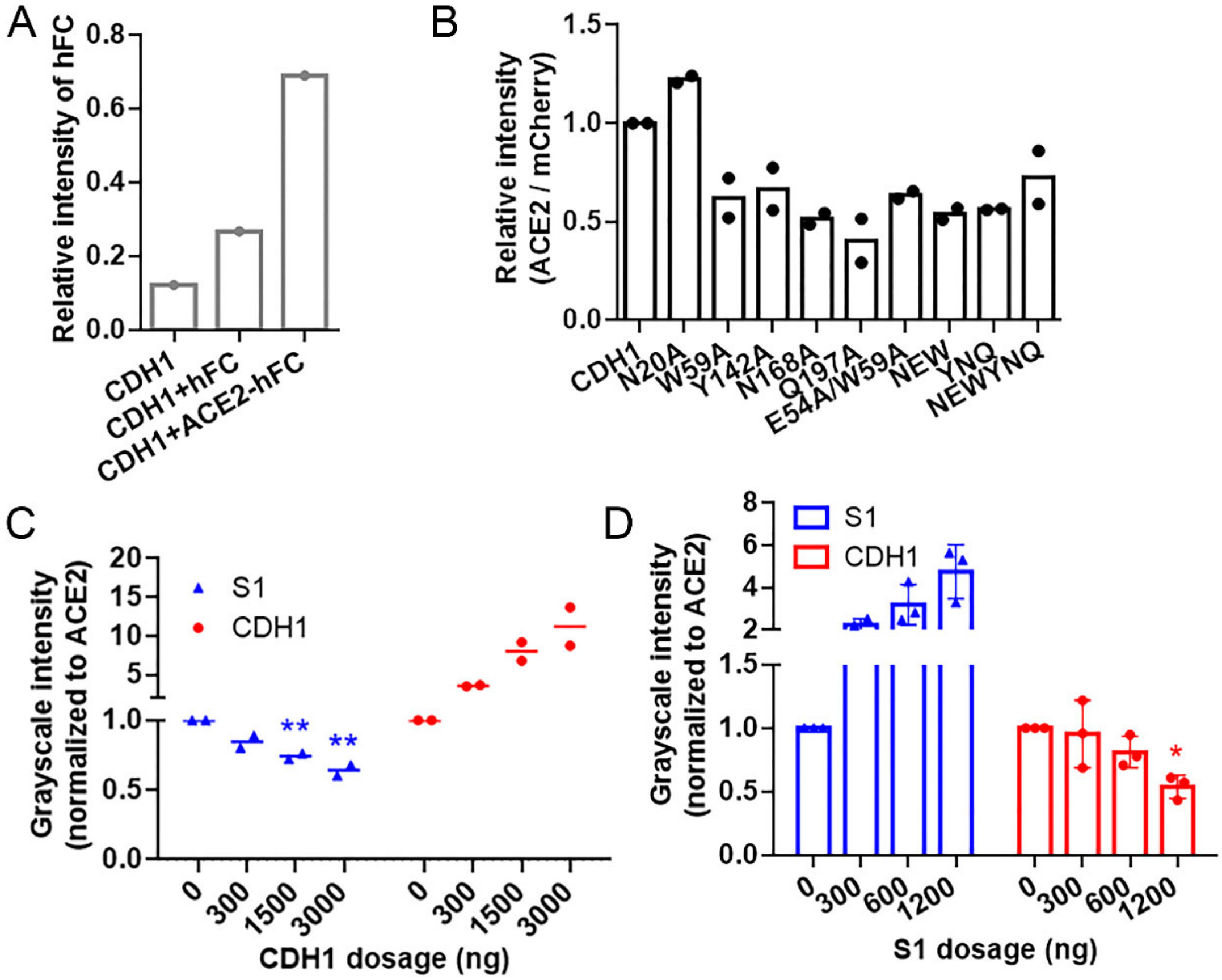

Appendix Figure S8. Quantitative analysis on Co-IP and pull-down assay data. Quantitative analysis on Co-IP of CDH1 and ACE2-hFc fusion protein (A), Co-IP of ACE2 recombinant protein and mutated CDH1 protein (B), and pull-down assay (C, D). Statistical significance was determined by one-way ANOVA with Dunnett's multiple comparisons test. **p = 0.009 (1500 ng CDH1) and **p = 0.0026 (3000 ng CDH1) (C), *p = 0.0156 (D). n = 2 in each group (C), n = 3 in each group (D). Data were presented as mean ± SD.

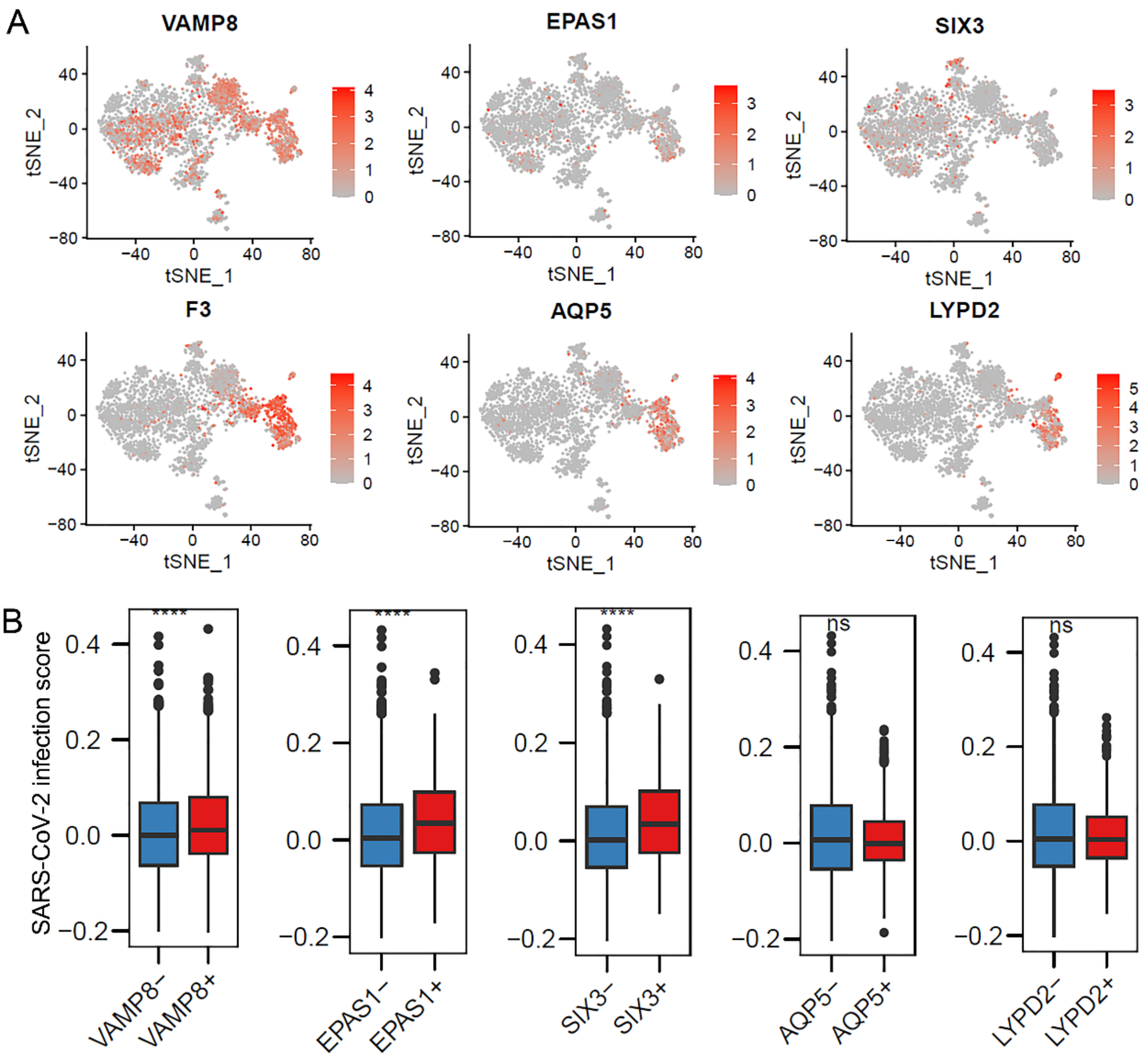

Appendix Figure S9. The correlation between viral entry related genes and viral load in lung cells. (A) tSNE plot showing VAMP8, EPAS1, SIX3, F3, AQP5, LYPD2 expression in infected lung cells. (B) Differential gene set scores of early SARS-CoV-2 infection events between VAMP8^{+} and VAMP8^{-}, EPAS1^{+} and EPAS1^{-}, SIX3^{+} and SIX3^{-}, AQP5^{+} and AQP5^{-}, LYPD2^{+} and LYPD2^{-} infected lung cells. Significance was determined by unpaired two-samples Wilcoxon rank-sum test. ns, not significant. VAMP8: ****p = 9.07E-6, EPAS1: ****p = 1.28E-6, SIX3: ****p = 3.29E-9, AQP5: p = 0.318, LYPD2: p = 0.909.
