# Supplementary material for: Cadherin 1 negatively regulates SARS-CoV-2 infection in the olfactory epithelium
Source: EMBO Rep. 2026 Aug 3;27(18):5407–28. doi: 10.1038/s44319-026-00880-8 (PMC13601637; doi:10.1038/s44319-026-00880-8)
Supplement: Supplementary file 2 [file 44319_2026_880_MOESM2_ESM.pdf]

EMBO reports

Cadherin 1 Negatively Regulates SARS-CoV-2 Infection in the Olfactory Epithelium

Weihao Li, Yuansen Shi, Xuewen Li, Meiqin Liu, Jinxia Liu, Zhen Qin, Haofeng Lin, Yuzhen Wang, and Yiqun Yu

Corresponding author(s): Yiqun Yu (yu_yiqun@fudan.edu.cn)

Review Timeline:

Submission Date:	30th Nov 25
Editorial Decision:	26th Jan 26
Revision Received:	23rd Apr 26
Editorial Decision:	9th Jun 26
Revision Received:	10th Jun 26
Accepted:	3rd Jul 26

Editor: Kurt Weir

Transaction Report:

(Note: With the exception of the correction of typographical or spelling errors that could be a source of ambiguity, letters and reports are not edited. Depending on transfer agreements, referee reports obtained elsewhere may or may not be included in this compilation. Referee reports are anonymous unless the Referee chooses to sign their reports.)

1st Editorial Decision 26th Jan 2026

Dear Prof. Yu

Thank you for the submission of your research manuscript to our journal. We have now received the full set of referee reports that is copied below.

As you have seen, the referees acknowledge that the findings are interesting and that the conclusions are overall supported by the data presented but they also raise a number of concerns and have suggestions how to further strengthen the data, particularly the inclusion of additional controls for the viral entry assays and greater information on the interaction of CDH1 and ACE2 in cells.

Given these constructive comments, we would like to invite you to revise your manuscript with the understanding that the referee concerns (as detailed above and in their reports) must be fully addressed and their suggestions taken on board. Please address all referee concerns in a complete point-by-point response. Acceptance of the manuscript will depend on a positive outcome of a second round of review. It is EMBO Reports policy to allow a single round of revision only and acceptance or rejection of the manuscript will therefore depend on the completeness of your responses included in the next, final version of the manuscript.

We realize that it is difficult to revise to a specific deadline. In the interest of protecting the conceptual advance provided by the work, we recommend a revision within 3 months (April 26th). Please discuss the revision progress ahead of this time with the editor if you require more time to complete the revisions.

I am also happy to discuss the revision further via e-mail or a video call, if you wish.

You can either publish the study as a short report or as a full article. For short reports, the revised manuscript should not exceed 27,000 characters (including spaces but excluding materials & methods and references) and 5 main plus 5 expanded view figures. The results and discussion sections must further be combined, which will help to shorten the manuscript text by eliminating some redundancy that is inevitable when discussing the same experiments twice. For a normal article there are no length limitations, but it should have more than 5 main figures and the results and discussion sections must be separate. In both cases, the entire materials and methods must be included in the main manuscript file.

================
IMPORTANT NOTE:

We perform an initial quality control of all revised manuscripts before re-review. Your manuscript will FAIL this control and the handling will be delayed IN CASE the following APPLIES:

1) A data availability section providing access to data deposited in public databases is missing. If you have not deposited any data, please add a sentence to the data availability section that explains that.

2) Your manuscript contains statistics and error bars based on n=2. Please use scatter blots in these cases. No statistics should be calculated if n=2.

When submitting your revised manuscript, please carefully review the instructions that follow below. Failure to include requested items will delay the evaluation of your revision.

================

When submitting your revised manuscript, we will require:

1) a .docx formatted version of the manuscript text (including legends for main figures, EV figures and tables). Please make sure that the changes are highlighted to be clearly visible.

2) individual production quality figure files as .eps, .tif, .jpg (one file per figure).
Please download our Figure Preparation Guidelines (figure preparation pdf) from our Author Guidelines pages https://www.embopress.org/page/journal/14693178/authorguide for more info on how to prepare your figures.

3) a .docx formatted letter INCLUDING the reviewers' reports and your detailed point-by-point responses to their comments. As part of the EMBO Press transparent editorial process, the point-by-point response is part of the Review Process File (RPF), which will be published alongside your paper.

4) a complete author checklist, which you can download from our author guidelines

(<https://www.embopress.org/page/journal/14693178/authorguide>). Please insert information in the checklist that is also reflected in the manuscript. The completed author checklist will also be part of the RPF.

5) Please note that all corresponding authors are required to supply an ORCID ID for their name upon submission of a revised manuscript (<https://orcid.org/>). Please find instructions on how to link your ORCID ID to your account in our manuscript tracking system in our Author guidelines
(<https://www.embopress.org/page/journal/14693178/authorguide#authorshipguidelines>)

6) We replaced Supplementary Information with Expanded View (EV) Figures and Tables that are collapsible/expandable online. A maximum of 5 EV Figures can be typeset. EV Figures should be cited as 'Figure EV1, Figure EV2" etc... in the text and their respective legends should be included in the main text after the legends of regular figures.

- For the figures that you do NOT wish to display as Expanded View figures, they should be bundled together with their legends in a single PDF file called *Appendix*, which should start with a short Table of Content. Appendix figures should be referred to in the main text as: "Appendix Figure S1, Appendix Figure S2" etc. See detailed instructions regarding expanded view here: <https://www.embopress.org/page/journal/14693178/authorguide#expandedview>

- Additional Tables/Datasets should be labeled and referred to as Table EV1, Dataset EV1, etc. Legends have to be provided in a separate tab in case of .xls files. Alternatively, the legend can be supplied as a separate text file (README) and zipped together with the Table/Dataset file.

7) Before submitting your revision, primary datasets (and computer code, where appropriate) produced in this study need to be deposited in an appropriate public database (see <
https://www.embopress.org/page/journal/14693178/authorguide#dataavailability>).

Please remember to provide a reviewer password if the datasets are not yet public.

The accession numbers and database should be listed in a formal "Data Availability " section (placed after Materials & Method) that follows the model below (see also < https://www.embopress.org/page/journal/14693178/authorguide#dataavailability>). Please note that the Data Availability Section is restricted to new primary data that are part of this study.

Data availability

The datasets (and computer code) produced in this study are available in the following databases:

- RNA-Seq data: Gene Expression Omnibus GSE46843 (https://www.ncbi.nlm.nih.gov/geo/query/acc.cgi?acc=GSE46843)
- [data type]: [name of the resource] [accession number/identifier/doi] ([URL or identifiers.org/DATABASE:ACCESSION])

*** Note - All links should resolve to a page where the data can be accessed. ***

8) At EMBO Press we ask authors to provide source data for the main manuscript figures. You will receive a separate email with instructions for providing source data with your revised manuscript, including how to upload and organize the files.

Additional information on source data and instruction on how to label the files are available
<https://www.embopress.org/page/journal/14693178/authorguide#sourcedata>

9) The journal requires a statement specifying whether or not authors have competing interests (defined as all potential or actual interests that could be perceived to influence the presentation or interpretation of an article). In case of competing interests, this must be specified in your disclosure statement. Further information: https://www.embopress.org/competing-interests

10) Figure legends and data quantification:
The following points must be specified in each figure legend:

- the name of the statistical test used to generate error bars and P values,
- the EXACT p-values,
- the number (n) of independent experiments (please specify technical or biological replicates) underlying each data point,
- the nature of the bars and error bars (s.d., s.e.m.)

- If the data are obtained from n {less than or equal to} 5, show the individual data points in addition to the SD or SEM.
- If the data are obtained from n {less than or equal to} 2, use scatter blots showing the individual data points.

Discussion of statistical methodology can be reported in the materials and methods section, but figure legends should contain a basic description of n, P and the test applied.

See also the guidelines for figure legend preparation:
https://www.embopress.org/page/journal/14693178/authorguide#figureformat

- Please also include scale bars in all microscopy images and define their size in the legend, not the image.

11) Our journal encourages inclusion of *data citations in the reference list* to directly cite datasets that were re-used and obtained from public databases. Data citations in the article text are distinct from normal bibliographical citations and should directly link to the database records from which the data can be accessed. In the main text, data citations are formatted as follows: "Data ref: Smith et al, 2001" or "Data ref: NCBI Sequence Read Archive PRJNA342805, 2017". In the Reference list, data citations must be labeled with "[DATASET]". A data reference must provide the database name, accession number/identifiers and a resolvable link to the landing page from which the data can be accessed at the end of the reference. Further instructions are available at <https://www.embopress.org/page/journal/14693178/authorguide#referencesformat>.

12) All Materials and Methods need to be described in the main text using our 'Structured Methods' format. According to this format, the Methods section includes a Reagents and Tools Table (listing key reagents, experimental models, software and relevant equipment and including their sources and relevant identifiers) followed by a Methods and Protocols section describing the methods, ideally using a step-by-step protocol format. The aim is to facilitate adoption of the methodologies across labs. Please download and fill our Reagents and Tools Table template (.docx), which you can find in our author guidelines: https://www.embopress.org/page/journal/14693178/authorguide#structuredmethods.
When submitting your revised manuscript, please do not include the Reagents and Tools Table in the Methods section of the manuscript but upload it as a separate file choosing the file type "Reagent Table".
An example of a Method paper with Structured Methods can be found here: https://www.embopress.org/doi/10.15252/msb.20178071.

13) As part of the EMBO publication's Transparent Editorial Process, EMBO Reports publishes online a Review Process File to accompany accepted manuscripts. This File will be published in conjunction with your paper and will include the referee reports, your point-by-point response and all pertinent correspondence relating to the manuscript.

You are able to opt out of this by letting the editorial office know (emboreports@embo.org). If you do opt out, the Review Process File link will point to the following statement: "No Review Process File is available with this article, as the authors have chosen not to make the review process public in this case."

We would also welcome the submission of cover suggestions, or motifs to be used by our Graphics Illustrator in designing a cover.

I look forward to seeing a revised form of your manuscript when it is ready and please let me know if you have questions or comments regarding the revision.

Yours sincerely,

Kurt Weir
Editor
EMBO Reports

Referee #1:

In this manuscript, Li-Shi-Li-Lui et al. identified CDH1 (E-cadherin) as a negative regulator of SARS-CoV-2 infection in the olfactory epithelium and other respiratory tissues. The study provides mechanistic, cellular, biochemical, and organoid-based evidence that CDH1 interacts with ACE2 and competes with Spike for binding, thereby restricting viral entry. The authors combined scRNA-seq analyses, structural modeling, immunostaining, mutagenesis, pseudoviral assays, and infection of human and mouse olfactory epithelial (OE) organoids. This comprehensive work resulted in a well-rounded study and dataset.

The strengths of this work includes the identification of CDH1 as a restriction factor for viral entry. The combination of scRNA-seq analysis, organoid models, biochemical assays, and structural modeling provides a robust multi-angle approach. The Mechanistic Evidence of the competition between CDH1 and Spike for ACE2 binding is well supported by mutagenesis, co-IP, and biochemical data. Lastly, clinical integration of multiple patient datasets provide the increases translational potential of the study.

However, several aspects need clarification or strengthening, including mechanistic depth, data quantification rigor, clarity in writing, and the extent to which correlative observations support causal conclusions in vivo. Addressing these issues would

substantially improve impact and interpretability.

Major Weakness to be addressed
- Causality versus correlation in patient tissue: The manuscript concludes that CDH1 suppresses SARS-CoV-2 infection in vivo, but patient data primarily suggests inverse correlations. Viral infection, cell loss, or inflammation may secondarily reduce CDH1 expression. So, the authors should address alternative interpretations and experiments to strengthen the causality in physiologic olfactory epithelial cells.
- Mechanistic ambiguity in OE context: Most functional experiments use HEK293T-ACE2 cells, which do not replicate the architecture or biological context of OE sustentacular cells. Authors should clarify mechanistic models and discuss limitations.
- Inconsistency between organoids and patient data: CDH1 appears upregulated upon infection in human OE organoids, contrasting with patient tissue trends. This discrepancy requires deeper analysis and discussion (1st comment).
- Absence of quantifications: Several qualitative imaging results lack quantification and statistical reporting. Figures involving viral NP staining, immunofluorescence, and co-staining should include quantified data and details regarding replicates.
- Isoform Rationale: The early focus on four CDH1 isoforms leads to eventual concentration on variant 1. The biological relevance of these isoforms should be explained.
- Structural Modeling Interpretation: Docking predictions must be framed as hypothetical. Conclusions regarding binding interfaces should remain cautious unless validated structurally.

Minor Comments
- Improve the clarity and conciseness in the Introduction.
- Please standardize figure labelings, axis descriptions, and statistical annotation.
- Please discuss the omission of gender/sex as a biological variable.

Referee #2:

In this manuscript, Li and coworkers identify Cadherin-1 (CDH1) as a negative regulator of SARS-CoV-2 infection in the olfactory epithelium (OE). By leveraging existing single-cell RNA-sequencing datasets from human and mouse OE, the authors identify a small set of nine genes that co-express with key SARS-CoV-2 entry factors, including ACE2, TMPRSS2, and FURIN, with CDH1 emerging as a central node within this network. CDH1 is co-expressed with ACE2 in sustentacular and other epithelial cell types and its expression inversely correlates with viral load in olfactory, bronchial, and lung epithelial cells from COVID-19 patients. Using pseudovirus assays, the authors show that CDH1 overexpression reduces viral entry, with the strongest effect observed for CDH1 isoform 1, an effect confirmed in cells stably overexpressing CDH1. Consistently, cells expressing CDH1 show reduced susceptibility to infection with authentic SARS-CoV-2. CDH1 function is further assessed in human olfactory epithelium organoids by AAV-mediated overexpression or knockdown, where increased CDH1 levels correlate with reduced viral nucleoprotein signal and viral RNA, while CDH1 depletion is associated with increased infection.
Mechanistically, the authors demonstrate that CDH1 directly interacts with ACE2 and competes with the viral Spike protein for receptor binding. Structure-guided mutagenesis identifies specific CDH1 residues required for ACE2 interaction, and disruption of this interaction restores viral entry. Together, these findings support a model in which CDH1 acts as a host restriction factor that limits SARS-CoV-2 infection by modulating ACE2 accessibility at the cell surface.
Finally, the authors extend their analysis to independent single-cell RNA-sequencing datasets from SARS-CoV-2-infected bronchial epithelial and lung samples, where CDH1 expression shows a negative correlation with viral RNA levels, particularly in ciliated and secretory cell populations, consistent with the trends observed in the olfactory epithelium.
Collectively, the data presented in this manuscript point to CDH1 as a potential restriction factor for SARS-CoV-2 infection in the olfactory epithelium.

Major comments
The manscript would strongly benefit from extensive language editing for clarity. While the scientific background is generally appropriate and well referenced, several sentences are overly long or imprecisely phrased, making the manuscript and experiment difficult to follow. In addition, a few conceptual inaccuracies should be corrected (e.g., the role of TMPRSS2 in Spike-ACE2 binding versus viral entry).
A central limitation of the manuscript concerns the biological interpretation of the proposed CDH1-ACE2 interaction. While the in vitro interaction assays using purified proteins are technically solid and elegant, they remain entirely outside the cellular context. Validation of the CDH1-ACE2 interaction in cells, ideally in OE-derived systems or organoids, would substantially strengthen the mechanistic model. Approaches that preserve spatial information at the membrane, such as proximity ligation assays or comparable techniques, would be particularly informative, whereas standard cellular co-immunoprecipitation would still leave compartmental localization of the proposed interaction unresolved.
Related to this point, the functional interpretation of the entry assays is limited by the lack of direct measurements of ACE2 surface levels. Across pseudovirus and authentic virus infection experiments, the author do not assesses whether CDH1 expression alters ACE2 abundance or localization at the plasma membrane. As a consequence, reduced viral entry could reflect indirect effects on ACE2 expression, trafficking, or membrane accessibility rather than a direct competitive interaction with Spike. This concern is particularly relevant in the stably selected CDH1 cell lines, where antibiotic selection and long-term expression could have influenced receptor levels. Direct assessment of ACE2 surface expression would be necessary to exclude this alternative explanation.

The pseudovirus assays themselves provide limited insight into the relationship between CDH1 expression and viral entry at the single-cell level since the mCherry signals is always used as proxy for CDH1 expression. However, CDH1 protein levels, localization, and heterogeneity are not directly measured, and ACE2 expression, and more importantly, localizatoin, is not monitored in parallel. This limits the interpretability of the entry phenotypes, since presence of mcherry signals does not inform on the correct expression and localization of CDH1 variants.
Finally, the manuscript does not clearly specify the viral strain or variant used in the authors' SARS-CoV-2 infection experiments. Given the known differences in Spike-ACE2 interactions across variants, clarification of viral strains and discussion of the generalizability of the findings across variants would be important. Overall, the manuscript would benefit from a more rigorous mechanistic analysis of the CDH1-ACE2 interaction, which represents a key element of novelty, as well as from the inclusion of additional controls to strengthen the interpretation of the entry and infection assays.

Minor comments
The figure legends for the organoid infection experiments should explicitly report the number of biological replicates and clarify how many independent experiments were performed. In addition, the immunofluorescence data, particularly in organoids, would benefit from quantitative analysis rather than qualitative representation alone, especially given that the current image quality does not allow clear evaluation of the immunofluorescent signals.
In the figure legend, 2H is indicated as a violin plot while a volcano plot is shown.
Figure 2F: the two difference between the two plots is indicated as non-statistically significant, yet in the manuscript is reported that CDH1 is less expressed in orf1ab high respect to orf1ab low.

Referee #3:

Li and colleagues investigated determinants controlling SARS-CoV-2 infection of the olfactory epithelium (OE). In brief, they report that cadherin 1 expression is linked with that of other genes relevant to infection, including ACE2, and that cadherin 1 levels are inversely correlated with viral load in the OE of COVID-19 patients. Further, overexpression of cadherin-1 in a cell line and in OE organoids is shown to reduce SARS-CoV-2 spike driven entry while knockdown increased infection. In addition, cadherin-1 binding to ACE2 is shown with recombinant proteins and cadherin-1 is demonstrated to reduce the interaction between spike and ACE2. Finally, evidence for a negative correlation between cadherin-1 expression in experimentally infected bronchial and lung cells is presented. In sum, the data suggest that cadherin-1 might negatively regulate SARS-CoV-2 infection by interfering with ACE2 engagement by the viral spike protein. These findings are of interest to the field. However, some points remain open.

Major

The negative effect of cadherin 1 on SARS-CoV-2 spike driven entry observed with pseudotypes is suggestive but not fully convincing. It is important to show ACE2 and cadherin-1 expression levels in the cell lines analyzed and to investigate entry driven by other coronavirus spike proteins and unrelated viral glycoproteins as specificity control. Of particular importance will be to include SARS-CoV-1 and NL63 spikes since they also depend on ACE2 for entry but, mainly in case of NL63 spike, engage ACE2 differently as compared to SARS-CoV-2 spike.

The ACE2-spike-cadherin 1 interaction data were obtained with recombinant proteins which might not fully model the corresponding proteins expressed in cells. It should thus be investigated whether directed expression of cadherin-1 in a cell line not only reduces SARS-CoV-2 spike driven entry but also spike binding to cells and whether KO of endogenous cadherin-1 increases spike binding.

Minor

It should be investigated whether the negative effect of cadherin-1 on SARS-CoV-2 entry is observed for the major SARS-CoV-2 variants.

On several occasions patient samples were analyzed for expression of viral and cellular genes. It should be stated in the text at what time the samples were taken and what variants were circulating at that time.

The manuscript requires revision for grammar and style issues.

Here are our point-by-point responses based on the comments, and all responses are highlighted by cyan.

From Editor

We perform an initial quality control of all revised manuscripts before re-review. Your manuscript will FAIL this control and the handling will be delayed IN CASE the following APPLIES:

1) A data availability section providing access to data deposited in public databases is missing. If you have not deposited any data, please add a sentence to the data availability section that explains that.
Response: We did not deposit any data, and we explained this in data availability section as "All data are available in the main text and the supplementary materials. We did not deposit any data".
2) Your manuscript contains statistics and error bars based on n=2. Please use scatter blots in these cases. No statistics should be calculated if n=2.
Response: We used scatter plot when n = 2.
When submitting your revised manuscript, please carefully review the instructions that follow below. Failure to include requested items will delay the evaluation of your revision.

When submitting your revised manuscript, we will require:

1) a .docx formatted version of the manuscript text (including legends for main figures, EV figures and tables). Please make sure that the changes are highlighted to be clearly visible.
Response: We provided.
2) individual production quality figure files as .eps, .tif, .jpg (one file per figure).
Please download our Figure Preparation Guidelines (figure preparation pdf) from our Author Guidelines pages https://www.embopress.org/page/journal/14693178/authorguide for more info on how to prepare your figures.
Response: We uploaded figures as TIF format.
3) a .docx formatted letter INCLUDING the reviewers' reports and your detailed point-by-point responses to their comments. As part of the EMBO Press transparent editorial process, the point-by-point response is part of the Review Process File (RPF), which will be published alongside your paper.
Response: We provided.
4) a complete author checklist, which you can download from our author guidelines (<https://www.embopress.org/page/journal/14693178/authorguide>). Please insert information in the checklist that is also reflected in the manuscript. The completed author checklist will also be part of the RPF.
Response: We provided.
5) Please note that all corresponding authors are required to supply an ORCID ID for their name upon submission of a revised manuscript (<https://orcid.org/>). Please find instructions on how to link your ORCID ID to your account in our manuscript tracking system in our Author guidelines

(<https://www.embopress.org/page/journal/14693178/authorguide#authorshipguidelines>)

Response: We provided.

6) We replaced Supplementary Information with Expanded View (EV) Figures and Tables that are collapsible/expandable online. A maximum of 5 EV Figures can be typeset. EV Figures should be cited as 'Figure EV1, Figure EV2" etc... in the text and their respective legends should be included in the main text after the legends of regular figures.

Response: Not applicable.

- For the figures that you do NOT wish to display as Expanded View figures, they should be bundled together with their legends in a single PDF file called *Appendix*, which should start with a short Table of Content. Appendix figures should be referred to in the main text as: "Appendix Figure S1, Appendix Figure S2" etc. See detailed instructions regarding expanded view here:
<https://www.embopress.org/page/journal/14693178/authorguide#expandedview>

Response: We provided.

- Additional Tables/Datasets should be labeled and referred to as Table EV1, Dataset EV1, etc. Legends have to be provided in a separate tab in case of .xls files. Alternatively, the legend can be supplied as a separate text file (README) and zipped together with the Table/Dataset file.

Response: Not applicable.

7) Before submitting your revision, primary datasets (and computer code, where appropriate) produced in this study need to be deposited in an appropriate public database (see < https://www.embopress.org/page/journal/14693178/authorguide#dataavailability>).
Please remember to provide a reviewer password if the datasets are not yet public.
The accession numbers and database should be listed in a formal "Data Availability " section (placed after Materials & Method) that follows the model below (see also < https://www.embopress.org/page/journal/14693178/authorguide#dataavailability>).
Please note that the Data Availability Section is restricted to new primary data that are part of this study.

Response: Not applicable.

8) At EMBO Press we ask authors to provide source data for the main manuscript figures. You will receive a separate email with instructions for providing source data with your revised manuscript, including how to upload and organize the files.
Additional information on source data and instruction on how to label the files are available <https://www.embopress.org/page/journal/14693178/authorguide#sourcedata>

Response: We provided.

9) The journal requires a statement specifying whether or not authors have competing interests (defined as all potential or actual interests that could be perceived to influence the presentation or interpretation of an article). In case of competing interests, this must be specified in your disclosure statement. Further information: https://www.embopress.org/competing-interests

Response: We provided.

10) Figure legends and data quantification:
The following points must be specified in each figure legend:

- the name of the statistical test used to generate error bars and P values,
- the EXACT p-values,
- the number (n) of independent experiments (please specify technical or biological replicates) underlying each data point,
- the nature of the bars and error bars (s.d., s.e.m.)
- If the data are obtained from n {less than or equal to} 5, show the individual data points in addition to the SD or SEM.
- If the data are obtained from n {less than or equal to} 2, use scatter blots showing the individual data points.

Discussion of statistical methodology can be reported in the materials and methods section, but figure legends should contain a basic description of n, P and the test applied.

See also the guidelines for figure legend preparation: https://www.embopress.org/page/journal/14693178/authorguide#figureformat

- Please also include scale bars in all microscopy images and define their size in the legend, not the image.

Response: We specified all these in each figure legend.

11) Our journal encourages inclusion of *data citations in the reference list* to directly cite datasets that were re-used and obtained from public databases. Data citations in the article text are distinct from normal bibliographical citations and should directly link to the database records from which the data can be accessed. In the main text, data citations are formatted as follows: "Data ref: Smith et al, 2001" or "Data ref: NCBI Sequence Read Archive PRJNA342805, 2017". In the Reference list, data citations must be labeled with "[DATASET]". A data reference must provide the database name, accession number/identifiers and a resolvable link to the landing page from which the data can be accessed at the end of the reference. Further instructions are available at <https://www.embopress.org/page/journal/14693178/authorguide#referencesformat>.

Response: Not applicable.

12) All Materials and Methods need to be described in the main text using our 'Structured Methods' format. According to this format, the Methods section includes a Reagents and Tools Table (listing key reagents, experimental models, software and relevant equipment and including their sources and relevant identifiers) followed by a Methods and Protocols section describing the methods, ideally using a step-by-step protocol format. The aim is to facilitate adoption of the methodologies across labs.

Please download and fill our Reagents and Tools Table template (.docx), which you can find in our author guidelines: https://www.embopress.org/page/journal/14693178/authorguide#structuredmethods.

When submitting your revised manuscript, please do not include the Reagents and Tools Table in the Methods section of the manuscript but upload it as a separate file choosing the file type "Reagent Table".

An example of a Method paper with Structured Methods can be found here: https://www.embopress.org/doi/10.15252/msb.20178071.

Response: We provided.

13) As part of the EMBO publication's Transparent Editorial Process, EMBO Reports publishes online a Review Process File to accompany accepted manuscripts. This File will be published

in conjunction with your paper and will include the referee reports, your point-by-point response and all pertinent correspondence relating to the manuscript.

Response: We agreed.

Referee 1

In this manuscript, Li-Shi-Li-Lui et al. identified CDH1 (E-cadherin) as a negative regulator of SARS-CoV-2 infection in the olfactory epithelium and other respiratory tissues. The study provides mechanistic, cellular, biochemical, and organoid-based evidence that CDH1 interacts with ACE2 and competes with Spike for binding, thereby restricting viral entry. The authors combined scRNA-seq analyses, structural modeling, immunostaining, mutagenesis, pseudoviral assays, and infection of human and mouse olfactory epithelial (OE) organoids. This comprehensive work resulted in a well-rounded study and dataset.

The strengths of this work includes the identification of CDH1 as a restriction factor for viral entry. The combination of scRNA-seq analysis, organoid models, biochemical assays, and structural modeling provides a robust multi-angle approach. The Mechanistic Evidence of the competition between CDH1 and Spike for ACE2 binding is well supported by mutagenesis, co-IP, and biochemical data. Lastly, clinical integration of multiple patient datasets provide the increases translational potential of the study.

However, several aspects need clarification or strengthening, including mechanistic depth, data quantification rigor, clarity in writing, and the extent to which correlative observations support causal conclusions in vivo. Addressing these issues would substantially improve impact and interpretability.

Major Weakness to be addressed

- Causality versus correlation in patient tissue: The manuscript concludes that CDH1 suppresses SARS-CoV-2 infection in vivo, but patient data primarily suggests inverse correlations. Viral infection, cell loss, or inflammation may secondarily reduce CDH1 expression. So, the authors should address alternative interpretations and experiments to strengthen the causality in physiologic olfactory epithelial cells.

Response: We appreciated the comment. To answer if viral infection reduces the CDH1 expression, we analyzed the single-cell data for human bronchial epithelial cells [GSE166766 reported in (Ravindra *et al*, 2021)] and measured the changes in CDH1 expression at Day 1, 2, and 3 post infection. We found that there was no significant change in CDH1 expression with the progression of infection, as shown in the figure at right side.

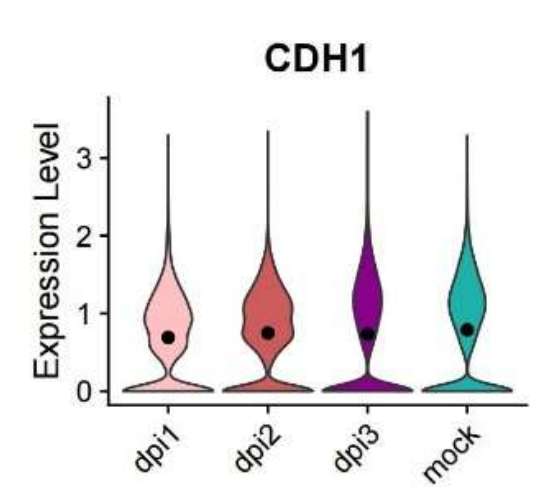

We also infected the Krt18-hACE2 mouse and human olfactory epithelium organoids with SARS-CoV2, and determine the change in CDH1 expression after viral infection. Quantitative PCR data showed that CDH1-mRNA expression level was not significantly decreased in SARS-CoV2-infected organoids at Day 7 post infection when compared to mock control (Appendix Fig.S2).

We performed RNA-Seq on mock control and SARS-CoV-2-infected human olfactory epithelium organoids at 3 dpi, and the result showed that there was not a significant difference in CDH1 expression between control and infected organoids. The data was shown as Appendix Figure S2D.

As we showed in Appendix Fig.S2E, SARS-CoV-2 infection even increase the CDH1 expression in human olfactory epithelium organoid at 6 dpi. This probably reflects an offset of the cellular recovery after viral infection for a relative long time. To prove this, we analyzed the scRNA-Seq data from normal controls and patients with post-acute sequelae of COVID-19 (PASC)(Finlay *et al*, 2022), and found higher CDH1 expression in HBCs and SUS cells of PASC patients compared to normal controls (Appendix Fig.S2F). This further suggests a potential offset of CDH1 expression in the recovery after SARS-CoV-2 infection. However, this inference needs further experimental verification.

Collectively, we hereby conclude that viral infection does not reduce CDH1 expression.

- Mechanistic ambiguity in OE context: Most functional experiments use HEK293T-ACE2 cells, which do not replicate the architecture or biological context of OE sustentacular cells. Authors should clarify mechanistic models and discuss limitations.

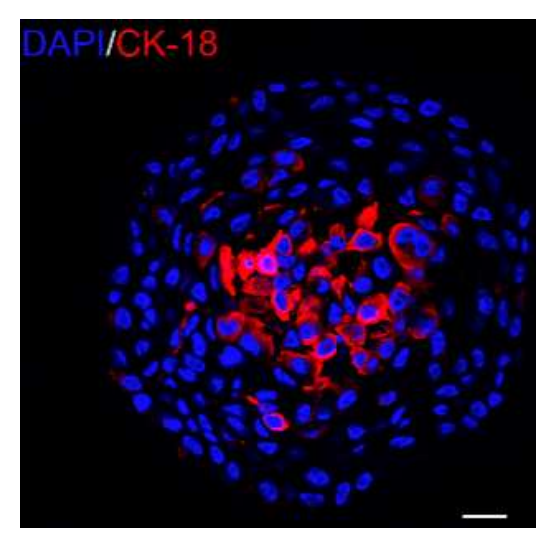

Response: We appreciated the comment. Both HEK293T-ACE2 and human OE organoids were used to determine the regulatory role of CDH1 in viral infection. As shown in the figure at right side, the immunostaining data have shown that human OE organoids contained $CK18^{+}$ sustentacular cells, which were reported to be the main targets to SARS-CoV-2 infection. We discussed the limitations for both models in the second paragraph of Discussion section.

- Inconsistency between organoids and patient data: CDH1 appears upregulated upon infection in human OE organoids, contrasting with patient tissue trends. This discrepancy requires deeper analysis and discussion (1st comment).

Response: We appreciated the comment. The patient data supported a negative correlation between CDH1 expression and viral load in OE cells (Fig.2G). Then, we performed quantitative PCR to further show that viral infection did not significantly decrease the CDH1 expression in mouse organoids (Appendix Fig.S2B). At 3 dpi, RNA-Seq data confirm a non-significant difference in CDH1 expression between mock control and SARS-CoV-2-infected human organoids (Appendix Fig.S2D). However, as you mentioned, viral infection at 6 dpi increased CDH1 expression in human organoids (Appendix Fig.S2E). We also found the CDH1 upregulation in the OE of patients with post-acute sequelae of COVID-19 (PASC) compared to normal control (Appendix Fig.S2F). This suggests that SARS-CoV-2 infection does not hamper the CDH1 expression, and we infer that there is an offsetting effect for CDH1 expression with the progression of viral infection. This needs further experimental verification.

- Absence of quantifications: Several qualitative imaging results lack quantification and statistical reporting. Figures involving viral NP staining, immunofluorescence, and co-staining

should include quantified data and details regarding replicates.

Response: We added the quantification for immunostaining figures and provided n number in figure legends (Fig.2B, 3I, 3J).

- Isoform Rationale: The early focus on four CDH1 isoforms leads to eventual concentration on variant 1. The biological relevance of these isoforms should be explained.

Response: We appreciated the comment. Four human CDH1 transcripts were cloned and overexpressed in HEK-293T-ACE2 cells. Transcript variant 1 has 4811 bp and encodes the longest isoform with 882 amino acids. The variant 1 encodes a preproprotein that is proteolytically processed to generate the mature glycoprotein. The variant 2 has 4628 bp and the isoform 2 contains 821 amino acids, lacking an alternate exon in the central coding region compared to variant 1. The isoform 2 undergoes proteolytic processing similar to isoform 1. The variant 3 has 4878 bp, but the isoform 3 only contains 366 amino acids. The variant 3 uses an alternate splice site in an exon in its 5' UTR, resulting in a different 5' UTR and the use of a downstream start site compared to variant 1. The encoded isoform 3 has a shorter N-terminus and lacks a predicted signal peptide compared to isoform 1. The variant 4 has 4665 bp, and the encoded isoform 4 contains 227 amino acids. This transcript also lacks an exon in its 5' UTR, and the encoded isoform 4 has a shorter N-terminus and lacks a predicted signal peptide. Our data show that overexpression of variant 1 inhibits pseudoviral infection in HEK-293T-ACE2 cells, while other three variants do not exhibit the similar effect (Appendix Fig.S5). Thus, we selected variant 1 for further functional analysis.

- Structural Modeling Interpretation: Docking predictions must be framed as hypothetical. Conclusions regarding binding interfaces should remain cautious unless validated structurally.

Response: We stated the related data as hypothetical since the binding is predictive.

Minor Comments

- Improve the clarity and conciseness in the Introduction.

Response: We improved.

- Please standardize figure labelings, axis descriptions, and statistical annotation.

Response: We standardized in figures and legends.

- Please discuss the omission of gender/sex as a biological variable.

Response: The statement was not accurate and we deleted it.

Referee 2

In this manuscript, Li and coworkers identify Cadherin-1 (CDH1) as a negative regulator of SARS-CoV-2 infection in the olfactory epithelium (OE). By leveraging existing single-cell RNA-sequencing datasets from human and mouse OE, the authors identify a small set of nine genes that co-express with key SARS-CoV-2 entry factors, including ACE2, TMPRSS2, and FURIN, with CDH1 emerging as a central node within this network. CDH1 is co-expressed with ACE2 in sustentacular and other epithelial cell types and its expression inversely correlates with viral load in olfactory, bronchial, and lung epithelial cells from COVID-19 patients.

Using pseudovirus assays, the authors show that CDH1 overexpression reduces viral entry, with the strongest effect observed for CDH1 isoform 1, an effect confirmed in cells stably

overexpressing CDH1. Consistently, cells expressing CDH1 show reduced susceptibility to infection with authentic SARS-CoV-2. CDH1 function is further assessed in human olfactory epithelium organoids by AAV-mediated overexpression or knockdown, where increased CDH1 levels correlate with reduced viral nucleoprotein signal and viral RNA, while CDH1 depletion is associated with increased infection.

Mechanistically, the authors demonstrate that CDH1 directly interacts with ACE2 and competes with the viral Spike protein for receptor binding. Structure-guided mutagenesis identifies specific CDH1 residues required for ACE2 interaction, and disruption of this interaction restores viral entry. Together, these findings support a model in which CDH1 acts as a host restriction factor that limits SARS-CoV-2 infection by modulating ACE2 accessibility at the cell surface.

Finally, the authors extend their analysis to independent single-cell RNA-sequencing datasets from SARS-CoV-2-infected bronchial epithelial and lung samples, where CDH1 expression shows a negative correlation with viral RNA levels, particularly in ciliated and secretory cell populations, consistent with the trends observed in the olfactory epithelium.

Collectively, the data presented in this manuscript point to CDH1 as a potential restriction factor for SARS-CoV-2 infection in the olfactory epithelium.

Major comments

The manscript would strongly benefit from extensive language editing for clarity. While the scientific background is generally appropriate and well referenced, several sentences are overly long or imprecisely phrased, making the manuscript and experiment difficult to follow. In addition, a few conceptual inaccuracies should be corrected (e.g., the role of TMPRSS2 in Spike-ACE2 binding versus viral entry).

Response: We did the language editing and corrected the conceptual inaccuracies.

A central limitation of the manuscript concerns the biological interpretation of the proposed CDH1-ACE2 interaction. While the in vitro interaction assays using purified proteins are technically solid and elegant, they remain entirely outside the cellular context. Validation of the CDH1-ACE2 interaction in cells, ideally in OE-derived systems or organoids, would substantially strengthen the mechanistic model. Approaches that preserve spatial information at the membrane, such as proximity ligation assays or comparable techniques, would be particularly informative, whereas standard cellular co-immunoprecipitation would still leave compartmental localization of the proposed interaction unresolved.

Response: We appreciated the comment and this point is critical to strength the mechanistic model. To validate the CDH1-ACE2 interaction in a physiological context, we conducted proximity ligation assay (PLA) on human OE organoids. In the PLA, target proteins are recognized by specific primary antibodies, and subsequent binding of oligonucleotide-conjugated secondary antibodies in extremely close spatial proximity allows for probe ligation and amplification, generating a visible fluorescent signal. Primary antibodies targeting CDH1 and ACE2 (mouse anti-CDH1 and goat anti-ACE2) coupled with their corresponding Duolink PLA probes (Anti-Goat MINUS DUO92006 and Anti-Mouse PLUS DUO92001; Sigma) were employed to detect their physical proximity. To detect nonspecific binding of PLA probes in the system, we set up a negative control omitting all primary antibodies. Our data showed that co-incubation with primary antibodies against both ACE2 and

CDH1 yielded abundant red fluorescent puncta, whereas the negative control group did not show any observable PLA signal. Thus, the presence of specific PLA signals in human OE organoids provides direct evidence showing an interaction between the two proteins in a real OE system. This further validated the interaction between CDH1 and ACE2 inside the cellular context. The PLA data was shown as Figure 4K-M.

Related to this point, the functional interpretation of the entry assays is limited by the lack of direct measurements of ACE2 surface levels. Across pseudovirus and authentic virus infection experiments, the author do not assess whether CDH1 expression alters ACE2 abundance or localization at the plasma membrane. As a consequence, reduced viral entry could reflect indirect effects on ACE2 expression, trafficking, or membrane accessibility rather than a direct competitive interaction with Spike. This concern is particularly relevant in the stably selected CDH1 cell lines, where antibiotic selection and long-term expression could have influenced receptor levels. Direct assessment of ACE2 surface expression would be necessary to exclude this alternative explanation.

Response: We appreciated the comment and this point is important to elucidate whether CDH1 overexpression alters ACE2 expression. To address this concern, we performed ACE2 antibody staining on non-permeabilized CDH1-overexpressing and control HEK293T-ACE2 cells. We then quantified the Mean Fluorescence Intensity (MFI) of ACE2 surface expression using flow cytometry, and found that there was no significant difference in MFI of ACE2 between the HEK293T-ACE2 and HEK293T-ACE2-CDH1 cells. Thus, we conclude that CDH1 overexpression restricts viral entry without downregulating ACE2 surface expression. These data were shown as Figure 3M, N.

The pseudovirus assays themselves provide limited insight into the relationship between CDH1 expression and viral entry at the single-cell level since the mCherry signals is always used as proxy for CDH1 expression. However, CDH1 protein levels, localization, and heterogeneity are not directly measured, and ACE2 expression, and more importantly, localizatoin, is not monitored in parallel. This limits the interpretability of the entry phenotypes, since presence of mcherry signals does not inform on the correct expression and localization of CDH1 variants.

Response: We appreciated the comment. To address this concern, we performed immunostaining to visualize the colocalization of CDH1 and mCherry reporter in individual mCherry-positive cells. Since CDH1 and mCherry is fused together to express a protein complex, they are theoretically colocalized. When HEK293T-ACE2 cells transiently expressed one of the four CDH1 variants, the immunostaining result indicated that the vast majority of $mCherry^+$ cells showed robust CDH1 expression, and these two were colocalized (Appendix Fig.S3A, B). Besides, intensity of CDH1 expression in $mCherry^+$ cells was significantly higher than $mCherry^-$ cells (Appendix Fig.S3C). These data suggest that the presence of mCherry indicates the strong CDH1 protein expression.

We also performed immunostaining against ACE2 in HEK293T-ACE2 cells transiently expressing one of the four CDH1 variants. The data showed that ACE2 was abundant in the cell membrane, and CDH1 overexpression did not change the ACE2 expression since $mCherry^-$ and $mCherry^+$ cells exhibited similar intensity of ACE2 expression (Appendix Fig.S4A, B). Meanwhile, immunostaining against CDH1 showed the colocalization of CDH1 and ACE2 in almost all $mCherry^+$ HEK293T-ACE2 cells (Appendix Fig.S4C, D). These data elucidated the expression and

localization of CDH1 and ACE2 in HEK293T-ACE2 cells transiently expressing CDH1 variants.

Finally, the manuscript does not clearly specify the viral strain or variant used in the authors' SARS-CoV-2 infection experiments. Given the known differences in Spike-ACE2 interactions across variants, clarification of viral strains and discussion of the generalizability of the findings across variants would be important.

Response: We thank the reviewer for raising this important point. In response, we have now clearly specified the viral strain used in our infection experiments in the revised manuscript (see 'Methods – SARS-CoV-2 infection' section. The SARS-CoV-2 (#IVCAS 6.7512) was offered by the National Virus Resource Center of the Wuhan Institute of Virology, Chinese Academy of Science. The complete genome sequence of the SARS-CoV-2 isolate WIV04 is available under GenBank accession number MN996528.1. This strain represents an early prototypic sequence of the virus.

Regarding the generalizability of our findings, we agree with the reviewer that other variants can potentially alter viral entry efficiency. Since our current study provides fundamental mechanistic insights based on the prototype strain, we have added a discussion of this limitation in the revised manuscript (the last paragraph of Discussion section). Future studies employing pseudoviruses or authentic viruses carrying mutations found in current circulating variants (e.g., Delta, Omicron sublineages) would be valuable to confirm whether the observed mechanisms are conserved across different strains.

Overall, the manuscript would benefit from a more rigorous mechanistic analysis of the CDH1-ACE2 interaction, which represents a key element of novelty, as well as from the inclusion of additional controls to strengthen the interpretation of the entry and infection assays.

Response: We performed above-mentioned assays to further strengthen and support our conclusion.

Minor comments

The figure legends for the organoid infection experiments should explicitly report the number of biological replicates and clarify how many independent experiments were performed. In addition, the immunofluorescence data, particularly in organoids, would benefit from quantitative analysis rather than qualitative representation alone, especially given that the current image quality does not allow clear evaluation of the immunofluorescent signals.

Response: We appreciated the comment. We added the number of replicates in the figure legends. Quantitative analysis was added for the immunostaining data in organoids (Figs.3I and 3J).

In the figure legend, 2H is indicated as a violin plot while a volcano plot is shown.

Figure 2F: the two difference between the two plots is indicated as non-statistically significant, yet in the manuscript is reported that CDH1 is less expressed in orf1ab high respect to orf1ab low.

Response: We corrected them.

Referee 3

Li and colleagues investigated determinants controlling SARS-CoV-2 infection of the

olfactory epithelium (OE). In brief, they report that cadherin 1 expression is linked with that of other genes relevant to infection, including ACE2, and that cadherin 1 levels are inversely correlated with viral load in the OE of COVID-19 patients. Further, overexpression of cadherin-1 in a cell line and in OE organoids is shown to reduce SARS-CoV-2 spike driven entry while knockdown increased infection. In addition, cadherin-1 binding to ACE2 is shown with recombinant proteins and cadherin-1 is demonstrated to reduce the interaction between spike and ACE2. Finally, evidence for a negative correlation between cadherin-1 expression in experimentally infected bronchial and lung cells is presented. In sum, the data suggest that cadherin-1 might negatively regulate SARS-CoV-2 infection by interfering with ACE2 engagement by the viral spike protein. These findings are of interest to the field. However, some points remain open.

Major

The negative effect of cadherin 1 on SARS-CoV-2 spike driven entry observed with pseudotypes is suggestive but not fully convincing. It is important to show ACE2 and cadherin-1 expression levels in the cell lines analyzed and to investigate entry driven by other coronavirus spike proteins and unrelated viral glycoproteins as specificity control. Of particular importance will be to include SARS-CoV-1 and NL63 spikes since they also depend on ACE2 for entry but, mainly in case of NL63 spike, engage ACE2 differently as compared to SARS-CoV-2 spike.

Response: We appreciated the comment and this point is critical to strengthen our conclusion. Firstly, we supplemented immunostaining data showing the colocalization of ACE2 and CDH1 in HEK293T-ACE2 cells expressing one of the four CDH1 variants (Appendix Figure S4C, D). Secondly, we added commercially available His-tagged recombinant Spike proteins from SARS-CoV-2, SARS-CoV-1 and HCoV-NL63 into HEK293T-ACE2 cells to measure the membrane binding of these Spike proteins using flow cytometry. The results showed that CDH1 overexpression reduced the binding of SARS-CoV-1 and SARS-CoV-2 Spike proteins compared to control group, whereas HCoV-NL63 Spike protein with low affinity exhibited minimal binding in both control and CDH1 overexpression groups. These data were shown as Appendix Figure S7C, D.

The ACE2-spike-cadherin 1 interaction data were obtained with recombinant proteins which might not fully model the corresponding proteins expressed in cells. It should thus be investigated whether directed expression of cadherin-1 in a cell line not only reduces SARS-CoV-2 spike driven entry but also spike binding to cells and whether KO of endogenous cadherin-1 increases spike binding.

Response: We appreciated the comment. We regulated the expression of CDH1 in HEK293T-ACE2 cells by AAV infection, and incubated them with recombinant HCoV-NL63, SARS-CoV-1, and SARS-CoV-2 Spike protein. The amount of surface-bound Spike protein was quantified via flow cytometry, and results indicated directed CDH1 overexpression reduced Spike protein binding of SARS-CoV-1 and SARS-CoV-2, and knockout of endogenous CDH1 increased Spike protein binding. However, HCoV-NL63 Spike protein showed very low affinity in both control and CDH1 regulation groups. These data were shown as Appendix Figure S7.

Minor

It should be investigated whether the negative effect of cadherin-1 on SARS-CoV-2 entry is observed for the major SARS-CoV-2 variants.

Response: We thank the reviewer for raising this important point regarding the generalizability of our findings to major SARS-CoV-2 variants. We agree that this is a critical aspect for the translational relevance of our study. Since our current experimental setup is limited to the prototypic WIV04 strain, we have added a discussion of this limitation in the revised manuscript (last paragraph of Discussion section). Future studies employing pseudoviruses or authentic viruses carrying mutations found in other circulating variants would be valuable to confirm whether the observed mechanisms are conserved across different lineages.

On several occasions patient samples were analyzed for expression of viral and cellular genes. It should be stated in the text at what time the samples were taken and what variants were circulating at that time.

Response: Four types of patient sample data were analyzed in current study. Olfactory mucosa were obtained from nine patients with post–COVID-19 hyposmia/anosmia, while control samples included olfactory tissue biopsies obtained from normosmic individuals with no history of COVID-19 and from normosmic individuals who had been previously diagnosed with COVID-19. These patients were infected with prototypic SARS-CoV-2 strain, and the human olfactory mucosa biopsies were obtained from patients at 4–16 months post-SARS-CoV-2 infection, a time frame corresponding to the post-acute phase of COVID-19 (PASC) with persistent olfactory dysfunction. (Finlay *et al.*, 2022).

In other report, samples of olfactory cleft mucosa were harvested bedside by ENT surgeons via an endoscopic endonasal approach soon after the death of the patient infected with B.1.1.7/Alpha variant(Khan *et al*, 2021).

Human bronchial epithelial cells (HBECs) were infected with SARS-CoV-2 isolate USA-WA1/2020 *in vitro* and single-cell RNA sequencing was performed at day 1, 2, and 3 post infection (Ravindra et al., 2021).

For lung cell analysis, Bronchoalveolar lavage fluid (BALF) samples were collected from COVID-19 patients infected with prototypic SARS-CoV-2 lineage during the early phase of the pandemic. 13 samples were collected from lung tissues, including 12 BALF samples and 1 pleural fluid mononuclear cell (PFMC) sample (Ren et al, 2021). The exact day post-infection on which the BALF samples were collected was not clearly specified in their manuscript.

All these were added in the manuscript.

The manuscript requires revision for grammar and style issues.

Response: We did.

Reference

Finlay JB, Brann DH, Abi Hachem R, Jang DW, Oliva AD, Ko T, Gupta R, Wellford SA, Moseman EA, Jang SS *et al* (2022) Persistent post-COVID-19 smell loss is associated with immune cell infiltration and altered gene expression in olfactory epithelium. *Sci Transl Med* 14: eadd0484

Khan M, Yoo SJ, Clijsters M, Backaert W, Vanstapel A, Speleman K, Lietaer C, Choi S, Hether TD,

Marcelis L *et al* (2021) Visualizing in deceased COVID-19 patients how SARS-CoV-2 attacks the respiratory and olfactory mucosae but spares the olfactory bulb. *Cell* 184: 5932-5949 e5915

Ravindra NG, Alfajaro MM, Gasque V, Huston NC, Wan H, Szigeti-Buck K, Yasumoto Y, Greaney AM, Habet V, Chow RD *et al* (2021) Single-cell longitudinal analysis of SARS-CoV-2 infection in human airway epithelium identifies target cells, alterations in gene expression, and cell state changes. *PLoS Biol* 19: e3001143

Ren X, Wen W, Fan X, Hou W, Su B, Cai P, Li J, Liu Y, Tang F, Zhang F *et al* (2021) COVID-19 immune features revealed by a large-scale single-cell transcriptome atlas. *Cell* 184: 1895-1913 e1819

1st Revision - Editorial Decision 9th Jun 2026

Dear Prof. Yu

Thank you for the submission of your revised manuscript to EMBO reports. We have now received the full set of referee reports that is copied below.

As you will see, all referees are very positive about the study and request only minor changes to clarify text and figures.

From the editorial side, there are also a few things that we need before we can proceed with the official acceptance of your study:

- As a standard procedure, we edit the abstract of manuscripts to make them more accessible to a general readership and to fit our word limit. Please find the suggested version below my signature.

- Please remove the figures from the manuscript file and leave the legends at the end of the manuscript.

- CRediT has replaced the traditional author contributions section because it offers a systematic machine readable author contributions format that allows for more effective research assessment. Please remove the Author Contributions section from the manuscript and use the free text boxes beneath each contributing author's name in our online systems to add specific details on the author's contribution. More information is available in our guide to authors.

- Please double-check to make sure to all relevant funding information in the manuscript is congruent with the info entered into our submission system (missing in eJP - Fudan University, Shanghai Medical College, Young investigator for clinical and scientific research team).

- Please remove the author list from the appendix file and add page numbers to the file and the table of contents.

- Please provide the appendix file at higher resolution as it was pixelated under image analysis.

- Please remove the instructions from the reagents and tools table.

- Please add the cell lines used to the reagents and tools table.

- Please include citationsfor re-used datasets and adjust the format for citation of preprints as specified in our author guidelines:
The citation in the text should be: "(preprint: NAME1 et al, YEAR)"
The citation in the reference list: "Author NAME1, Author NAME2, ... (YEAR) article title. PLATFORM NAME, doi: XXX [PREPRINT]"
Please, refer to our detailed online guidelines for further information:
https://www.embopress.org/page/journal/14602075/authorguide#referencesformat

- Please note that EMBO press papers are accompanied online by:
A) a short (2 sentences) summary of the findings and their significance,
B) 2-5 short bullet points highlighting the key results, and
C) a synopsis image in .jpg or .png format that is exactly 550 pixels wide and 300 pixels high. Please note that the text needs to be legible at the final size. Please upload this information along with your revised manuscript (the text for A and B should be provided in one separate Word file uploaded as Cover Art/Synopsis Image).

- Our production/data editors have asked you to clarify several points in the figure legends (see below). Please incorporate these changes in the manuscript and return the revised file with tracked changes with your final manuscript submission.
- Please note that the legends for figure 3; is not provided in the sequential manner (legend for figure 3E is provided before legend of figure 3D). This needs to be rectified.
- Please note that the exact p values are not provided in the legends of 3D.
- Please indicate the statistical test used for data analysis in the legends of figures 2G,H; 5F.
- Please note that the box plots need to be defined in terms of minima, maxima, centre, bounds of box and whiskers, and percentile in the legends of figures 1G; 2F; 6D,F.
- Please note that information related to n is missing in the legends of 1G; 2A,D,F,H; 6D,F.

We look forward to seeing a final version of your manuscript as soon as possible.

Kurt Weir
Editor
EMBO Reports

Olfactory dysfunction is a major symptom of COVID-19 syndrome. The critical genes contributing to SARS-CoV-2 infection are not fully understood. Here, we identify Cadherin 1 (CDH1), a hub in the interaction network of viral entry-related genes in human and mouse olfactory epithelium (OE), is coexpressed with ACE2 in sustentacular cells. CDH1 overexpression attenuates pseudoviral and authentic SARS-CoV-2 infection in ACE2-expressing HEK293T cells and human OE organoids, whereas CDH1 downregulation promotes infection. Mechanistically, CDH1 interacts with ACE2 and competes with the Spike protein for ACE2 binding. Three residues in CDH1 (W59, N168, and Q197) are critical for CDH1-ACE2 binding, and mutations at these residues weaken the interaction and enhance pseudoviral infection. In the OE of deceased COVID-19 patients, cells with higher viral load exhibit lower CDH1 expression. This inverse correlation between CDH1 expression and SARS-CoV-2 load is validated in human bronchial epithelial cells, and in lung ciliated and secretory cells. Collectively, our findings establish CDH1 as a novel restriction factor for SARS-CoV-2 infection in the olfactory system, potentially offering a target for antiviral therapy.

Referee #1:

The authors have addressed my comments.

Referee #2:

The authors have replied to all my comments. I am satisfied with the response, therefore I do not have any additional comment.

Referee #3:

The authors have partially addressed the points raised by this reviewer. The finding that CDH1 overexpression reduces while knock-down increases binding of recombinant SARS1 and SARS2 spikes to cells is important and appreciated. However, the experiments determining whether alteration of CDH1 expression modulates entry driven by SARS1, NL63 and unrelated spike proteins (specificity!) as well as the effect of CDH1 on cell entry of SARS2 variants was not examined and this important limitation should be clearly stated.

Point-by-Point Responses

Thank you for the submission of your revised manuscript to EMBO reports. We have now received the full set of referee reports that is copied below.

As you will see, all referees are very positive about the study and request only minor changes to clarify text and figures.

From the editorial side, there are also a few things that we need before we can proceed with the official acceptance of your study:

- As a standard procedure, we edit the abstract of manuscripts to make them more accessible to a general readership and to fit our word limit. Please find the suggested version below my signature.
Response: We appreciated for your editing. The new version is fine to us.

- Please remove the figures from the manuscript file and leave the legends at the end of the manuscript.
Response: We removed all figures from the manuscript file.

- CRediT has replaced the traditional author contributions section because it offers a systematic machine readable author contributions format that allows for more effective research assessment. Please remove the Author Contributions section from the manuscript and use the free text boxes beneath each contributing author's name in our online systems to add specific details on the author's contribution. More information is available in our guide to authors.
Response: We removed the Author Contributions section from the manuscript.

- Please double-check to make sure to all relevant funding information in the manuscript is congruent with the info entered into our submission system (missing in eJP - Fudan University, Shanghai Medical College, Young investigator for clinical and scientific research team).
Response: We added this funding information in eJP.

- Please remove the author list from the appendix file and add page numbers to the file and the table of contents.
Response: We removed the author list and added page numbers.

- Please provide the appendix file at higher resolution as it was pixelated under image analysis.
Response: We provided the higher resolution file.

- Please remove the instructions from the reagents and tools table.
Response: We removed the instructions.

- Please add the cell lines used to the reagents and tools table.
Response: We added.

- Please include citationsfor re-used datasets and adjust the format for citation of preprints as specified in our author guidelines:
The citation in the text should be: "(preprint: NAME1 et al, YEAR)"
The citation in the reference list: "Author NAME1, Author NAME2, ... (YEAR) article title. PLATFORM NAME, doi: XXX [PREPRINT]"
Please, refer to our detailed online guidelines for further information: https://www.embopress.org/page/journal/14602075/authorguide#referencesformat
Response: We inserted citations for re-used datasets.
The cited preprint (Ferrena A, Zhang X, Shrestha R, Zheng D, Liu W (2023) Six3 and Six6 jointly regulate the identities and developmental trajectories of multipotent retinal progenitor cells in the mouse retina. *bioRxiv*) has been published in *PloS One*. We updated the reference in the manuscript as follows, "Ferrena A, Zhang X, Shrestha R, Zheng D, Liu W (2024) Six3 and Six6 jointly control diverse target genes in multiple cell populations over developmental trajectories of mouse embryonic retinal progenitor cells. *PLoS One* 19: e0308839".

- Please note that EMBO press papers are accompanied online by:
A) a short (2 sentences) summary of the findings and their significance,
B) 2-5 short bullet points highlighting the key results, and
C) a synopsis image in .jpg or .png format that is exactly 550 pixels wide and 300 pixels high. Please note that the text needs to be legible at the final size. Please upload this information along with your revised manuscript (the text for A and B should be provided in one separate Word file uploaded as Cover Art/Synopsis Image).
Response: We uploaded all the files.

- Our production/data editors have asked you to clarify several points in the figure legends (see below). Please incorporate these changes in the manuscript and return the revised file with tracked changes with your final manuscript submission.
- Please note that the legends for figure 3; is not provided in the sequential manner (legend for figure 3E is provided before legend of figure 3D). This needs to be rectified.
Response: We rectified.
- Please note that the exact p values are not provided in the legends of 3D.
Response: We provided the exact p values in the legends of Figure 3D.
- Please indicate the statistical test used for data analysis in the legends of figures 2G,H; 5F.
Response: We indicated the statistical test.
- Please note that the box plots need to be defined in terms of minima, maxima, centre, bounds of box and whiskers, and percentile in the legends of figures 1G; 2F; 6D,F.
Response: We added in the figure legends.
- Please note that information related to n is missing in the legends of 1G; 2A,D,F,H; 6D,F.
Response: We added n number in the legends.

Referee #1:

The authors have addressed my comments.
Response: We appreciated for the comment.

Referee #2:

The authors have replied to all my comments. I am satisfied with the response, therefore I do not have any additional comment.
Response: We appreciated for the comment.

Referee #3:

The authors have partially addressed the points raised by this reviewer. The finding that CDH1 overexpression reduces while knock-down increases binding of recombinant SARS1 and SARS2 spikes to cells is important and appreciated. However, the experiments determining whether alteration of CDH1 expression modulates entry driven by SARS1, NL63 and unrelated spike proteins (specificity!) as well as the effect of CDH1 on cell entry of SARS2 variants was not examined and this important limitation should be clearly stated.
Response: We sincerely appreciated the positive evaluation of our binding assay results and the constructive feedback provided. We completely agreed with you that investigating the entry driven by SARS-CoV-1, HCoV-NL63, unrelated viral glycoproteins (as a specificity control, such as VSV-G), as well as various SARS-CoV-2 variants, is of paramount importance to comprehensively determine the specificity of CDH1-mediated regulation.

Performing these comprehensive entry assays requires a wide array of specific pseudotyped and live virus systems, which are currently beyond the scope of our available resources. We fully acknowledged this as a limitation of our current study. Following your insightful suggestion, we have explicitly stated this limitation and discussed the need for future investigations regarding viral specificity and variant cross-reactivity in the last paragraph of Discussion section.

Prof. Yiqun Yu
Eye & ENT Hospital, Fudan University
Department of Otorhinolaryngology
China

Dear Prof. Yu,

I am pleased to inform you that your manuscript has been accepted for publication in EMBO reports. Your manuscript will be processed for publication by EMBO Press. It will be copy edited and you will receive page proofs prior to publication. Please note that you will be contacted by Springer Nature Author Services to complete licensing and payment information.

You may qualify for financial assistance for your publication charges - either via a Springer Nature fully open access agreement or an EMBO initiative. Check your eligibility: https://link.springer.com/journal/44319/how-to-publish-with-us

Should you be planning a Press Release on your article, please notify the production team via this form in order to coordinate publication and release dates: https://forms.cloud.microsoft/e/eH4A8JVP6g

If you have any questions, please do not hesitate to contact the Editorial Office. Thank you for your contribution to EMBO Reports.

Yours sincerely,

Kurt Weir
Editor
EMBO Reports

>>> Please note that it is EMBO Reports policy for the transcript of the editorial process (containing referee reports and your response letter) to be published as an online supplement to each paper. If you do NOT want this, you will need to inform the Editorial Office via email immediately. More information is available here: https://link.springer.com/partners/embo-press/editorial-policies#Peer%20review

EMBO Press Author Checklist

Corresponding Author Name: Yiqun Yu	
Journal Submitted to: EMBO Reports	
Manuscript Number: EMBOR-2025-63306	

USEFUL LINKS FOR COMPLETING THIS FORM
The EMBO Journal - Author Guidelines
EMBO Reports - Author Guidelines
Molecular Systems Biology - Author Guidelines
EMBO Molecular Medicine - Author Guidelines

Reporting Checklist for Life Science Articles (updated January
This checklist is adapted from Materials Design Analysis Reporting (MDAR) Checklist for Authors. MDAR establishes a minimum set of requirements in transparent reporting in the life sciences (see Statement of Task: 10.31222/osf.io/9sm4x). Please follow the journal's guidelines in preparing your
Please note that a copy of this checklist will be published alongside your article.

Abridged guidelines for figures
1. Data
The data shown in figures should satisfy the following conditions:
- the data were obtained and processed according to the field's best practice and are presented to reflect the results of the experiments in an accurate and unbiased manner.
- ideally, figure panels should include only measurements that are directly comparable to each other and obtained with the same assay.
- plots include clearly labeled error bars for independent experiments and sample sizes. Unless justified, error bars should not be shown for technical
- if n<5, the individual data points from each experiment should be plotted. Any statistical test employed should be justified.
- Source Data should be included to report the data underlying figures according to the guidelines set out in the authorship guidelines on Data

2. Captions
Each figure caption should contain the following information, for each panel where they are relevant:
- a specification of the experimental system investigated (eg cell line, species name).
- the assay(s) and method(s) used to carry out the reported observations and measurements.
- an explicit mention of the biological and chemical entity(ies) that are being measured.
- an explicit mention of the biological and chemical entity(ies) that are altered/varied/perturbed in a controlled manner.
- the exact sample size (n) for each experimental group/condition, given as a number, not a range;
- a description of the sample collection allowing the reader to understand whether the samples represent technical or biological replicates (including how many animals, litters, cultures, etc.).
- a statement of how many times the experiment shown was independently replicated in the laboratory.
- definitions of statistical methods and measures:
  - common tests, such as t-test (please specify whether paired vs. unpaired), simple χ2 tests, Wilcoxon and Mann-Whitney tests, can be unambiguously identified by name only, but more complex techniques should be described in the methods section;
  - are tests one-sided or two-sided?
  - are there adjustments for multiple comparisons?
  - exact statistical test results, e.g., P values = x but not P values < x;
  - definition of 'center values' as median or average;
  - definition of error bars as s.d. or s.e.m.

Please complete ALL of the questions below.
Select "Not Applicable" only when the requested information is not relevant for your study.

Materials

Newly Created Materials	Information included in the manuscript?	In which section is the information available? (Reagents and Tools Table, Materials and Methods, Figures, Data Availability Section)
New materials and reagents need to be available; do any restrictions apply?	Not Applicable	

Antibodies	Information included in the manuscript?	In which section is the information available? (Reagents and Tools Table, Materials and Methods, Figures, Data Availability Section)
For **antibodies** provide the following information:<br>- Commercial antibodies: RRID (if possible) or supplier name, catalogue number and or/clone number<br>- Non-commercial: RRID or citation	Yes	Reagents and Tools Table, Materials and Methods

DNA and RNA sequences	Information included in the manuscript?	In which section is the information available? (Reagents and Tools Table, Materials and Methods, Figures, Data Availability Section)
**Short novel DNA or RNA including primers, probes**: provide the sequences.	Yes	Reagents and Tools Table, Materials and Methods

Cell materials	Information included in the manuscript?	In which section is the information available? (Reagents and Tools Table, Materials and Methods, Figures, Data Availability Section)
**Cell lines:** Provide species information, strain. Provide accession number in repository **OR** supplier name, catalog number, clone number, and/**OR** RRID.	Yes	Materials and Methods
**Primary cultures:** Provide species, strain, sex of origin, genetic modification status.	Yes	Materials and Methods
Report if the cell lines were recently **authenticated** (e.g., by STR profiling) and tested for mycoplasma contamination.	Yes	Materials and Methods

Experimental animals	Information included in the manuscript?	In which section is the information available? (Reagents and Tools Table, Materials and Methods, Figures, Data Availability Section)
**Laboratory animals or Model organisms:** Provide species, strain, sex, age, genetic modification status. Provide accession number in repository **OR** supplier name, catalog number, clone number, **OR** RRID.	Yes	Reagents and Tools Table, Materials and Methods
**Animal observed in or captured from the field:** Provide species, sex, and age where possible**.**	Not Applicable	
Please detail **housing and husbandry conditions**.	Yes	Materials and Methods

Plants and microbes	Information included in the manuscript?	In which section is the information available? (Reagents and Tools Table, Materials and Methods, Figures, Data Availability Section)
**Plants:** provide species and strain, ecotype and cultivar where relevant, unique accession number if available, and source (including location for collected wild specimens).	Not Applicable	
**Microbes:** provide species and strain, unique accession number if available, and source.	Yes	Materials and Methods

Human research participants	Information included in the manuscript?	In which section is the information available? (Reagents and Tools Table, Materials and Methods, Figures, Data Availability Section)
If collected and within the bounds of privacy constraints report on age, sex and gender or ethnicity for all study participants.	Yes	Materials and Methods

Core facilities	Information included in the manuscript?	In which section is the information available? (Reagents and Tools Table, Materials and Methods, Figures, Data Availability Section)
If your work benefited from core facilities, was their service mentioned in the acknowledgments section?	Yes	Acknowledgments

Design

Study protocol	Information included in the manuscript?	In which section is the information available? (Reagents and Tools Table, Materials and Methods, Figures, Data Availability Section)
If study protocol has been **pre-registered, provide DOI in the manuscript**. For clinical trials, provide the trial registration number **OR** cite DOI.	Not Applicable	
Report the **clinical trial registration number** (at ClinicalTrials.gov or equivalent), where applicable.	Not Applicable	

Laboratory protocol	Information included in the manuscript?	In which section is the information available? (Reagents and Tools Table, Materials and Methods, Figures, Data Availability Section)
Provide DOI OR other citation details if **external detailed step-by-step protocols** are available.	Not Applicable	

Experimental study design and statistics	Information included in the manuscript?	In which section is the information available? (Reagents and Tools Table, Materials and Methods, Figures, Data Availability Section)
Include a statement about **sample size** estimate even if no statistical methods were used.	Yes	Figure Legends
Were any steps taken to minimize the effects of subjective bias when allocating animals/samples to treatment (e.g. **randomization procedure**)? If yes, have they been described?	Not Applicable	
Include a statement about **blinding** even if no blinding was done.	Yes	Materials and Methods
Describe **inclusion/exclusion criteria** if samples or animals were excluded from the analysis. Were the criteria pre-established?<br><br>If sample or data points were omitted from analysis, report if this was due to attrition or intentional exclusion and provide justification.	Not Applicable	
For every figure, are **statistical tests** justified as appropriate? Do the data meet the assumptions of the tests (e.g., normal distribution)? Describe any methods used to assess it. Is there an estimate of variation within each group of data? Is the variance similar between the groups that are being statistically compared?	Yes	Materials and Methods, Figure Legends

Sample definition and in-laboratory replication	Information included in the manuscript?	In which section is the information available? (Reagents and Tools Table, Materials and Methods, Figures, Data Availability Section)
In the figure legends: state number of times the experiment was **replicated** in laboratory.	Yes	Figure legends
In the figure legends: define whether data describe **technical or biological replicates**.	Yes	Figure legends

Ethics

Ethics	Information included in the manuscript?	In which section is the information available? (Reagents and Tools Table, Materials and Methods, Figures, Data Availability Section)
Studies involving **human participants**: State details of **authority granting ethics approval** (IRB or equivalent committee(s), provide reference number for approval.	Yes	Materials and Methods
Studies involving **human participants**: Include a statement confirming that **informed consent** was obtained from all subjects and that the experiments conformed to the principles set out in the WMA Declaration of Helsinki and the Department of Health and Human Services Belmont Report.	Yes	Materials and Methods
Studies involving **human participants:** For publication of **patient photos**, include a statement confirming that consent to publish was obtained.	Not Applicable	
Studies involving experimental **animals**: State details of **authority granting ethics approval** (IRB or equivalent committee(s), provide reference number for approval. Include a statement of compliance with ethical regulations.	Yes	Materials and Methods
Studies involving **specimen and field samples:** State if relevant **permits** obtained, provide details of authority approving study; if none were required, explain why.	Not Applicable	

Dual Use Research of Concern (DURC)	Information included in the manuscript?	In which section is the information available? (Reagents and Tools Table, Materials and Methods, Figures, Data Availability Section)
Could your study fall under dual use research restrictions? Please check biosecurity documents and list of **select agents and toxins** (CDC): https://www.selectagents.gov/sat/list.htm	Not Applicable	
If you used a select agent, is the security level of the lab appropriate and reported in the manuscript?	Not Applicable	
If a study is subject to dual use research of concern regulations, is the name of the **authority granting approval and reference number** for the regulatory approval provided in the manuscript?	Not Applicable	

Reporting

The MDAR framework recommends adoption of discipline-specific guidelines, established and endorsed through community initiatives. Journals have their own policy about requiring specific guidelines and recommendations to complement MDAR.

Adherence to community standards	Information included in the manuscript?	In which section is the information available? (Reagents and Tools Table, Materials and Methods, Figures, Data Availability Section)
State if relevant guidelines or checklists (e.g., **ICMJE, MIBBI, ARRIVE, PRISMA**) have been followed or provided.	Not Applicable	
For **tumor marker prognostic studies,** we recommend that you follow the **REMARK** reporting guidelines (see link list at top right). See author guidelines, under 'Reporting Guidelines'. Please confirm you have followed these guidelines.	Not Applicable	
For **phase II and III randomized controlled trials**, please refer to the **CONSORT** flow diagram (see link list at top right) and submit the CONSORT checklist (see link list at top right) with your submission. See author guidelines, under 'Reporting Guidelines'. Please confirm you have submitted this list.	Not Applicable	

Data Availability

Data availability	Information included in the manuscript?	In which section is the information available? (Reagents and Tools Table, Materials and Methods, Figures, Data Availability Section)
Have **primary datasets** been deposited according to the journal's guidelines (see 'Data Deposition' section) and the respective accession numbers provided in the Data Availability Section?	Not Applicable	
Were **human clinical and genomic datasets** deposited in a public access-controlled repository in accordance to ethical obligations to the patients and to the applicable consent agreement?	Not Applicable	
Are **computational models** that are central and integral to a study available without restrictions in a machine-readable form? Were the relevant accession numbers or links provided?	Not Applicable	
If publicly available data were reused, provide the respective **data citations in the reference list**.	Yes	Materials and Methods
